# Randomized crossover trial of hand and hydrostatic casting for custom lower limb prosthetic sockets: Assessing socket comfort and fabrication time

**DOI:** 10.1371/journal.pone.0337185

**Published:** 2025-11-21

**Authors:** Stefania Fatone, Amy Gravely, Andrea Giovanni Cutti, Andrew H. Hansen, Steven A. Gard, on behalf of the Residual Limb Shape Capture Group

**Affiliations:** 1 Department of Physical Medicine and Rehabilitation, Northwestern University, Chicago, Illinois, United States of America; 2 Department of Rehabilitation Medicine, University of Washington, Seattle, Washington, United States of America,; 3 Minneapolis VA Health Care System, Minneapolis, Minnesota, United States of America; 4 INAIL, Vigorso di Budrio, Emilia Romagna, Italy; 5 Department of Family Medicine and Community Health, Department of Biomedical Engineering, University of Minnesota, Minneapolis, Minnesota, United States of America; Iran University of Medical Sciences, IRAN, ISLAMIC REPUBLIC OF

## Abstract

The aim of this study was to compare diagnostic sockets made by hand casting and standing hydrostatic pressure casting in persons with lower limb amputation. This multi-site, single-masked, randomized crossover trial (ClinicalTrials.gov NCT04141748) involved a prosthetist at each site taking one cast by hand (H) and another using hydrostatic casting (S). The process of casting, rectifying and modifying a diagnostic socket was timed in minutes. Socket comfort score (SCS) was assessed during static fitting of the diagnostic socket before (initial) and after (final) any modifications were made by the prosthetist. Difference scores for comfort and timing were calculated for each pair of casts within prosthetist. Bootstrapping methods were used to determine if the mean difference scores were significantly different from zero. Eighty participants with unilateral lower limb amputation were enrolled, with 75 completing the study. The initial SCS was significantly better in the transfemoral amputation group (TFA, n = 24) for the socket made from hand casting (H: 7.1 ± 1.9, S: 6.5 ± 2.2; p = 0.043). The final SCS was significantly better in the transtibial amputation group (TTA, n = 51) for the socket made from hydrostatic casting (H: 7.5 ± 2.0, S: 8.1 ± 1.3; p = 0.025). Total fabrication time for hydrostatic casting was significantly greater than hand casting (H: 42.1 ± 15.6, S: 48.0 ± 10.7; p = 0.001). It took significantly more time to cast (H: 10.6 ± 5.5, S: 23.7 ± 6.1; p < 0.0001) and significantly less time to rectify with hydrostatic casting (H: 23.4 ± 11.1, S: 16.6 ± 17.2; p < 0.0001). There was no difference between casting approaches for time to modify the diagnostic socket (H: 8.1 ± 7.5, S: 7.7 ± 6.6; p = 0.641). In individuals with both TTA and TFA results suggest that hydrostatic casting took more total time than hand casting to fabricate a diagnostic socket. While hand casting resulted in a significantly more comfortable socket initially for the TFA group, hydrostatic casting led to a more comfortable final socket for the TTA group.

## Introduction

The conventional lower-limb prosthetic socket fabrication process consists of residual limb shape capture via a non-weight bearing negative wrap of the residual limb with plaster or fiberglass bandages, rectification of a positive plaster mold, and, as needed, diagnostic (i.e., check) socket modifications. While this manual process of residual limb shape capture has been used for a long time and continues to be routinely used, the process has limitations, including the reliance on prosthetist experience and hand skills, which take many years to acquire [1]. Additional limitations of the manual process emerge as socket designs have evolved from those that apply pressure strategically according to anatomical structures (e.g., the Patella Tendon Bearing socket for transtibial amputation (TTA) [2] and the Ischial Containment socket for transfemoral amputation (TFA) [3]) versus those that aim to apply pressure more evenly over the entire residual limb, e.g., the Total Surface Bearing and Hydrostatic sockets for TTA [2] and the NUFlexSIS (Northwestern University Flexible Sub-Ischial Suction) socket for TFA [4,5]). While it may be possible to address some of the challenges of manual casting through use of digital scanning and rectification processes, these approaches require an *a priori* understanding of where and how much pressure should be applied to the residual limb for the fabrication of a comfortable and functional version of any particular socket design, which is currently not well-defined [1,6–9].

Since it is challenging to achieve even pressure distribution using hand casting, different approaches have been developed to apply pressure more evenly, including casting under vacuum [10] or using air-filled bladders [11], and casting using a cylinder of pressurized sand [12] or water [13]. While these casting approaches may apply pressure more evenly to the residual limb, only casting using a cylinder of pressurized sand or water does so in a weight bearing position, leading to the suggestion that casting with a cylinder of pressurized water may result in a more comfortable socket as the shape captured does not need to undergo further substantial rectifications to be suited to weight bearing socket forces [14]. A recent review proposed hydrostatic casting with water to be a more effective method of shape capture that relies less on manual manipulation of the cast, less rectification of the plaster mold, and possibly less time to achieve a comfortable diagnostic socket fit [15]. However, there was insufficient evidence available to draw conclusions regarding these claims. To date, studies of hydrostatic casting have been conducted using custom-made systems (e.g., P-Cast [16–20]). It is only recently that a system to facilitate use of hydrostatic casting in clinical practice has become commercially available (Symphonie Aqua Casting System, Romedis GmbH, Neubeuern, Germany).

While no studies have yet assessed whether hydrostatic casting is more efficient than hand casting, attempts have been made to assess whether a socket fabricated using hydrostatic casting is more comfortable than one fabricated using hand casting [15]. However, these studies did not control for a number of potentially confounding factors (e.g., socket design, socket materials, socket suspension, distal componentry, and dynamic alignment). When attempting to assess the unique contribution that the residual limb shape capture approach might make to the comfort of the socket, it is important to avoid these confounding factors. Therefore, socket comfort resulting from different casting approaches would need to be assessed at the initial static fitting of a diagnostic socket, before any modifications are made by the prosthetist to adjust socket fit and before any distal components are attached.

Hence, the aim of this study was to compare diagnostic sockets made by hand casting and standing hydrostatic pressure casting in persons with lower limb amputation on the basis of initial comfort, as well as the time required to cast, rectify, and modify a diagnostic socket. Our primary endpoint was initial socket comfort of the diagnostic socket during static fitting. It was hypothesized that compared to hand casting, (1) initial socket comfort would be greater for the diagnostic sockets fabricated by hydrostatic casting, and (2) time to fabricate a socket would be faster for hydrostatic casting. As a secondary analysis, we also assessed socket comfort after the prosthetist made modifications to the diagnostic socket.

## Materials and methods

This multi-site, single-masked, randomized crossover trial was registered on ClinicalTrials.gov (NCT04141748). A crossover design was considered appropriate given the individual shape of each limb is unique and the customized nature of prosthetic sockets. The study was approved by Institutional Review Boards at each institution (STU00210416; VAM-19–00466; 714–2019-SPER-AUSLBO-19134) as well as the Department of Defense Office of Human Research Oversight (E01091). The CONSORT extension for randomized crossover trials was used to ensure good reporting [21].

### Participants

Enrollment in the trial began on September 1, 2020, and ended on September 1, 2023, and was affected by the COVID-19 pandemic. Individuals were eligible to participate in the study if they were over 18 years of age, had a unilateral lower limb amputation at the transtibial or transfemoral level, and were currently using a prosthesis. Based on evaluation by the prosthetist and participant self-report, individuals were excluded from participation in the study if they had: an amputation for less than 1 year, poor residual limb sensation, a superficial neuroma that was painful to pressure, an open sore on the residual limb, a known silicone allergy, or a residual limb circumference or body weight that exceeded the limits of the Symphonie Aqua Casting System cylinder (i.e., > 58 cm or >170 kg for individuals with TTA and >78 cm or >170 kg for individuals with TFA). Participants were also excluded if they were unable to stand for the 4–6 minutes required for casting (e.g., individuals with bilateral amputations). Individuals with a TFA were excluded if they had a femur length less than 5 inches; which is a criteria for the NUFlexSIS socket [5].

Participants were recruited from three sites, two in the United States and one in Europe. All sites routinely conduct prosthetics research, two of the sites provide prosthetic clinical services (one site specifically to Veterans), and one site included a prosthetic and orthotic education program. The recruitment goal at each site was 20 participants with TTA and 10 with TFA, for an overall total of 90 participants. See S1 File for sample size calculations generated *a priori* of study conduct by the study statistician (A.G.).

### Interventions

For this study, the two casting approaches assessed were hand casting over a silicone liner with prosthetists’ choice of plaster or fiberglass bandages and casting with the Symphonie Aqua Casting System with plaster bandages wrapped over the same silicone liner. The Symphonie system was chosen because at the time of the study, it was the only commercially available hydrostatic casting system. Both a transtibial and transfemoral cylinder were used. Each cylinder came installed with a gauge to assess target pressure when standing with full body weight on the residual limb. Target pressure was determined using the Symphonie mobile application for those with TTA and soft tissue classification for those with TFA. The version of the system used included a Venturi pump to help fill the system with water and maintain water pressure at the desired level. Each site also had a mechanical lifter that could be used to raise and lower the cylinder to the height needed to maintain a level pelvis when standing.

### Procedures

The study required 3 visits. At the initial visit, participants were enrolled by a study coordinator at each site and written informed consent was obtained from participants. Then, demographic information was collected and included sex, age, race, ethnicity, Veteran status, height, mass, Body Mass Index (BMI), amputation level, side and etiology, residual limb length (measured with the knee flexed 30° from mid patella tendon to distal end for participants with TTA, and measured standing with the residual limb relaxed from ischial tuberosity to distal end for participants with TFA), and residual limb tissue type (categorized by the prosthetist based on manual palpation as firm, medium, or soft). The Amputee Mobility Predictor with Prosthesis (AMPPro) was administered to characterize level of mobility [22]. Finally, a distal circumference of the residual limb was taken so that a correctly sized liner could be procured. The prosthetist’s choice of liners was limited to the following: Synergy Cushion, Comfort Cushion, Dermo Cushion, and Seal-in X-TF (either conical or standard profile) (all liners from Össur, Reykjavik, Iceland).

At the second visit, casts were taken after the participant donned the silicone liner and sat quietly for 15 minutes with the residual limb supported on a stool. This time allowed residual limb fluid volume to equilibrate to the compression provided by the liner, similar to the process described by Sanders et al. [23]. To minimize variation in clinical approach, a prosthetist at each site took each cast in random order, rectified each mold in random order, and fit each diagnostic socket in random order. The three prosthetists varied in years of clinical experience as well as experience with hydrostatic casting (Table 1). The lead investigator at each site was responsible for implementing the random allocation sequence after each participant was enrolled in the study. See S1 File for calculation of the block randomization sequence generated *a priori* by the study statistician (A.G.).

**Table 1:** Prosthetist Experience at Study Commencement.

	Total Clinical Experience (years)	Experience with Hydrostatic Casting prior to Clinical Trial (years)
**Site 1**	19*	5 (passive TTA Symphonie Aqua Casting System)
**Site 2**	7*	0
**Site 3**	20**	8 (Venturi pump TTA/TFA Symphonie Aqua Casting System)

*Same prosthetist for both TTA and TFA groups.

**Different prosthetist for TTA and TFA groups.

For TTA, prosthetists were required to cast and modify a Total Surface Bearing socket design and for TFA, prosthetists were given the choice to cast and modify either an Ischial Containment or Sub-ischial socket design [5]. Between visits two and three, plaster molds were made using the casts and rectified by the prosthetist, and diagnostic sockets were vacuum formed from opaque thermoplastic.

Static fitting of the diagnostic sockets and assessment of comfort took place at the third visit after the participant donned the liner and sat quietly for 15 minutes with the residual limb supported on a stool.

### Outcome measures

Using a stopwatch, the time required to cast the participant, rectify the positive model, and fit and modify each diagnostic socket was recorded by a separate investigator who observed the prosthetist. The time it took to prepare the room for casting (i.e., ensure that all materials and tools needed were in the room) was not included in the casting time. Additionally, casting and fitting time did not include the initial 15 minutes of quiet sitting with the liner donned.

Included in the time recorded for hand casting were the following steps: apply cling wrap to the liner-clad residual limb, mark any desired landmarks, apply stockinette and plaster or fiberglass bandage to the residual limb, manually mold the cast as desired, wait until the plaster or fiberglass cured, and remove the cast.

Included in the time recorded for hydrostatic casting were the following steps: setup the Symphonie cylinder, conduct a practice run with the participant and system, empty the cylinder of water, apply cling wrap to the liner-clad residual limb, mark any desired landmarks, apply stockinette and plaster bandage to the residual limb, have the participant stand into the cylinder, apply the target water pressure, wait until the cast has cured, relieve the water pressure to allow the participant to exit the system, and remove the cast. Setup of the Symphonie cylinder included determining the target pressure, selecting the correct diameter distal cup, and adjusting the internal height of the cylinder to accommodate the participant’s residual limb length. The practice run included having the participant stand into the cylinder, establishing the correct cylinder height using a mechanical lifter (the distance from the bottom of the cylinder to the floor was measured and recorded so it could be repeated for casting), filling the cylinder with water, and practicing weight shifting onto the cylinder to assess that the participant could tolerate the target pressure when full body weight was placed on the residual limb. If the participant could not tolerate the target pressure, pressure was incrementally reduced by removing water from the cylinder until it could be tolerated. This pressure was recorded and used for casting.

During the fitting session, both the prosthetist and participant were masked as to which diagnostic socket was produced by each casting approach. This masking was accomplished by having another investigator randomly present the diagnostic sockets to the prosthetist and participant. Since prior socket design or experience with sockets may influence an individual’s assessment of comfort, we attempted to address this issue by having each participant try on each socket before assessing comfort. This allowed the participant to compare the two sockets rather than potentially considering comfort relative to their usual socket, especially for the first socket assessed. Hence, after sitting with liner donned and limb supported for 15 minutes, participants donned each socket, sat for 2 minutes with the residual limb supported by a stool, then stood for 5 minutes. Standing was accomplished by supporting the diagnostic socket on a height adjustable stand (eliminating the effects of alignment and distal components on comfort). After each participant had “experienced” both sockets, socket 1 was re-donned and the Socket Comfort Score (SCS) administered verbally by a masked-assessor (11-item response scale, 0 least comfortable socket imaginable to 10 most comfortable socket imaginable) [24]. This was then repeated for socket 2. In a study of 219 individuals with lower limb amputation, Hafner et al. [25] reported that the minimal detectable change (MDC_90_) for SCS ranged from 2.31 to 3.03 depending on administration mode (i.e., paper or electronic survey). Since our verbal mode of administration differed from those used in the Hafner et al. [25] study, we used the mean MDC_90_ of 2.73 as our reference, which was computed by Hafner et al. [25] across all modes of administration.

By consensus, study prosthetists developed a Socket Fit Evaluation Checklist (see S2 File) prior to study commencement. It was used to evaluate the fit of each check socket. If the prosthetist decided that there were modifications they could make to improve fit and comfort of the socket, those modifications were made. When the prosthetist determined that there were no further modifications to be made, the SCS was administered again for each socket. If the prosthetist determined that no modifications were required at all, the initial SCS was carried forward as the final SCS and modification time was entered as 0.

### Data analysis

Difference scores for socket comfort and fabrication timing were calculated for each pair of casts within prosthetist. Histograms of the difference scores for both socket comfort and fabrication timing indicated that these data were not normally distributed. Hence, percentile bootstrapping methods [26–28] were utilized to obtain 95% confidence intervals and p-values to determine if the mean difference scores were significantly different from 0. The difference scores overall were bootstrapped (initial SCS, final SCS, total minutes, casting minutes, rectification minutes, and diagnostic socket modification minutes) and for each subgroup of interest (i.e., TTA and TFA). We utilized SAS 9.4 (SAS Institute Inc., Cary, NC) for all analyses. For all bootstrap resampling, 5000 resamples were conducted randomly selected with replacement from the original sample. For all analyses, alpha was set at 0.05 to assess (1) differences in time (total, casting, rectifying, modifying) for each casting approach, and (2) differences in comfort between diagnostic sockets before and after modification by the prosthetist. Since the results were within groups and site is between groups, we controlled for site as a fixed effect in all our analyses.

Individual differences in SCS were also assessed with respect to the previously reported MDC_90_ [25]. Pearson’s Chi-Square and Two Sample T-Test were used as appropriate to assess differences between those participants with a difference in SCS that was more or less than the MDC_90_ [25].

## Results and discussion

**Fig 1:**
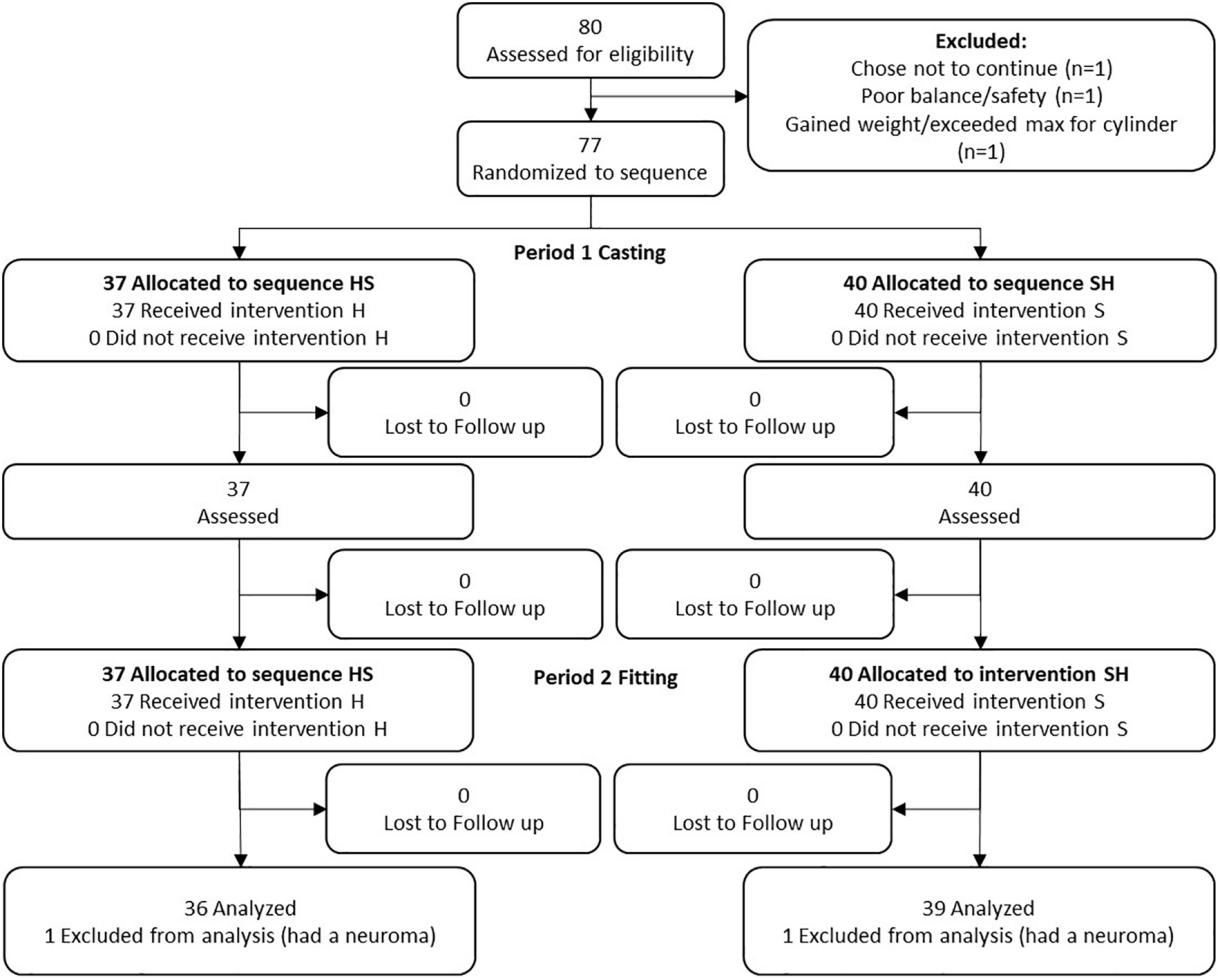
CONSORT flow diagram for crossover trials [21].

**Fig 2:**
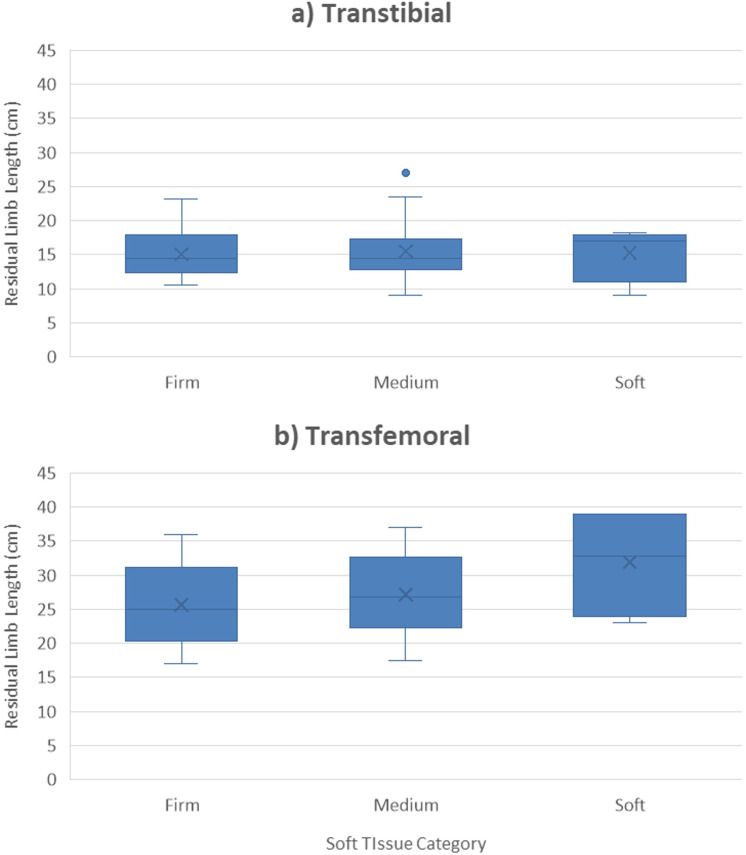
Distribution of residual limb lengths. Distribution of residual limb lengths by amputation level across soft tissue category for a) transtibial amputation group (n = 51) and b) transfemoral amputation group (n = 24).

A total of 80 participants were enrolled (site 1 = 22; site 2 = 24, site 3 = 29), representing 89% of our target enrollment. Five participants (6% of those enrolled) withdrew for a variety of reasons (Table 2, Fig 1). Of the 75 participants who completed the study, 51 (68%) had a TTA and 24 (32%) had a TFA. The mean age of all participants was 53.4 ± 16.2 years (range: 19–82 years). Sixty-six (88%) of the participants were men and 52 (69%) had an amputation due to trauma. Table 3 summarizes the participant characteristics. Fig 2 illustrates the distribution of limb lengths by amputation level across soft tissue category, illustrating that there was reasonable distribution across categories for both groups. See S1 Table for the individual characteristics of each participant.

**Table 2:** Participants Enrolled, Completed and Withdrawn.

	All Sites All/TTA/TFA	Site 1 All/TTA/TFA	Site 2 All/TTA/TFA	Site 3 All/TTA/TFA
**Recruitment Goal**	90/60/30	30/20/10	30/20/10	30/20/10
**Participants Enrolled**	80/55/25	23/15/8	26/20/6	31/20/11
**Participants Completed/Analyzed**	75/51/24	22/14/8	24/18/6	29/19/10
**Participants Withdrawn**	5	1^a^	2^b,c^	2^d^

TTA: transtibial amputation group; TFA: transfemoral amputation group.

^a^Participant did not want to continue in the study. ^b^ Participant had poor balance and was withdrawn for safety reasons. ^c^ Participant gained weight and exceeded the weight limit of the Symphonie cylinder. ^d^ Both participants were found to have an unreported distal neuroma.

**Table 3:** Summary of Participant Characteristics.

	All (n = 75)
**Age (years) (mean, SD)**	53.4 (16.2)
**Sex (n, %)**	
** Male**	66 (88%)
** Female**	9 (12%)
**Race (n, %)**	
** White**	70 (93.3%)
** Black or African American**	4 (5.3%)
** Asian**	1 (1.3%)
**Ethnicity (n, %)**	
** Hispanic or Latino**	32 (42.7)
** Not Hispanic or Latino**	43 (57.3)
**Veteran (n, %)**	
** Yes**	25 (33.3%)
** No**	50 (66.6%)
**Height (cm) (mean, SD)**	176.4 (7.8)
**Mass (kg) (mean, SD)**	85.2 (16.4)
**Body Mass Index (mean, SD)**	27.3 (4.9)
**Amputation Level (n, %)**	
** Transtibial**	51 (68%)
** Transfemoral**	24 (32%)
**Amputation Side (n, %)**	
** Left**	47 (62.7%)
** Right**	28 (37.3%)
**Amputation Etiology (n, %)**	
** Trauma**	52 (69.3%)
** Dysvascular**	8 (10.7%)
** Infection**	6 (8%)
** Cancer**	3 (4%)
** Congenital**	1 (1.3%)
** Other**	5 (6.6%)
**Time since Amputation (years) (mean, SD)**	14.5 (15.3)
**Diabetes (n, %)**	
** Yes**	10 (13.3%)
** No**	65 (86.7%)
**Residual Limb Length (cm)**	
** Transtibial (n = 51)**	15.3 ± 3.9
** Transfemoral (n = 24)**	27.6 ± 6.6
**Soft Tissue Type (n, %)**	
** Firm**	24 (32.0%)
** Medium**	43 (57.3%)
** Soft**	8 (10.7%)
**AMPPro Score (mean, SD)**	42.9 ± 4.0

SD: standard deviation. AMPPro: Amputee Mobility Predictor with Prosthesis.

Socket design was Total Surface Bearing for all participants in the TTA group, sub-ischial for 14 participants in the TFA group from sites 1 and 2, and Ischial Containment for 10 participants in the TFA group from site 3. For the TTA group, the Dermo Cushion liner was used most frequently (24/51 participants), followed by the Comfort Cushion (17/51), Synergy Cushion (9/51) and Seal-in X-TF (1/51). Mean target pressure was 0.82 ± 0.15 bar and mean actual pressure was 0.79 ± 0.15 with target pressure achieved in 28/51 participants, with 17/51 being below and 5/51 being above the target pressure. For the TFA group, the Seal-in X-TF was used most frequently in 21/24 participants, followed by the Synergy Cushion in 3/24 participants. Mean target pressure was 0.2 bar (firm) for 7/24 participants, 0.22 bar (medium) for 11/24 participants, and 0.24 bar (soft) for 6/24 participants. Mean actual pressure was 0.22 ± 0.02 bar, with mean target pressure achieved in 19/24 participants, with 2/24 being above and 3/24 being below the target pressure. See S2 Table for the individual casting details for each participant (i.e., liner type, target and actual casting pressure, socket design, etc.).

Mean initial and final SCS are shown in Table 4. Our results indicate that there were no significant differences in initial SCS for all participants (p = 0.253) or those with TTA (p = 0.050). There was a significant difference for participants with TFA (p = 0.043), where the diagnostic socket made by hand casting was more comfortable.

**Table 4:** Mean (SD) Initial and Final Socket Comfort Scores by Group.

SCS	Group	Hand Cast	Hydrostatic Cast	Parametric Difference*	Bootstrapped Difference*	95% CI	p-value
Initial	All (n = 75)	6.6 (2.3)	6.9 (1.9)	0.307	0.307	(−.227,.853)	0.253
	TTA (n = 51)	6.3 (2.5)	7.0 (1.7)	0.706	0.708	(−.020, 1.431)	0.050
	TFA (n = 24)	7.1 (1.9)	6.5 (2.2)	−0.542	−0.547	(−1.13, 0.000)	**0.043**
Final	All (n = 75)	7.6 (1.9)	7.9 (1.5)	0.293	0.288	(−.107, 0.720)	0.173
	TTA (n = 51)	7.5 (2.0)	8.1 (1.3)	0.608	0.605	(0.098, 1.157)	**0.025**
	TFA (n = 24)	7.8 (1.7)	7.4 (1.7)	−0.375	−0.378	(−.917, 0.167)	0.154

TTA: transtibial amputation group; TFA: transfemoral amputation group; CI: confidence interval; SD: standard deviation.

*Hydrostatic minus hand casting.

Bold indicates significant bootstrapped difference at p < 0.05.

Our findings demonstrate that modifications made by prosthetists to diagnostic sockets for all participants and all sockets generally improved comfort by approximately 1 point (S3 Table). Despite these improvements in comfort score with modification, there were no significant differences in final SCS for all participants (p = 0.173) or those with TFA (p = 0.154). However, there was a significant difference for participants with TTA (p = 0.025), where the diagnostic socket made using hydrostatic casting was more comfortable after modification by the prosthetist.

**Fig 3:**
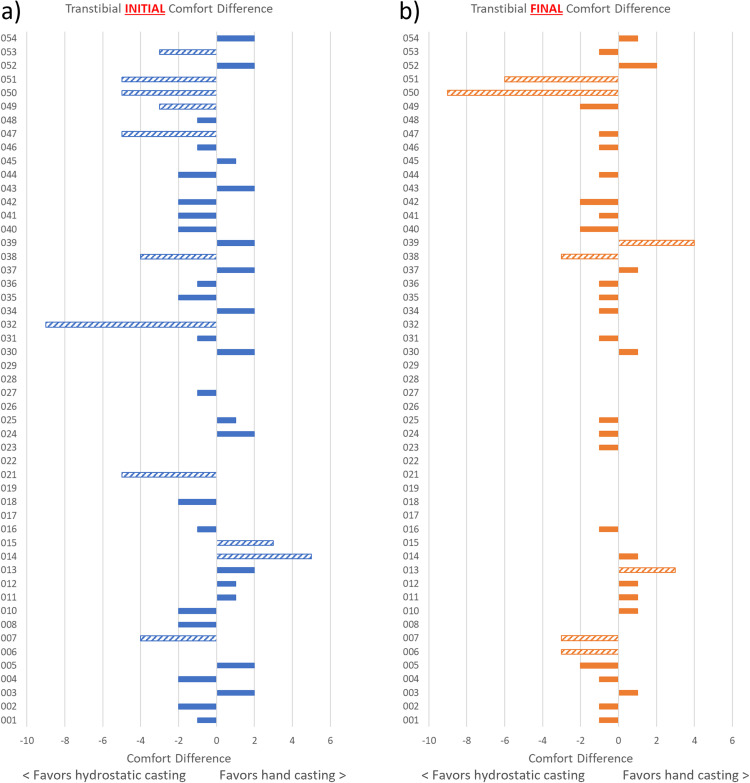
Differences in comfort score for individuals with transtibial amputation. Differences in comfort score for individual participants with transtibial amputation: a) initial difference in score (before modifications to the diagnostic socket) and b) final difference in score (after modifications to the diagnostic socket). Striped bars indicate those differences in comfort score that exceeded the MDC_90_ of 2.73. [25] Where there is no bar, the difference was 0.

**Fig 4:**
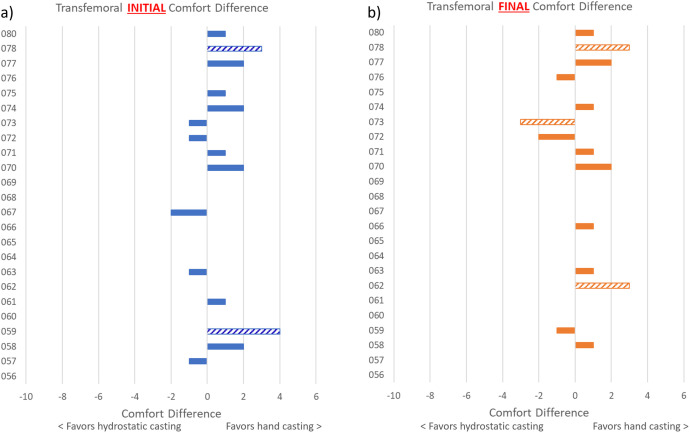
Differences in comfort score for individuals with transfemoral amputation. Differences in comfort score for individual participants with transfemoral amputation: a) initial difference in score (before modifications to the diagnostic socket) and b) final difference in score (after modifications to the diagnostic socket). Striped bars indicate those differences in comfort score that exceeded the MDC_90_ of 2.73. [25] Where there is no bar, the difference was 0.

While analyses of group differences in comfort score are important to address our hypothesis, they do not fully illustrate the range of individual responses that occurred. Differences in SCS for individual participants are illustrated in Figs 3 and 4, while the SCS for individual participants are included in the S3 Table. The range of differences in SCS was greater for those with TTA (initial score range: −9–5; final score range: −9–4) than those with TFA (initial score range: −2–4; final score range: −3–3) where negative scores indicate that comfort favors hydrostatic casting and positive scores indicate that comfort favors hand casting. For difference in SCS, the proportion of participants who favored hydrostatic casting was higher for those with TTA both initially (51% for hydrostatic casting, 33% for hand casting, and 16% no difference) and finally (49% for hydrostatic casting, 22% for hand casting, and 29% no difference), whereas for those with TFA the proportion who favored hand casting was higher both initially (21% hydrostatic casting, 42% hand casting, and 37% no difference) and finally (17% hydrostatic casting, 42% hand casting, and 42% no difference). In all cases, there were also individuals who did not have a difference in SCS between casting approaches.

More participants with TTA than TFA had SCS differences that exceeded the MDC_90_ of 2.73 [25] (TTA: 22% initial, 14% final; TFA: 8% initial, 13% final). For those with TTA, 18% of participants had an initial SCS that favored hydrostatic casting and exceeded the MDC_90_, whereas there were none among those with TFA; 4% of participants with TTA had an initial SCS that favored hand casting and exceeded the MDC_90_, compared to 8% of those with TFA. Looking at the available physical characteristics such as BMI, level of amputation, residual limb length, and soft tissue type, only level of amputation was significantly different between those who had a better initial SCS with hydrostatic casting (Table 5).

**Table 5:** Difference in physical characteristics between participants with a comfort score above and below the MDC_90_ [25].

Physical Characteristics		Total N = 75	Initial SCS < 2.73 points different for hydrostatic casting N = 66	Initial SCS > 2.73 points different for hydrostatic casting N = 9	p-value
Body Mass Index (mean, SD)		27.3 (4.9)	27.5 (4.8)	26.1 (5.1)	0.4214 ^b^
Amputation Level (n, %)	TTA	51 (68)	42 (63.6)	9 (100)	**0.0282** ^**a**^
	TFA	24 (32)	24 (36.4)	0 (0)	
Years since amputation (mean, SD)		14.5 (15.3)	15.4 (15.8)	7.9 (8.7)	0.1700 ^b^
Residual Limb Length (cm, mean, SD)		19.2 (7.5)	19.7 (7.8)	15.7 (4.0)	0.1345 ^b^
Soft Tissue Type (n, %)	Firm	24 (32.0)	19 (28.8)	5 (55.6)	0.2463 ^a^
	Medium	43 (57.3)	40 (60.6)	3 (33.3)	
	Soft	8 (10.7)	7 (10.6)	1 (11.1)	

SD: standard deviation; TTA: transtibial amputation; TFA: transfemoral amputation; SCS: socket comfort score.

^a^Pearson’s Chi-Square; ^b^ Two Sample T-Test.

Results for fabrication time are shown in Table 6. Fabrication times for individual participants are included in the S4 Table. Our results indicate that for all participants total fabrication time (p = 0.001), time to cast (p < 0.0001), and time to rectify (p < 0.0001) were significantly different between casting approaches. While it took significantly more time to cast with hydrostatic casting, and significantly less time to rectify with hydrostatic casting, total fabrication time for hydrostatic casting took more time than hand casting. There was no difference between casting approaches for time to modify the diagnostic socket (p = 0.641). Differences in timing were the same for participants with TTA and TFA.

**Table 6:** Mean (SD) Fabrication Time by Group.

Time (min)	Group	Hand Cast	Hydrostatic Cast	Parametric Difference*	Bootstrapped Difference*	95% CI	p-value
Total	All (n = 75)	42.1 (15.6)	48.0 (10.7)	5.93	5.94	(2.74, 9.10)	**0.001**
	TTA (n = 51)	43.9 (16.7)	48.6 (11.1)	4.74	4.76	(0.76, 8.71)	**0.020**
	TFA (n = 24)	38.2 (12.1)	46.6 (9.9)	8.45	8.43	(3.32, 13.59)	**0.001**
Casting	All (n = 75)	10.6 (5.5)	23.7 (6.1)	13.11	13.11	(11.45, 14.71)	**<0.0001**
	TTA (n = 51)	10.9 (5.7)	24.3 (6.2)	13.38	13.39	(11.63, 15.20)	**<0.0001**
	TFA (n = 24)	9.9 (5.2)	22.5 (5.9)	12.55	12.55	(9.02, 15.99)	**<0.0001**
Rectification	All (n = 75)	23.4 (11.1)	16.6 (7.2)	−6.83	−6.83	(−9.05, −4.58)	**<0.0001**
	TTA (n = 51)	24.6 (12.1)	16.6 (7.5)	−8.03	−8.03	(−11.0, −4.95)	**<0.0001**
	TFA (n = 24)	21.0 (8.4)	16.7 (6.8)	−4.28	−4.29	(−6.19, −2.61)	**<0.0001**
Modification	All (n = 75)	8.1 (7.5)	7.7 (6.6)	−0.35	−0.34	(−1.185, 1.115)	0.641
	TTA (n = 51)	8.4 (6.9)	7.8 (6.1)	−0.61	−0.61	(−2.28, 1.013)	0.468
	TFA (n = 24)	7.3 (8.9)	7.5 (7.6)	0.18	0.17	(−3.04, 2.943)	0.907

TTA: transtibial amputation group; TFA: transfemoral amputation group; CI: confidence interval

*Hydrostatic minus hand casting.

Bold indicates significant bootstrapped difference at p < 0.05.

The aim of this comparative effectiveness clinical trial was to assess whether initial and final comfort of the diagnostic socket differed between hand casting and standing hydrostatic pressure casting in persons with lower limb amputation, as well as whether time to cast, rectify, and modify a diagnostic socket differed between casting approaches. Overall, our findings suggest that initial socket comfort was better for hand casting for the group with TFA, while final socket comfort was better for hydrostatic casting for the group with TTA. Additionally, our findings suggest that there was a significant difference in total fabrication time, with casting time being greater for hydrostatic casting. While rectification took less time for hydrostatic casting, the difference was not sufficient to affect total time.

When considered together, comfort results at the group and individual level suggest that hydrostatic casting of Total Surface Bearing sockets may have some merit over hand casting for individuals with TTA. While group results did not suggest a difference in initial SCS between diagnostic sockets made using hand and hydrostatic casting for participants with TTA, the 95% confidence interval of −0.020 to 1.431 (where positive values favor the hydrostatic socket) and the p-value (p = 0.05) were right on the threshold of favoring hydrostatic casting. Furthermore, Fig 3 illustrates that initial comfort was clinically different in favor of hydrostatic casting for 9 participants, and in favor of hand casting for 2 participants. Final comfort was still clinically different in favor of hydrostatic casting for 5 participants versus in favor of hand casting for 2 participants.

This study was conducted during the COVID-19 pandemic, affecting our recruitment rate. Our sample size calculations indicated that we needed 54 individuals with TTA to detect a 1-point difference in comfort score and we completed the study with 51 and mean comfort differences between sockets were less than 1 point. It is possible that if we had achieved our *a priori* sample size, the initial comfort results would have favored hydrostatic casting. However, since we did find a significant difference in comfort in the TTA group with the same sample size after modifications, it is possible that sample size was not the issue but rather that modifications to the socket are required to achieve comfort. Modifications were most frequently made to adjust the socket trimlines for sockets made using both hand and hydrostatic casting.

The opposite appears to be the case for individuals with TFA: when considered together, comfort results at the group and individual level suggest that hand casting may have merit over hydrostatic casting. However, the extent to which it does so seems to vary by socket design. Two socket designs were used at the prosthetists’ discretion for the TFA group and different sites chose one or the other design based on their typical clinical practice or ease with the Symphonie system. While this reflects real world practice, we acknowledge that it introduces variability. Two of the 3 sites chose to cast for a Sub-ischial socket (where initial socket comfort favored hand casting in 3/14 participants; favored hydrostatic casting in 3/14; and did not change for 8/14) and one site chose to cast for an Ischial Containment socket (where initial socket comfort favored hand casting in 7/10 participants; favored hydrostatic casting in 2/10; and did not change for 1/10). Since initial comfort for the Ischial Containment socket favored hand casting in the majority of participants, it may be more sensitive to casting approach than for the Sub-ischial socket where the majority of participants demonstrated no difference in initial comfort. While not initially planned, we conducted an additional analysis by socket design for TFA participants to further explore these observations. We compared the SCS of TFA participants from site 3 who received Ischial Containment sockets (n = 10) to participants from sites 1 and 2 combined who received Sub-ischial sockets (n = 14). The results were not significant (p = 0.19). These findings should be considered cautiously given the small number of participants who were cast for each socket design.

It is possible that the approach to determining target pressure during hydrostatic casting should be further considered as perhaps it might also affect initial socket comfort and need to vary for different socket designs. While the Symphonie mobile application provides a method of calculating target pressure for individuals with TTA, there was no such application available at the time of the study for those with TFA, which is a distinct limitation. Given this absence, we utilized a method based on soft tissue categorization of a group of 64 participants with TFA developed for casting Ischial Containment sockets in a pilot study conducted by one of our sites prior to the start of the trial [29]. However, evaluation of a standardized protocol for calculating target pressure for hydrostatic casting of individuals with TFA is needed.

We tried to explore why hydrostatic casting resulted in a socket that was more comfortable than hand casting for some individuals but were unable to do so using our limited data set of participant physical characteristics. A previously published study comparing comfort of definitive sockets fabricated by hand and hydrostatic casting [17] reported that their socket comfort and preference results were similar regardless of participant limb length, presence of limb anomalies, or previous socket type worn. Future studies should seek to better assess variables characterizing the physical presentation of participants to better understand for whom hydrostatic casting is most appropriate.

Our results suggest that the difference in total time between casting approaches lies between two and nine minutes. A difference on the lower end of this range may not be clinically impactful but a difference on the larger end of this range may affect clinical efficiency. We attribute our longer hydrostatic casting time results to both setup of the cylinder for casting and testing of the system before casting. While this protocol is prudent when using the system with individuals for the first time, if a prosthetist were to skip testing of the system with every patient, the time to cast may be substantially faster. It should be noted that other investigators have reported using their own custom-made hydrostatic casting system [16–19,30,31] with varying levels of target pressure (e.g., 50% or 100% body weight when specified) [17,19,20,31]. It is possible that other systems are set up differently and that these results are unique to this system and may not generalize to all hydrostatic casting systems.

To our knowledge this is the first study to prospectively assess comfort and timing of Total Surface Bearing diagnostic sockets made using the Symphonie Aqua Casting System and hand casting. It is possible that assessing socket comfort of the initial static fitting of a diagnostic socket, on a height-adjustable stand prior to the attachment and alignment of distal components, may be perceived as a limitation. However, this condition was chosen deliberately as it allowed control of confounding by suspension mechanism, componentry, and alignment. While we think it is the appropriate endpoint to address the contribution that casting method may make to socket comfort, we recognize that it is not a final clinical endpoint in terms of socket comfort [17]. In one prior study that attempted to compare casting methods, the authors chose a different endpoint. In that study, the PCAST hydrostatic casting system (originally described by Lee et al. [16]) was used to fabricate transtibial sockets that were compared in random order to Patella Tendon Bearing (PTB) sockets made using hand casting [17]. After 15 minutes acclimation in these definitive sockets with components attached no difference in socket comfort was found [17].

While there was considerable variation in the prosthetists’ years of clinical experience and experience with the Symphonie Aqua Casting System, it did not affect the results given the paired nature of the data (i.e., within subjects). That does not mean that clinician experience did not contribute variability to the potential quality of the casts taken and sockets made, but rather that the bias inherent between prosthetists was attenuated by the within prosthetist paired study design. Level of prosthetist experience influenced the choice of socket design at site 2: since the prosthetist had no prior experience with hydrostatic casting, he chose to implement the Sub-ischial socket with TFA participants, as it is both a more straightforward socket design to cast and rectify by hand as well as with the hydrostatic casting system. Since we were unable to assess differences in comfort between hand and hydrostatic casting across prosthetists with varying levels of experience, it would be interesting for a future study to explore how hydrostatic casting by novice prosthetists compares to hand casting by experienced prosthetists of the same participant. Such a study would help us better understand whether hydrostatic casting does indeed require less skill and experience to achieve a socket with comfort similar to that of a prosthetist with many years of skill and experience casting by hand. Additionally, while hydrostatic casting took more time overall, the reduced rectification time and the simpler rectifications required for hydrostatic casting may be clinically meaningful, especially when comparing novice and experienced clinicians. This would also be interesting for a future study to explore further.

Another potentially confounding factor for future studies to explore is the amount of weight bearing on the cylinder during casting. When specified, other studies of hydrostatic casting reported either 50% [17,19,20] or 100% loading [31] of the residual limb during casting. In this study we used the Symphonie mobile application to determine a target pressure with inputs of weight, circumference at mid patella tendon, soft tissue classification (soft, medium, firm), and K-level for individuals with TTA and soft tissue classification alone for individuals with TFA. This approach resulted in a target pressure that was achieved 55% of the time for participants with TTA (with 33% being below the target pressure due to participants expressing they felt excessive and/or uncomfortable pressure during the initial practice test) and 80% of the time for participants with TFA (with 12% being below the target pressure). Another consideration is that the speed with which the participant shifted weight onto the residual limb during casting may have contributed to different experiences of the target pressure. This weight shifting speed may have affected the cast shape obtained given that it may have occurred at different points in the plaster curing process [32] and this may in turn have affected the difference in socket comfort achieved. Future studies should explore the extent to which these variables affect the comfort of sockets made using hydrostatic casting. Furthermore, they highlight that hydrostatic casting requires an investment of time and effort to develop skills to perform consistently.

Another possible issue affecting our results is that the process of taking two casts in a single study visit may have altered residual limb volume. Casting in a randomized order should have helped attenuate the effect of this issue on socket comfort to some extent.

## Conclusions

The results of this multi-center clinical trial in individuals with both TTA and TFA suggest that hydrostatic casting using the Symphonie Aqua Casting System took more total time than hand casting to fabricate a diagnostic socket. While hand casting resulted in a significantly more comfortable socket initially for the group with TFA, hydrostatic casting led to a more comfortable final socket for the group with TTA. Individual differences in response to casting deserve further research.

## Supporting information

S1 Table Individual Participant Characteristics. (PDF)

S2 Table Individual Casting Details. (PDF)

S3 Table Initial and Final Comfort Scores and Differences in Comfort Scores for all Participants. (PDF)

S4 Table Fabrication Time and Casting Approach. (PDF)

S1 File Sample Size Calculation and Randomization Sequence. (PDF)

S2 File Socket Fit Evaluation Checklist. (PDF)

S1 Protocol IRB Approved Protocol. (PDF)

S1 Checklist CONSORT Extension for Randomized Crossover Trials Checklist. (PDF)
