# Supplementary material for: Randomized crossover trial of hand and hydrostatic casting for custom lower limb prosthetic sockets: Assessing socket comfort and fabrication time
Source: PLoS One. 2025 Nov 21;20(11):e0337185. doi: 10.1371/journal.pone.0337185 (PMC12637896; doi:10.1371/journal.pone.0337185)
Supplement: S1 Table — (PDF) [file pone.0337185.s001.pdf]

S1 Table. Individual Participant Characteristics.

Subject ID	Sex (M/F)	Age (years)	Mass (kg)	Height (cm)	BMI	Amputation				Residual Limb Length (cm)	Tissue Type	AMPPro Score
						Side (R/L)	Level (TTA/TFA)	Etiology	Time Since (years)			
001	M	56	105	190	28.9	L	TTA	Trauma	34	10.5	Firm	46
002	M	24	97	188	27.6	L	TTA	Trauma	4	16.0	Firm	46
003	M	29	82	170	28.3	L	TTA	Dysvascular	5	10.8	Firm	42
004	M	59	95	173	31.8	R	TTA	Cancer	5	21.0	Firm	45
005	M	31	70	175	23.0	L	TTA	Infection	4	12.0	Medium	43
006	M	66	91	183	27.2	L	TTA	Trauma	6	13.0	Firm	43
007	M	49	93	166	33.8	R	TTA	Congenital	30	17.5	Firm	42
008	F	62	88	163	33.1	L	TTA	Other	17	12.5	Medium	45
009 (w)	F	45	89	154	37.4	L	TTA	Dysvascular	7	14.5	Medium	44
010	M	78	97	183	29.0	L	TTA	Cancer	15	19.4	Medium	43
011	F	60	61	173	20.3	R	TTA	Trauma	24	13.0	Medium	46

Subject ID	Sex (M/F)	Age (years)	Mass (kg)	Height (cm)	BMI	Amputation				Residual Limb Length (cm)	Tissue Type	AMPPro Score
						Side (R/L)	Level (TTA/TFA)	Etiology	Time Since (years)			
012	M	48	104	175	34.0	L	TTA	Infection	3	13.4	Medium	41
013	F	46	55	172	18.5	L	TTA	Trauma	19	10.5	Firm	46
014	M	65	106	175	34.6	L	TTA	Trauma	43	19.0	Firm	42
015	M	64	86	169	29.9	L	TTA	Trauma	27	13.5	Medium	43
016	F	35	99	168	35.3	L	TTA	Trauma	2	16.0	Medium	45
017	M	73	86	180	26.5	L	TTA	Trauma	41	27.0	Medium	42
018	M	69	99	187	28.4	L	TTA	Trauma	2	16.5	Medium	38
019	M	74	98	165	35.8	L	TTA	Trauma	52	9.0	Medium	40
020 (w)	M	72	113	193	30.4	L	TTA	Infection	3	14.0	Soft	NR
021	M	49	104	173	35.0	L	TTA	Trauma	8	19.0	Medium	47
022	M	72	107	185	31.0	L	TTA	Infection	2	14.5	Medium	42

Subject ID	Sex (M/F)	Age (years)	Mass (kg)	Height (cm)	BMI	Amputation				Residual Limb Length (cm)	Tissue Type	AMPPro Score
						Side (R/L)	Level (TTA/TFA)	Etiology	Time Since (years)			
023	M	63	102	188	28.9	R	TTA	Infection	3	13.5	Medium	39
024	M	68	102	185	29.7	L	TTA	Trauma	30	14.0	Medium	45
025	M	62	117	171	39.7	R	TTA	Infection	1	11.0	Medium	32
026	M	66	91	173	30.4	R	TTA	Other	3	13.0	Medium	41
027	M	65	102	178	32.3	R	TTA	Other	9	16.5	Medium	44
028	M	75	88	178	27.8	R	TTA	Trauma	52	9.0	Soft	45
029	M	59	101	180	31.1	R	TTA	Trauma	13	12.0	Medium	37
030	M	73	93	185	27.0	R	TTA	Trauma	52	21.0	Medium	45
031	M	73	73	173	24.5	R	TTA	Dysvascular	5	16.0	Medium	42
032	M	75	68	178	21.5	R	TTA	Dysvascular	6	15.0	Firm	28
033 (w)	M	61	173	183	51.8	L	TTA	Trauma	22	15.0	Medium	39

Subject ID	Sex (M/F)	Age (years)	Mass (kg)	Height (cm)	BMI	Amputation				Residual Limb Length (cm)	Tissue Type	AMPPro Score
						Side (R/L)	Level (TTA/TFA)	Etiology	Time Since (years)			
034	M	49	95	180.	29.3	R	TTA	Trauma	9	18.0	Medium	47
035	M	76	113	183	33.9	R	TTA	Infection	2	17.0	Soft	42
036	M	48	87	180	26.9	L	TTA	Trauma	17	16.3	Medium	46
037	M	59	78	186	22.5	L	TTA	Trauma	2	12.5	Firm	46
038	M	29	75	190	20.8	L	TTA	Trauma	3	12.0	Firm	46
039	M	49	108	175	35.3	R	TTA	Trauma	5	20.0	Firm	46
040	M	40	68	179	21.2	R	TTA	Trauma	4	13.5	Medium	46
041	M	40	84	175	27.4	R	TTA	Dysvascular	37	12.5	Firm	46
042	M	33	86	180	26.5	L	TTA	Trauma	7	15.3	Firm	46
043	M	23	60	180	18.5	L	TTA	Trauma	7	21.0	Medium	46
044	M	34	87	168	30.8	L	TTA	Trauma	18	17.0	Soft	46
045	M	64	76	178	24.0	L	TTA	Trauma	44	15.0	Medium	46
046	F	57	61	165	22.4	R	TTA	Trauma	3	23.5	Medium	46
047	M	37	55	174	18.2	R	TTA	Trauma	7	11.5	Medium	46
048	M	62	83	182	25.1	L	TTA	Trauma	14	15.0	Medium	46
049	M	63	88	175	28.7	L	TTA	Trauma	3	12.5	Medium	46

Subject ID	Sex (M/F)	Age (years)	Mass (kg)	Height (cm)	BMI	Amputation				Residual Limb Length (cm)	Tissue Type	AMPPro Score
						Side (R/L)	Level (TTA/TFA)	Etiology	Time Since (years)			
050	M	40	74	170	25.6	L	TTA	Trauma	9	23.2	Firm	46
051	M	48	97	185	28.3	L	TTA	Other	3	12.5	Firm	43
052	M	62	94	187	26.9	L	TTA	Trauma	35	14.0	Firm	46
053	M	37	74	180	22.8	L	TTA	Trauma	2	18.2	Soft	46
054	M	58	76	165	27.9	L	TTA	Trauma	5	16.0	Firm	46
055 (w)	M	56	99	178	31.2	L	TTA	Trauma	5	20.0	Medium	46
056	M	36	112	190	31.0	L	TFA	Cancer	NR	36.0	Firm	40
057	M	65	98	189	27.6	L	TFA	Trauma	7	28.0	Firm	43
058	M	64	92	180	28.4	L	TFA	Dysvascular	2	23.0	Medium	39
059	F	51	59	171	20.2	L	TFA	Trauma	32	27.0	Medium	43
060	F	69	70	165	25.7	L	TFA	Dysvascular	11	19.0	Medium	30*
061	M	49	72	172	24.3	L	TFA	Trauma	28	17.0	Firm	43
062	M	57	91	179	28.3	L	TFA	Other	3	22.0	Firm	44
063	M	51	109	191	30.0	L	TFA	Trauma	49	17.5	Medium	43

Subject ID	Sex (M/F)	Age (years)	Mass (kg)	Height (cm)	BMI	Amputation				Residual Limb Length (cm)	Tissue Type	AMPPro Score
						Side (R/L)	Level (TTA/TFA)	Etiology	Time Since (years)			
064	M	73	91	170	31.3	R	TFA	Dysvascular	8	26.0	Medium	32
065	M	82	98	175	31.9	L	TFA	Dysvascular	2	39.0	Soft	32
066	M	62	84	170	29.0	R	TFA	Trauma	5	32.5	Medium	33
067	M	40	63	175	20.4	R	TFA	Trauma	20	33.0	Medium	44
068	M	67	83	165	30.3	R	TFA	Trauma	45	37.0	Medium	38
069	M	72	81	170	28.0	R	TFA	Trauma	43	39.0	Soft	38
070	F	33	52	159	20.6	R	TFA	Trauma	2	23.0	Soft	43
071	M	22	59	175	19.3	R	TFA	Trauma	1	25.0	Medium	46
072	M	44	94	178	29.7	R	TFA	Trauma	14	29.5	Firm	45
073	M	26	82	180	25.3	L	TFA	Trauma	8	21.5	Firm	46
074	F	19	40	155	16.6	L	TFA	Trauma	1	20.0	Medium	46
075	M	53	85	178	26.8	R	TFA	Trauma	16	26.5	Medium	46
076	M	19	69	183	20.6	L	TFA	Trauma	1	33.5	Medium	43
077	M	47	77	182	23.2	L	TFA	Trauma	2	32.0	Medium	46

Subject ID	Sex (M/F)	Age (years)	Mass (kg)	Height (cm)	BMI	Amputation				Residual Limb Length (cm)	Tissue Type	AMPPro Score
						Side (R/L)	Level (TTA/TFA)	Etiology	Time Since (years)			
078	M	46	68	172	23.0	L	TFA	Trauma	3	28.0	Medium	39
079 (w)	M	65	66	173	22.1	L	TFA	Trauma	41	25.5	Soft	45
080	M	60	85	170	29.4	L	TFA	Trauma	15	26.5	Soft	40
**All**	**Mean**	**53.4**	**85.2**	**176.4**	**27.3**				**14.5**	**19.2**		**42.9**
	**SD**	**16.2**	**16.4**	**7.8**	**4.9**				**15.3**	**7.5**		**4.0**
**TTA**	**Mean**	**54.8**	**87.8**	**177.2**	**28.0**				**14.8**	**15.3**		**43.6**
	**SD**	**15.3**	**15.3**	**7.2**	**5.0**				**15.5**	**3.9**		**3.7**
**TFA**	**Mean**	**50.3**	**79.8**	**174.8**	**25.9**				**13.8**	**27.6**		**41.4**
	**SD**	**17.9**	**17.7**	**9.0**	**4.4**				**15.3**	**6.6**		**4.4**

Gray shading and (w) indicates subject withdrew or was withdrawn.
BMI: Body Mass Index; F: female; M: male; R: right; L: left: TTA: transtibial amputation; TFA: transfemoral amputation; NR: not reported; SD standard deviation.
**Participant assessed with AMPnoPro as they attended the first visit without their prosthesis.*
Mean and standard deviation are shown for participants who completed the study.
