# Supplementary material for: Randomized crossover trial of hand and hydrostatic casting for custom lower limb prosthetic sockets: Assessing socket comfort and fabrication time
Source: PLoS One. 2025 Nov 21;20(11):e0337185. doi: 10.1371/journal.pone.0337185 (PMC12637896; doi:10.1371/journal.pone.0337185)
Supplement: S2 Table — (PDF) [file pone.0337185.s002.pdf]

S2 Table. Individual Casting Details.

Subject ID	Liner Type	Liner Profile	Target Pressure (bar)	Actual Pressure (bar)	Socket Type
001	Össur Comfort Cushion	n/a	0.80	0.60	TSB
002	Össur Comfort Cushion	n/a	0.82	0.50 to 0.60	TSB
003	Össur Synergy Cushion	n/a	0.82	0.60 to 0.70	TSB
004	Össur Comfort Cushion	n/a	0.94	0.60	TSB
005	Össur Comfort Cushion	n/a	0.78	0.70	TSB
006	Össur Synergy Cushion	n/a	0.85	0.85	TSB
007	Össur Comfort Cushion	n/a	0.78	0.75	TSB
008	Össur Comfort Cushion	n/a	0.95	0.90	TSB
010	Össur Comfort Cushion	n/a	0.87	0.80	TSB
011	Össur Comfort Cushion	n/a	0.79	0.79	TSB
012	Össur Comfort Cushion	n/a	0.94	0.90	TSB
013	Össur Comfort Cushion	n/a	0.69	0.69	TSB
014	Össur Comfort Cushion	n/a	1.00	0.70	TSB
015	Össur Comfort Cushion	n/a	0.74	0.75	TSB
016	Össur Synergy Cushion	n/a	0.77	0.77	TSB
017	Össur Dermo Cushion	n/a	0.85	0.85	TSB
018	Össur Dermo Cushion	n/a	0.98	0.65	TSB
019	Össur Dermo Cushion	n/a	0.92	0.92	TSB
021	Össur Synergy Cushion	n/a	1.03	1.00	TSB
022	Össur Dermo Cushion	n/a	NR	NR	TSB
023	Össur Dermo Cushion	n/a	0.84	0.84	TSB
024	Össur Synergy Cushion	n/a	0.85	0.85	TSB
025	Össur Dermo Cushion	n/a	0.94	0.80	TSB
026	Össur Dermo Cushion	n/a	0.70	0.70	TSB
027	Össur Synergy Cushion	n/a	0.83	0.83	TSB
028	Össur Dermo Cushion	n/a	0.90	0.90	TSB
029	Össur Dermo Cushion	n/a	0.94	0.90	TSB
030	Össur Dermo Cushion	n/a	0.77	0.77	TSB
031	Össur Dermo Cushion	n/a	0.74	0.70	TSB
032	Össur Synergy Cushion	n/a	0.69	0.69	TSB
034	Össur Dermo Cushion	n/a	0.76	0.76	TSB
035	Össur Synergy Cushion	n/a	0.93	0.93	TSB
036	Össur Dermo Cushion	n/a	0.89	0.89	TSB
037	Össur Dermo Cushion	n/a	0.86	0.86	TSB
038	Össur Dermo Cushion	n/a	0.75	0.75	TSB
039	Össur Dermo Cushion	n/a	0.89	0.89	TSB
040	Össur Dermo Cushion	n/a	0.74	0.74	TSB
041	Össur Dermo Cushion	n/a	0.74	0.70	TSB
042	Össur Dermo Cushion	n/a	0.76	0.76	TSB
043	Össur Dermo Cushion	n/a	0.74	0.74	TSB
044	Össur Dermo Cushion	n/a	1.12	1.00	TSB
045	Össur Comfort Cushion	n/a	0.89	0.89	TSB
046	Össur Comfort Cushion	n/a	0.83	0.90	TSB
047	Össur Comfort Cushion	n/a	0.73	0.75	TSB
048	Össur Comfort Cushion	n/a	0.83	0.83	TSB
049	Össur Seal-in X-TF	n/a	0.80	0.80	TSB
050	Össur Synergy Cushion	n/a	0.78	0.78	TSB
051	Össur Dermo Cushion	n/a	0.85	0.90	TSB
052	Össur Dermo Cushion	n/a	0.85	0.85	TSB
053	Össur Comfort Cushion	n/a	0.90	0.90	TSB
054	Össur Dermo Cushion	n/a	0.82	0.84	TSB
056	Össur Seal-in X-TF	Standard	Firm (0.20 bar)	0.20	SI
057	Össur Synergy Cushion	Standard	Medium (0.22 bar)	0.22	SI
058	Össur Synergy Cushion	Standard	Medium (0.22 bar)	0.22	SI

Subject ID	Liner Type	Liner Profile	Target Pressure (bar)	Actual Pressure (bar)	Socket Type
059	Össur Seal-in X-TF	Standard	Soft (0.24 bar)	0.24	SI
060	Össur Seal-in X-TF	Standard	Medium (0.22 bar)	0.22	SI
061	Össur Seal-in X-TF	NR	Firm (0.20 bar)	0.20	SI
062	Össur Seal-in X-TF	Standard	Medium (0.22 bar)	0.22	SI
063	Össur Seal-in X-TF	Conical	Firm (0.20 bar)	0.22	SI
064	Össur Seal-in X-TF	NR	Soft (0.24 bar)	0.24	SI
065	Össur Seal-in X-TF	Conical	Soft (0.24 bar)	0.24	SI
066	Össur Seal-in X-TF	Standard	Soft (0.24 bar)	0.24	SI
067	Össur Seal-in X-TF	Standard	Medium (0.22 bar)	0.22	SI
068	Össur Seal-in X-TF	Standard	Medium (0.22 bar)	0.22	SI
069	Össur Seal-in X-TF	Standard	Soft (0.24 bar)	0.24	SI
070	Össur Seal-in X-TF	Conical	Medium (0.22 bar)	0.22	IC
071	Össur Seal-in X-TF	Conical	Firm (0.20 bar)	0.20	IC
072	Össur Seal-in X-TF	Conical	Firm (0.20 bar)	0.20	IC
073	Össur Seal-in X-TF	Conical	Firm (0.20 bar)	0.20	IC
074	Össur Seal-in X-TF	Conical	Medium (0.22bar)	0.22	IC
075	Össur Seal-in X-TF	Conical	Firm (0.20 bar)	0.20	IC
076	Össur Synergy Cushion	Conical	Medium (0.22 bar)	0.18	IC
077	Össur Seal-in X-TF	Conical	Medium (0.22 bar)	0.20	IC
078	Össur Seal-in X-TF	Conical	Medium (0.22 bar)	0.20	IC
080	Össur Seal-in X-TF	Conical	Soft (0.24 bar)	0.30	IC

n/a = not applicable; NR = not reported; TSB = Total Surface Bearing; SI = Sub-ischial; IC = Ischial Containment.
