# Supplementary material for: Randomized crossover trial of hand and hydrostatic casting for custom lower limb prosthetic sockets: Assessing socket comfort and fabrication time
Source: PLoS One. 2025 Nov 21;20(11):e0337185. doi: 10.1371/journal.pone.0337185 (PMC12637896; doi:10.1371/journal.pone.0337185)
Supplement: S3 Table — (PDF) [file pone.0337185.s003.pdf]

S3 Table. Initial and Final Comfort Scores and Differences in Comfort Scores for all Participants.

Subject ID	Initial Comfort Score		Final Comfort Score		Difference between initial comfort scores (hand minus hydrostatic cast)	Difference between final comfort scores (hand minus hydrostatic cast)	Difference between initial and final hand casting	Difference between initial and final hydrostatic casting
	Hand Casting	Hydrostatic Casting	Hand Casting	Hydrostatic Casting				
001	6	7	7	8	-1	-1	1	1
002	0	2	6	7**	-2	-1	6	5
003	7	5	9	8	2	1	2	3
004	5	7	8	9	-2	-1	3	2
005	8	6	8	10	2	-2	0	4
006	4	4	3	6	0	-3	-1	2
007	3	7	5	8	-4	-3	2	1
008	6	8	9	9	-2	0	3	1
010	6	8	9	8	-2	1	3	0
011	9	8	9	8	1	1	0	0
012	8	7	8	7	1	1	0	0
013	5	3	6	3	2	3	1	0
014	9	4	9	8	5	1	0	4
015	8	5	10	10	3	0	2	5
016	5	6	6	7	-1	-1	1	1
017	10	10	10*	10*	0	0	0	0
018	5	7	9	9	-2	0	4	4
019	9	9	9	9	0	0	0	0
021	1	6	8	8	-5	0	7	7
022	10	10	10	10	0	0	0	0
023	8	8	8*	9	0	-1	0	0
024	8	6	5	6*	2	-1	-3	-3
025	8	7	6	7	1	-1	-2	-2
026	9	9	9*	9	0	0	0	0
027	7	8	8	8	-1	0	1	1
028	8**	8	9	9	0	0	1	1
029	8	8	8*	8	0	0	0	0
030	9	7	9*	8	2	1	0	0
031	7	8	7*	8*	-1	-1	0	0
032	0	9	9	9*	-9	0	9	9
034	7	5	7	8	2	-1	0	0

Subject ID	Initial Comfort Score		Final Comfort Score		Difference between initial comfort scores (hand minus hydrostatic cast)	Difference between final comfort scores (hand minus hydrostatic cast)	Difference between initial and final hand casting	Difference between initial and final hydrostatic casting
	Hand Casting	Hydrostatic Casting	Hand Casting	Hydrostatic Casting				
035	6	8	7	8*	-2	-1	1	1
036	8	9	8	9*	-1	-1	0	0
037	9	7	9*	8	2	1	0	1
038	6	10	7	10*	-4	-3	1	0
039	7	5	9	5	2	4	2	0
040	5	7	6	8	-2	-2	1	1
041	7**	9	8**	9*	-2	-1	1	0
042	6	8	6**	8**	-2	-2	0	0
043	9	7	9**	9	2	0	0	2
044	5**	7**	7**	8	-2	-1	2	1
045	7	6	7**	7	1	0	0	1
046	3	4	5	6	-1	-1	2	2
047	4	9	8	9*	-5	-1	4	0
048	6**	7	9	9	-1	0	3	2
049	5**	8	6	8	-3	-2	1	0
050	2	7	0	9*	-5	-9	-2	2
051	2	7	2	8	-5	-6	0	1
052	9**	7	9*	7	2	2	0	0
053	5	8	7**	8	-3	-1	2	0
054	9	7	9	8	2	1	0	1
056	9	9	9	9	0	0	0	0
057	5	6	7	7	-1	0	2	1
058	5	3	7	6	2	1	2	3
059	5	1	3	4	4	-1	-2	3
060	10	10	10	10	0	0	0	0
061	7	6	7	7	1	0	0	1
062	3	3	6	3	0	3	3	0
063	6	7	7	6	-1	1	1	-1
064	9	9	9	9*	0	0	0	0
065	9	9	9	9	0	0	0	0
066	7	7	8	7	0	1	1	0
067	8	10	10	10*	-2	0	2	0
068	8	8	8*	8*	0	0	0	0
069	5	5	7	7	0	0	2	2
070	8	6	10	8	2	2	2	2
071	6	5	8	7	1	1	2	2
072	6	7	6	8	-1	-2	0	1
073	6**	7**	6*	9	-1	-3	0	2
074	9	7	9*	8	2	1	0	1

Subject ID	Initial Comfort Score		Final Comfort Score		Difference between initial comfort scores (hand minus hydrostatic cast)	Difference between final comfort scores (hand minus hydrostatic cast)	Difference between initial and final hand casting	Difference between initial and final hydrostatic casting
	Hand Casting	Hydrostatic Casting	Hand Casting	Hydrostatic Casting				
075	8	7**	8**	8**	1	0	0	1
076	5	5**	6**	7**	0	-1	1	2
077	8	6	8*	6*	2	2	0	0
078	10	7	10*	7*	3	3	0	0
080	8	7	9	8	1	1	1	1

**NM: not modified. Initial SCS score was carried forward.*

***Some participants provided half scores. These scores were rounded down to nearest whole number to ensure parity in the scale across sites.*
