# Supplementary material for: Randomized crossover trial of hand and hydrostatic casting for custom lower limb prosthetic sockets: Assessing socket comfort and fabrication time
Source: PLoS One. 2025 Nov 21;20(11):e0337185. doi: 10.1371/journal.pone.0337185 (PMC12637896; doi:10.1371/journal.pone.0337185)
Supplement: S4 Table — (PDF) [file pone.0337185.s004.pdf]

S4 Table. Fabrication Time and Casting Approach.

Subject ID	Hand Casting				Hydrostatic Casting			
	Total Time (hr:min:sec)	Casting Time (hr:min:sec)	Rectification Time (hr:min:sec)	Check Socket Modification Time (hr:min:sec)	Total Time (hr:min:sec)	Casting Time (hr:min:sec)	Rectification Time (hr:min:sec)	Check Socket Modification Time (hr:min:sec)
001	00:50:06	00:12:18	00:20:52	00:16:56	00:43:20	00:23:02	00:14:24	00:05:54
002	00:45:48	00:05:56	00:14:52	00:25:00	00:53:55	00:25:30	00:12:01	00:16:24
003	00:34:00	00:05:48	00:18:14	00:09:58	00:46:41	00:25:23	00:09:01	00:11:57
004	00:41:34	00:06:54	00:19:23	00:15:17	00:57:10	00:30:26	00:10:34	00:16:10
005	00:32:52	00:05:03	00:14:16	00:13:33	00:43:18	00:23:13	00:08:48	00:11:17
006	00:41:34	00:05:45	00:13:22	00:22:27	00:54:44	00:26:09	00:08:28	00:20:07
007	00:39:42	00:06:03	00:11:22	00:22:17	00:48:49	00:23:53	00:10:39	00:14:17
008	00:42:49	00:07:05	00:13:31	00:22:13	01:01:34	00:26:44	00:06:47	00:28:03
010	00:33:28	00:06:55	00:14:43	00:11:50	00:43:58	00:22:47	00:09:31	00:11:40
011	00:40:00	00:06:53	00:18:50	00:14:17	00:51:19	00:23:57	00:11:10	00:16:12
012	00:34:17	00:09:08	00:09:00	00:16:09	00:56:56	00:37:30	00:04:58	00:14:28
013	00:20:51	00:08:34	00:06:00	00:06:17	00:41:07	00:26:11	00:04:55	00:10:01
014	00:23:24	00:06:45	00:08:55	00:07:44	00:42:44	00:27:09	00:04:50	00:10:45
015	00:21:08	00:06:09	00:08:36	00:06:23	00:39:03	00:19:57	00:06:43	00:12:23
016	00:38:47	00:07:30	00:24:01	00:07:16	00:49:01	00:21:00	00:20:16	00:07:45
017	00:29:06	00:06:01	00:23:05	00:00:00	00:32:31	00:21:16	00:11:15	00:00:00
018	00:37:05	00:06:27	00:27:42	00:02:56	00:49:41	00:27:20	00:10:45	00:11:36
019	00:39:47	00:07:25	00:25:36	00:06:46	00:36:31	00:18:25	00:10:51	00:07:15
021	00:44:33	00:06:14	00:24:40	00:12:39	00:41:55	00:21:55	00:10:46	00:09:14
022	00:32:47	00:08:14	00:24:01	00:00:32	00:43:33	00:25:12	00:13:13	00:05:08
023	00:32:07	00:07:47	00:24;20	00:00:00	00:43:17	00:20:17	00:12:34	00:10:26
024	00:43:14	00:06:40	00:25:50	00:10:44	00:43:19	00:17:45	00:25:34	00:00:00
025	00:35:19	00:07:12	00:21:05	00:07:02	01:06:45	00:34:56	00:25:57	00:05:52
026	00:30:15	00:07:26	00:22:49	00:00:00	00:46:07	00:14:03	00:17:46	00:14:18
027	00:30:25	00:06:39	00:22:50	00:00:56	00:40:17	00:15:29	00:23:29	00:01:19
028	00:34:48	00:06:15	00:23:30	00:05:03	00:44:36	00:18:43	00:18:37	00:07:16
029	00:34:16	00:06:21	00:27:55	00:00:00	00:39:59	00:20:45	00:17:40	00:01:34
030	00:33:01	00:06:51	00:26:10	00:00:00	00:44:49	00:18:05	00:21:51	00:04:53
031	00:29:00	00:06:17	00:22:43	00:00:00	00:36:11	00:17:24	00:18:47	00:00:00
032	00:29:01	00:06:57	00:15:46	00:06:18	00:38:17	00:21:00	00:17:17	00:00:00
034	00:27:44	00:07:14	00:20:00	00:00:30	00:35:26	00:16:19	00:18:28	00:00:39
035	00:29:01	00:07:41	00:16:25	00:04:55	00:36:23	00:25:00	00:11:23	00:00:00
036	00:35:40	00:14:00	00:20:00	00:01:40	00:24:00	00:12:00	00:12:00	00:00:00
037	00:47:00	00:17:00	00:30:00	00:00:00	00:52:05	00:27:40	00:18:00	00:06:25
038	01:06:30	00:18:00	00:32:00	00:16:30	00:47:14	00:24:14	00:22:00	00:00:00
039	01:23:30	00:20:00	00:50:00	00:13:30	00:59:00	00:30:00	00:20:00	00:09:00
040	01:07:50	00:15:00	00:45:00	00:07:50	01:00:50	00:31:30	00:18:00	00:11:20
041	01:26:58	00:15:52	01:07:00	00:03:50	01:05:19	00:26:19	00:39:00	00:00:00

Subject ID	Hand Casting				Hydrostatic Casting			
	Total Time (hr:min:sec)	Casting Time (hr:min:sec)	Rectification Time (hr:min:sec)	Check Socket Modification Time (hr:min:sec)	Total Time (hr:min:sec)	Casting Time (hr:min:sec)	Rectification Time (hr:min:sec)	Check Socket Modification Time (hr:min:sec)
042	01:20:00	00:23:00	00:50:00	00:10:00	01:16:00	00:30:00	00:36:00	00:10:00
043	01:38:52	00:24:13	01:04:50	00:09:49	01:03:52	00:32:20	00:27:30	00:04:02
044	00:58:38	00:22:56	00:31:05	00:04:37	01:14:39	00:37:16	00:30:00	00:07:23
045	00:55:51	00:16:08	00:31:19	00:08:24	01:09:11	00:40:03	00:23:27	00:05:41
046	01:26:08	00:23:38	00:50:00	00:12:30	00:57:43	00:31:55	00:20:00	00:05:48
047	00:59:21	00:17:07	00:25:00	00:17:14	00:42:16	00:22:16	00:20:00	00:00:00
048	01:05:16	00:18:45	00:41:00	00:05:45	00:37:42	00:20:02	00:14:00	00:03:40
049	01:11:30	00:15:00	00:50:00	00:06:30	00:48:00	00:25:00	00:18:00	00:05:00
050	01:02:20	00:14:30	00:33:50	00:14:00	01:01:10	00:29:30	00:22:00	00:08:40
051	01:12:20	00:12:20	00:45:00	00:15:00	00:58:30	00:22:10	00:20:00	00:16:20
052	00:34:20	00:14:20	00:20:00	00:00:00	00:37:30	00:15:30	00:15:00	00:07:00
053	00:52:13	00:11:47	00:35:17	00:05:09	00:44:50	00:17:29	00:19:36	00:07:45
054	01:01:19	00:17:13	00:43:00	00:01:06	00:49:36	00:24:27	00:21:18	00:03:31
056	00:34:21	00:08:17	00:16:58	00:09:06	00:51:56	00:22:31	00:19:00	00:10:25
057	00:30:23	00:07:30	00:13:54	00:08:59	00:57;33	00:36:10	00:09:09	00:12:14
058	00:38:10	00:07:16	00:13:07	00:17:47	00:55:13	00:23:45	00:11:15	00:20:13
059	01:09:15	00:08:24	00:18:29	00:42:22	01:04:29	00:34:49	00:13:08	00:16:32
060	00:18:20	00:06:02	00:08:38	00:03:40	00:45:38	00:26:21	00:07:05	00:12:12
061	00:31:14	00:06:16	00:11:39	00:13:19	01:01:07	00:25:59	00:06:46	00:28:22
062	00:27:06	00:07:16	00:08:31	00:11:19	00:40:18	00:26:41	00:05:27	00:08:10
063	00:22:01	00:05:50	00:10:04	00:06:07	00:42:12	00:26:14	00:07:33	00:08:25
064	00:31:50	00:07:00	00:15:50	00:09:00	00:32:40	00:19:15	00:13:25	00:00:00
065	00:33:42	00:05:13	00:21:20	00:07:09	00:53:59	00:29:17	00:24:12	00:00:30
066	00:33:42	00:06:57	00:17:58	00:08:47	00:56:04	00:23:27	00:15:40	00:16:57
067	00:31:30	00:04:11	00:21:55	00:05:24	00:33:47	00:20:04	00:13:43	00:00:00
068	00:24:05	00:05:05	00:19:00	00:00:00	00:36:28	00:22:28	00:14:00	00:00:00
069	00:32:29	00:05:25	00:16:04	00:11:00	00:40:34	00:15:01	00:14:49	00:10:44
070	00:52:00	00:22:00	00:28:00	00:02:00	01:05:00	00:25:00	00:29:00	00:11:00
071	00:40:00	00:13:00	00:23:00	00:04:00	00:47:05	00:19:35	00:18:00	00:09:30
072	00:46:50	00:13.30	00:32:00	00:01:20	00:31:00	00:12:10	00:17:00	00:01:50
073	00:44:20	00:14:20	00:30:00	00:00:00	00:38:50	00:15:00	00:21:00	00:02:50
074	00:35:41	00:12:56	00:22:45	00:00:00	00:38:45	00:17:30	00:19:27	00:01:48
075	00:37:33	00:09:02	00:26:26	00:02:05	00:39:41	00:16:10	00:21:12	00:02:19
076	01:02:37	00:18:07	00:36:30	00:08:00	00:46:17	00:20:00	00:22:30	00:03:47
077	00:47:00	00:22:00	00:25:00	00:00:00	00:48:15	00:23:15	00:25:00	00:00:00
078	00:41:30	00:14:30	00:27:00	00:00:00	00:41:30	00:16:30	00:25:00	00:00:00
080	00:51:10	00:07:50	00:39:00	00:04:20	00:51:11	00:21:51	00:27:00	00:02:20
