# Supplementary material for: Randomized crossover trial of hand and hydrostatic casting for custom lower limb prosthetic sockets: Assessing socket comfort and fabrication time
Source: PLoS One. 2025 Nov 21;20(11):e0337185. doi: 10.1371/journal.pone.0337185 (PMC12637896; doi:10.1371/journal.pone.0337185)
Supplement: S1 File — (PDF) [file pone.0337185.s005.pdf]

S1 File. Sample size calculation and randomization sequence.

Sample size calculation

Using unpublished pilot data wherein persons with transtibial amputation used the Socket Comfort Score (SCS) [1] to assess sockets fabricated using both casting approaches, we ran a range of power and sample size calculations using STATA MP 15 software (StataCorp. LLC, College Station, TX). Based on our pilot data, means were 8.4 and 6.3 for sockets made with each casting technique, with standard deviations of 1.3 and 2.8, respectively. The correlation between the two socket methods was 0.4. Keeping 0.8 power and 0.05 alpha constant, we looked at the total sample size that would be required to detect a difference of 3-, 2- and 1-points in SCS. Assuming a dependent 2-sided t-test framework, the total N to detect a 1-point difference was 54, to detect a 2-point difference was 16, and to detect a 3-point difference was 8. Recent assessment of the test-retest reliability of the SCS over 2-3 days suggests that the minimal detectable change is 2.73 [2]. However, since our testing takes place within a single study visit, we wanted to have sufficient power to detect a 1-point difference in SCS. We thought that this was the minimal difference that would be meaningful for our testing scenario and, when put in terms of standard deviation units of change, it represented a small to medium effect size. We expected dropouts to be modest given the minimal time and burden of the study, but to err on the side of caution we proposed to enroll 20 transtibial participants at each site for a total of N=60. This number allows for 10% attrition.

We did not have similar pilot data upon which to base a power calculation for transfemoral participants. Hence, we used the above power calculations in combination with

knowledge of the lower proportion of persons with transfemoral amputation to determine a sample size [3]. A total sample size of 16 transfemoral participants is needed to detect a two-point change in SCS, which equates to six participants per site (rounding up slightly). However, we decided to recruit 10 transfemoral participants per site for a total of N=30. Therefore, allowing for 10% attrition, this number was 36% more than the available data suggests is needed.

Calculation of randomization sequence

We utilized PROC PLAN in SAS 9.4 (SAS Institute Inc., Cary, NC) to randomize at visit 2: the casting order (hand-A, hydrostatic casting-B) and at visit 3: socket fitting order (hand-A, hydrostatic casting-B). For each of the 3 sites, we randomized order sequence (AB, BA) with 2 blocks of length 10 for the N=20 transtibial participants. Then we did the same for the N=10 transfemoral participants at each site with 2 blocks of length 6. We did not use the 6th and 12th rows due to N=10.

References

1. Hanspal RS, Fisher K, Nieveen R. Prosthetic socket fit comfort score. Disabil Rehabil. 2003;25(22):1278-80.
2. Hafner BJ, Morgan SJ, Askew RL, Salem R. Psychometric evaluation of self-report outcome measures for prosthetic applications. J Rehabil Res Dev. 2016;53(6):797-812.
3. Adams P, Hendershot G, Marano M. Current estimates from the National Health Interview Survey, 1996. Vital Health Stat. 1999;10(200).
