# Supplementary material for: Randomized crossover trial of hand and hydrostatic casting for custom lower limb prosthetic sockets: Assessing socket comfort and fabrication time
Source: PLoS One. 2025 Nov 21;20(11):e0337185. doi: 10.1371/journal.pone.0337185 (PMC12637896; doi:10.1371/journal.pone.0337185)
Supplement: S2 File — (PDF) [file pone.0337185.s006.pdf]

S1 File. Socket Fit Evaluation Checklist.

The socket-fit-checklist was used by all prosthetists to assess the fit of the diagnostic socket at visit 3, after initial comfort score was recorded. The socket-fit-checklist was developed prior to commencement of data collection by consensus of the study team, including the prosthetists who participated in the study. It reflected the typical diagnostic socket features that a prosthetist would assess clinically and placed them in an ordered sequence.

Instructions

- The following checklist is intended to be used AFTER the initial socket comfort score has been assessed. That means that the socket and liner will have been donned.
- To begin the socket fit evaluation process, please remove the socket and liner from the subject.

Socket Fit Evaluation Checklist

Subject ID:	Date:	Socket ID:	Re-pull: 0 1 2

When subject removes ***their usual prosthesis***, record how many socks (and what ply) or half liners (and what thickness) they should be wearing to achieve a good fit.	Socks/Half liner Today:
How does this sock number/ply or half liners compare to how many sock number/ply or half liners the subject was wearing with ***their usual prosthesis*** on the day of casting (visit 2)?	Socks/Half liner at Casting:
Calculate number/ply of socks or half liners for check socket goal fit today.	Goal fit:

Answer these items for all sockets.

		Yes	No	If no, what did you do to address the issue?
Before Donning	Check socket shape; does it look as you expect?			
	Is the liner appropriately sized?			
During Donning	Donning may occur seated or standing depending on the usual practice of the prosthetist for a particular socket design. It is ok if subject has to "pump" a couple of times during donning if that is typically necessary.			
	Is the resistance during donning appropriate, without distal end discomfort or pain?			

*Anatomical landmarks

For items marked with an asterisk*, consider the following anatomical landmarks as appropriate:

TT	TF
Crest of tibia and fibula<br>Head of fibula<br>Posterior area of the tibial medial condyle<br>Inferior border of patella<br>Insertion of hamstring tendons	Ramus<br>Ischium<br>Adductor tendon<br>Distal end of femur<br>Apex of distal end soft tissue

**Total contact on distal end

For items marked with two asterisks**, consider the following tests:

TT	TF
Remove valve, if used, and check visually.<br>Slight compression of the prosthetist's 5th finger when inserted in the valve hole and compressed by the subject.	

***Moving around with the socket

For item marked with three asterisks***, consider the following tests:

TT	TF
If subject has valgus knee, check pressure on medial tibia; if subject has varus knee, check pressure on lateral tibia and fibula	Check for discomfort on posterior/lateral end of femur, restrictions in hip extension, contact of posterior/lateral socket when hip is flexed

Answer these items for **TT SOCKET** only. Otherwise go to next page.

		Yes	No	If no, what did you do to address the issue?
Partial (double limb) weight bearing	Ask subject to stand in the parallel bars and place equal weight on both limbs. Adjust the height of the stander with a relatively firm surface) so that hips are level when the subject is weight bearing equally through both legs.			
	Is the subject comfortable?			
	Ask subject to gradually increase weight on the prosthetic socket (may use repetitive weight shifting).			
Standing	No excessive pressure encountered* as subject increases weight on the socket?			
	**Have you obtained total contact of the residual limb with the distal end of the socket?			
	Do anatomical landmarks* fit into the appropriate locations in the socket?			
	The mediolateral dimension at the femoral condyles is <u>not</u> loose and there is no gapping of the proximal lateral socket that would allow a varus moment?			
Full (single limb) weight bearing	Are all pressure intolerant areas* comfortable?			
	Have you achieved a total contact fit, such that the subject cannot discern any localized pressures?			

Answer these items for **TF IC SOCKET** only. Otherwise go to next page.

Phase	Item	Yes	No	If no, what did you do to address the issue?
Partial (double limb) weight bearing	Ask subject to stand in the parallel bars and place equal weight on both limbs. Adjust the height of the stander so that hips are level when the subject is weight bearing equally through both legs.			
	Is the subject comfortable?			
	Is the adductor longus tendon positioned in the medial anterior aspect of the socket and free from excessive pressure?			
	Does the anterior-medial wall allow the pubic ramus to exit out of the socket without pressure or pain?			
Standing	Ask subject to gradually increase weight on the prosthetic socket (may use repetitive weight shifting).			
	No gapping or excessive pressure* at trim lines?			
	No excessive pressure encountered* as subject increases weight on the socket?			
	**Have you obtained total contact of the residual limb with the distal end of the socket?			
Full (single limb) weight bearing	Is the socket tight on the residual limb when you pull laterally while resisting lateral motion of the prosthetic side ilium? (if not applicable, write n/a)			
	Is the greater trochanter comfortable?			

Answer these items for <u>**TF SI SOCKET**</u> only. Otherwise go to next page.

Phase	Item	Yes	No	If no, what did you do to address the issue?
Partial (double limb) weight bearing	Ask subject to stand in the parallel bars and place equal weight on both limbs. Adjust the height of the stander so that hips are level when the subject is weight bearing equally through both legs.			
	Is the subject comfortable?			
	Ask subject to gradually increase weight on the prosthetic socket (may use repetitive weight shifting).			
Standing	No excessive pressure encountered* as subject increases weight on the socket?			
	**Have you obtained total contact of the residual limb with the distal end of the socket?			
	Proximal medial trimline <u>does not</u> impinge on the ischial tuberosity, pubic ramus or adductor tendon?			
	Is all medial tissue comfortable?			
	Is the socket free of any gapping along the lateral brim or lateral wall?			
Full (single limb) weight bearing	Is the lateral posterior brim snug to the residual limb?			
	Have you achieved a total contact fit?			

Answer these items for all sockets.

Phase	Item	Yes	No	If no, what did you do to address the issue?
Weight bearing on unstable surface	Place a foam block on the stander under the socket and ask the subject to move into different knee positions (extension, flexion, varus, valgus) or hip positions (extension, flexion, abduction, adduction).			
	***Can the subject move around with the socket comfortably?			
Standing on intact limb	Ask subject to stand in the parallel bars and hold on with hands while unweighting the residual limb. If appropriate, don a sealing sleeve to test this item.			
	The socket <u>does not</u> move when you try to pull it off?			
	Is the subject comfortable during pulling of the socket (i.e., no pain, uncomfortable tightness or biting of the skin)?			
Non weight bearing, Sitting	Ask subject to sit.			
	Is subject comfortable in sitting?			
	TF: Is hip range of motion available for comfortable forward flexion?			
	TT: Can the knee flex comfortably in sitting with and without the knee sleeve?			
After Doffing	Ask subject to remove socket and liner.			
	The skin <u>does not</u> show any concerning marks or redness?			

If you were **able** to answer "Yes" (with or without modifications) to all the above items relevant to the particular socket design…

Did you achieve your goal fit in terms of socks/half liners?	Yes	No	If no, how many socks/half liners did you end up with:	

If you were **unable** to answer "Yes" (with or without modifications) to all the above items relevant to the particular socket design…

Do you need to re-pull the check socket to address remaining fitting issues?	Yes	No	If no, is the only option to re-cast? Please describe why:	

Do you have any comments about your final socket fit?	
Does the subject have any comments about your final socket fit?	
