# Supplementary material for: Randomized crossover trial of hand and hydrostatic casting for custom lower limb prosthetic sockets: Assessing socket comfort and fabrication time
Source: PLoS One. 2025 Nov 21;20(11):e0337185. doi: 10.1371/journal.pone.0337185 (PMC12637896; doi:10.1371/journal.pone.0337185)
Supplement: S1 Protocol — (PDF) [file pone.0337185.s007.pdf]

MASTER PROTOCOL TITLE:
Comparative Effectiveness of Socket Casting Methods: Improving Form and Fit

VERSION DATE: 07/14/19

MULTI-SITE RESEARCH

This is a prospective, multi-site, assessor-blinded, randomized crossover comparative effectiveness clinical trial to assess two casting techniques. The Northwestern University IRB complies with NIH Policy on the Use of a Single Institutional Review Board for Multi-Site Research; however exceptions to the policy include VA and international sites. Hence, a single IRB will not be used for this project. Instead, this master protocol and a master consent form have been developed for use by all collaborating sites for submission to their respective IRBs with local protocol addenda as necessary. While a single overall protocol will govern this study, each site will be overseen by their own IRB and will submit approvals, amendments, adverse events and protocol deviations to their respective IRBs as needed. Prior to commencement of any work with human subjects, we will apply for IRB approval at each site and then submit for approval from the DOD Human Research Protection Office (HRPO). No work with human subjects will begin until all IRB approvals are received.

Lead Site	Site 1	Site 2
Northwestern University (NU)	Minneapolis Veterans Administration Health Care System (MVAHCS)	INAIL: Istituto Nazionale per L'Assicurazione contro gli Infortuni sul Lavoro
680 N Lake Shore Drive Suite 1100, Chicago IL 60611	1 Veterans Drive, Mailstop 151, Minneapolis, MN 55417	Via Rabuina 14, Budrio Italy
PI: Stefania Fatone, PhD	Site PI: Andrew Hansen, PhD	Site PI: Andrea G. Cutti, PhD

CLINICAL TRIAL REGISTRATION

This trial will be registered on clinicaltrials.gov prior to enrollment of the first subject.

STUDY SUMMARY:

Investigational Agent(s) (Drugs or Devices)	None
IND / IDE / HDE #	Not applicable
Indicate Special Population(s)	☐ Children<br>☐ Children who are wards of the state<br>☐ Adults Unable to Consent<br>☐ Cognitively Impaired Adults<br>☐ Neonates of Uncertain Viability<br>☐ Pregnant Women<br>☐ Prisoners (or other detained/paroled individuals)<br>☐ Students/Employees
Sample Size	90
Funding Source	Department of Defense
Indicate the type of consent to be obtained	☒ Written<br>☐ Verbal/Waiver of Documentation of Informed Consent<br>☐ Waiver of HIPAA Authorization<br>☐ Waiver/Alteration of Consent Process

Research Related Radiation Exposure	☐ Yes<br>☒ No
DSMB / DMC / IDMC	☐ Yes<br>☒ No

OBJECTIVES:

The overall objective of this project is to compare hand casting to standing hydrostatic pressure casting with a water cylinder; including consistency and efficiency of residual limb shape capture, socket fit, and comfort in persons with lower limb amputation. Our overall hypothesis is that standing hydrostatic pressure casting with a water cylinder will lead to more consistent and efficient residual limb shape capture and improved initial socket fit and comfort compared to hand casting. To assess this overall hypothesis, our specific aims are as follows:

Aim 1: To compare the ***consistency*** of two residual limb shape capture techniques in persons with transtibial and transfemoral amputation.

Hypothesis 1.1: Standing hydrostatic pressure casting with a water cylinder results in more consistent cast shape and volume compared to hand casting.

Aim 2: To compare the ***efficiency*** of two residual limb shape capture techniques in persons with transtibial and transfemoral amputation.

Hypothesis 2.1: Standing hydrostatic pressure casting with a water cylinder reduces the time required to cast, rectify and successfully fit an initial diagnostic check socket compared to traditional hand casting.

Hypothesis 2.2: Standing hydrostatic pressure casting using a water cylinder reduces the number of diagnostic check sockets required to achieve a clinically acceptable initial fit compared to traditional hand casting.

Aim 3: To compare the ***outcomes*** of two residual limb shape capture techniques in persons with transtibial and transfemoral amputation.

Hypothesis 3.1: Standing hydrostatic pressure casting with a water cylinder improves socket comfort as compared to hand casting.

Hypothesis 3.2: Standing hydrostatic pressure casting with a water cylinder improves socket fit assessed clinically as compared to hand casting.

Hypothesis 3.3: Sockets made using standing hydrostatic pressure casting with a water cylinder will be preferred more often than those made by hand casting.

BACKGROUND

Rationale for Research/Clinical Trial

One of the most important components of restoring function in persons with lower limb amputation is the precise fitting of the prosthetic socket to the residual limb.[1] A prosthetic socket fits snugly around the residual limb, serving to transfer loads between the body and prosthesis without causing discomfort or pain. Pain is extremely common in people with lower limb amputation. In particular, residual limb pain occurs in 61-76% of people with lower limb amputation and is often related to issues such as prosthetic socket pressure and skin abrasions, among other factors.[2] It has been reported that residual limb pain leads to poorer acceptance of the prosthesis and more prosthesis-related restrictions than people without pain.[3]

Achieving a comfortably fitting socket that does not contribute to residual limb pain is challenging because the residual limb is dynamic in shape and volume. In addition to these physiological challenges, quality of socket fit is influenced by prosthetic fabrication processes, including residual limb shape capture, positive model rectification and prosthesis alignment,[4, 5] as well as interface material selection. There is no consensus on how to achieve and quantitatively confirm good socket fit,[6-8] hence quality of fit remains a subjective assessment.[9] Furthermore, successful socket fittings are largely dependent on the skill, knowledge, and experience of the

prosthetist. As a result, socket refits are common and expensive,[10] and frustrating and burdensome to the prosthesis user.[8]

Relevant Literature

The process of socket fabrication typically consists of residual limb shape capture, positive mold rectification, initial diagnostic socket fitting, and definitive prosthesis delivery (Figure 1). Accurately capturing residual limb shape is a foundational step in the fabrication of a prosthetic socket.[11] The most prevalent shape capture method involves taking a negative wrap cast in a non-weight bearing position and manually manipulating the plaster or fiberglass bandages to conform to the residual limb shape. However, it is challenging to accurately capture the contours of the bony anatomy and to distribute pressure evenly around the residual limb; hence casts are seldom reproducible and consistent,[12] nor do they capture a shape that is ideal for pressure distribution during weight bearing[13]. To improve consistency of shape capture, attempts have been made to develop socket fabrication techniques that rely less on manual manipulation of the negative cast wrap by the prosthetist, such as pressure casting.[12, 14] Pressure casting produces a cast through the application of air,[4, 15-17] vacuum,[11] sand,[18] or water[6, 10, 12, 14, 19-21] pressure to the plaster wrapped residual limb. Some pressure casting systems are designed to be used in sitting and some in standing. Suggested advantages of the pressure casting technique include ease of implementation, elimination of the need for or reduction of the extent of positive model rectification, and improved prosthesis delivery time.[12, 22, 23] In particular, casting with weight bearing pressure in standing is said to allow the residual limb to *"let nature dictate the most realistic and achievable pressure distribution."*[24]

Geil[1] stated that it is crucial that techniques used to produce sockets be accurate, repeatable, cost-effective, and have high patient utility. Convery et al.[5] suggested that the effect of inconsistencies in casting and rectification may be cumulative and Courtney et al.[6] illustrated that there may be an interaction between socket design and alignment in terms of its effect on interface pressures. Hence, to assess the contribution of casting technique to socket fit and comfort, we have to assess outcomes at the initial diagnostic socket fitting stage, before other variables such as dynamic alignment, suspension mechanism, and choice of componentry and interface materials influence these outcomes.

While standing hydrostatic pressure casting using a cylinder of water has been described in the literature, assessment of the consistency of shape capture, efficiency of fabrication and socket fit outcomes using standing hydrostatic pressure casting have been limited to case studies,[6, 14, 20, 22, 24, 25] applications in developing countries,[10, 21] and comparison to the patella tendon bearing socket,[12, 21, 22] which makes up only 8.5% of transtibial lower limb sockets provided in clinical practice in the United States (US) (compared to ~20% each for total surface bearing and hydrostatic sockets).[26] In a systematic review aimed at assessing socket fit across different transtibial socket designs, Safari and Meier[27, 28] concluded that while there is some evidence that

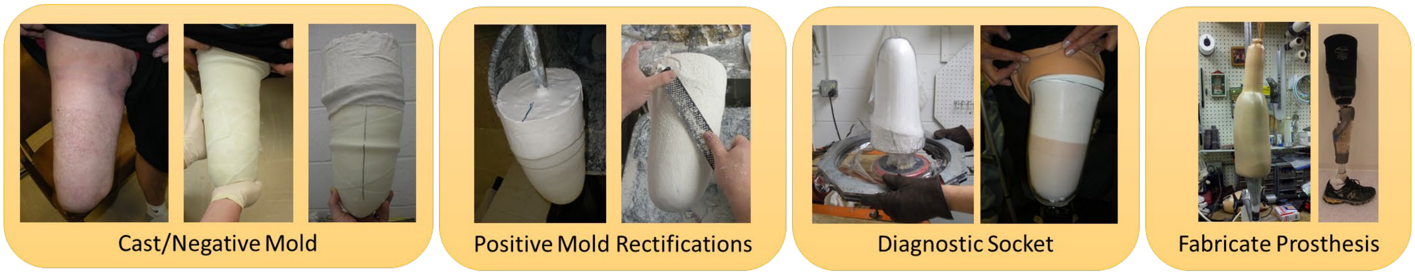

Figure 1 Conventional process of prosthetic socket fabrication beginning with a hand cast (i.e., negative mold) of the residual limb, filling the negative mold with liquid plaster to make a positive mold that can be rectified (i.e., manually sculpted), fabricating a diagnostic socket to check that total contact fit is achieved, and then proceeding to definitive prosthesis fabrication and delivery.

total surface bearing sockets lead to greater activity levels and satisfaction, evidence for hydrostatic sockets is inadequate and in need of further research.

While further research is still needed, standing hydrostatic pressure casting is gaining use in clinical practice, including within the Veterans Administration (VA) and Department of Defense (DOD), as an alternative to hand casting now that a standing hydrostatic pressure casting system is commercially available in the US (Symphonie Aqua System™, Romedis gmbh, Germany) (Figure 2). This system allows up to 100% body weight to be applied to the residual limb during casting, which may contribute to better shape capture and improved socket fittings by creating a shape that more evenly distributes weight-bearing loads. Modelling of the residual limb-water interface pressures for different loading conditions suggests that there is a positive correlation wherein an increase in load increases the interface pressure.[13] It has been argued that the most effective socket relies on a hydrostatic principle wherein controlled pressure is used to deform tissue under load such that hypothetically the stiffest path principle is achieved and internal shear stresses minimized.[25, 29]

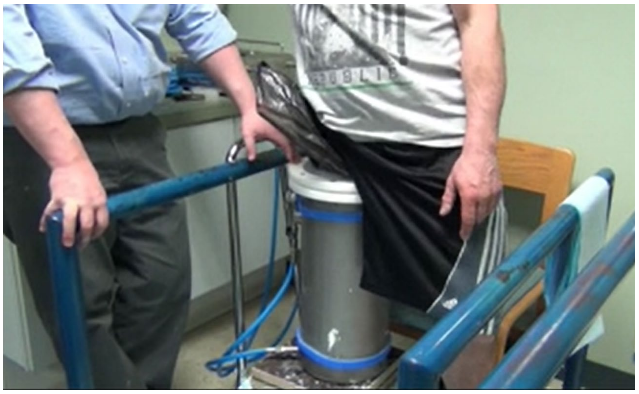

Figure 2 Using standing hydrostatic pressure casting with a water cylinder (Symphonie Aqua System™ Romedis gmbh, Germany) to cast a transfemoral subject. Plaster bandage is wrapped around the residual limb the same way as for hand casting. The limb is then placed in a cylinder that is filled with water. Bladder bulges proximally as pressure increases inside cylinder and body weight is applied.

Preliminary Data

Given the use of physics, specifically Pascal's principal,[13] to shape the residual limb rather than the prosthetist's hands, it has been proposed that standing hydrostatic pressure casting using a water cylinder results in better fitting sockets than hand casting. Co-Investigator Caldwell's preliminary data (n=7) comparing transtibial sockets made using standing hydrostatic pressure casting with a water cylinder and hand casting suggest that socket comfort at initial fitting is significantly better for the sockets made from standing hydrostatic pressure casts (mean Socket Comfort Scores for pressure versus hand casting: 8.4±1.3 vs 6.3±2.8, p=0.04) (Figure 3). Subjective comments about the sockets also suggested that patients perceived a better fit with the sockets made from standing hydrostatic pressure casts (Table 1). This preliminary data suggests that persons with transtibial amputation can perceive a distinct difference in socket fit and comfort between sockets fabricated using these two casting techniques.

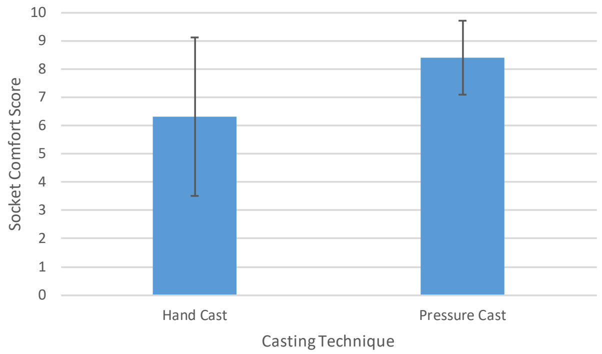

Figure 3 Socket Comfort Score for transtibial check sockets made from two casting techniques.

Table 1 Subjective comments about sockets made using two casting techniques.

Symphonie Aqua System™	Hand Cast
• "Feels like I could have more endurance with this socket."<br>• "Wow! A lot better, no pressure on the bottom, tighter fit around the knee. First time I put my stump in a socket and it was good to go."<br>• "I don't feel anything against the bone in the front, a little better than the other [socket]. Contoured more to me, snugger all over. Haven't felt something so solid around me in a long time; instead of flopping around inside, it felt like it was holding me all around."<br>• "No binding. Feels like a good fit; feels great. Really, really good. Feels even."<br>• "Both feel very close. Other [socket] is slightly more comfortable; this one has a little more pressure on the bottom in the back of the leg."<br>• "Better. Good tight fit around the knee. Not hitting the bottom. More stability on top."	• "Feels like a ring of pressure around the distal end which extends proximally as more pressure is applied."<br>• "Hitting the bottom."<br>• "In a little deeper; pretty close to the [other socket]."<br>• "Feels pretty good. Don't get the air pockets inside like usual."<br>• "Feels like tightness at the lower end, more pressure on the bottom. Tighter than the other [socket]. Feels a little more squeezed."<br>• "Pressure in the back goes away in this [socket]. Definitely more comfortable. Feel a 50:50 weight distribution in this [socket]."<br>• "Definitely hitting the bottom. Loose. Not very comfortable."

Use of standing hydrostatic pressure casting by Co-Investigator Cutti in a series of 64 transfemoral socket clinical fittings in relatively young traumatic amputees (mean age of 46±13 years), suggested that the Symphonie Aqua System™ is feasible for use in clinical practice with very little recasting (only 5 out of 64 sockets needed recasting) and minimal rectifications required compared to hand casting.[30] Hence, standing hydrostatic pressure casting potentially offers a standardized procedure with which to produce comfortably fitting sockets with repeatable results that are less dependent on a prosthetist's casting and rectification skills and more efficient of the prosthetist and prosthesis user's time.

While standing hydrostatic pressure casting can help fabricate transtibial sockets relatively easily, it is less straightforward for transfemoral sockets such as the ischial containment socket where the proximal trim line must contain pelvic anatomy. The pelvic anatomy is difficult to capture effectively using a water cylinder. However, the system works quite well for sub-ischial sockets given their simpler cylindrical shape only contains the thigh.[25, 31-36] With previous funding from the DOD/Congressionally Directed Medical Research Program (CDMRP) Peer Reviewed Orthopedic Program (#W81XWH-10-1-0744), Principal Investigator (PI) Fatone and Co-Investigator Caldwell developed the Northwestern University Flexible Sub-Ischial Vacuum (NU-FlexSIV) Socket for persons with transfemoral amputation.[33-35] There has been growing clinical implementation both nationally and internationally since Dr. Fatone and Mr. Caldwell began teaching the NU-FlexSIV Socket technique to prosthetists in 2015.[37-42] Hence, the NU-FlexSIV Socket, and accompanying Northwestern University Flexible Sub-Ischial Suction (NU-FlexSIS) Socket,[36] are viable designs for use with the Symphonie Aqua System™.

Additionally, as part of the development process, the rectification procedure required to fabricate the NU-FlexSIV Socket was quantified using rectification maps.[35] This involved digitizing negative molds of unrectified and rectified positive models, aligning the two shapes using an Iterative Closest Point algorithm, and calculating the difference in depth between corresponding points on pairs of casts to assess shape change due to rectification[35] (Figure 4). Digitizing rectifications in this way led to the development of a Computer Aided Design-Computer Aided

Manufacturing (CAD-CAM) template for the NU-FlexSIV Socket.[35] More recently, the same technique was used to assess socket volumes as a surrogate for change in residual limb volume from nine NU-FlexSIV Sockets provided to an individual with transfemoral amputation over the past eleven years. Scanning of the sockets showed that socket volume increased 31%, from a low of 2659.2 cm^3 in 2006 to a high of 3490.6 cm^3 in 2016, while body weight remained stable. Similar scanning and digitizing techniques can be used to assess shape and volume differences between casting techniques for the proposed study.

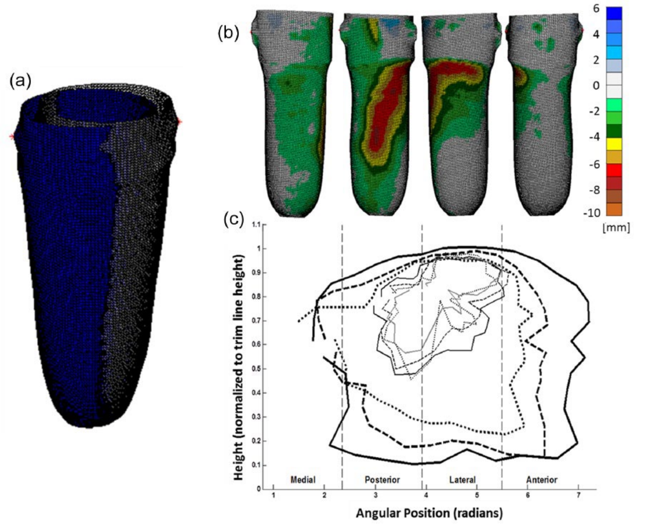

Figure 4 Comparison of casts before and after alignment: (a) juxtaposition of unrectified and rectified casts before alignment. (b) From left to right: medial, posterior, lateral, and anterior views of rectification map from a single representative subject. Color-coded scale indicates the difference in position (mm) of corresponding points between unrectified and rectified casts. (c) Average template depicted as the average contours from 30 casts plotted on the θ–*Z* plane. (Taken from Fatone et al.[35])

It may be perceived that standing hydrostatic pressure casting can only be used on uncomplicated residual limbs. However, our experience indicates that it works well for residual limbs that may be considered complicated due to their short length, odd shape, thin tissue coverage, and/or presence of scars, invaginations, bone spurs and heterotopic ossification. For example, the transtibial residual limbs shown in Figure 5 were fit successfully in clinical practice by Co-Investigator Ryan Caldwell using casts made with the Symphonie Aqua System™. It has been our experience that casts made with standing hydrostatic pressure casting are able to distribute pressure in a way that accounts for issues such as heterotropic ossification and bony prominences without additional modifications. Neuromas are not as easily addressed as the standing hydrostatic pressure casting procedure may apply pressure on the painful/sensitive area if it is superficial. At INAIL, the prototype Symphonie Aqua System was assessed for feasibility of clinical use exclusively in persons with traumatic transfemoral amputation. Given these preliminary experiences with standing hydrostatic

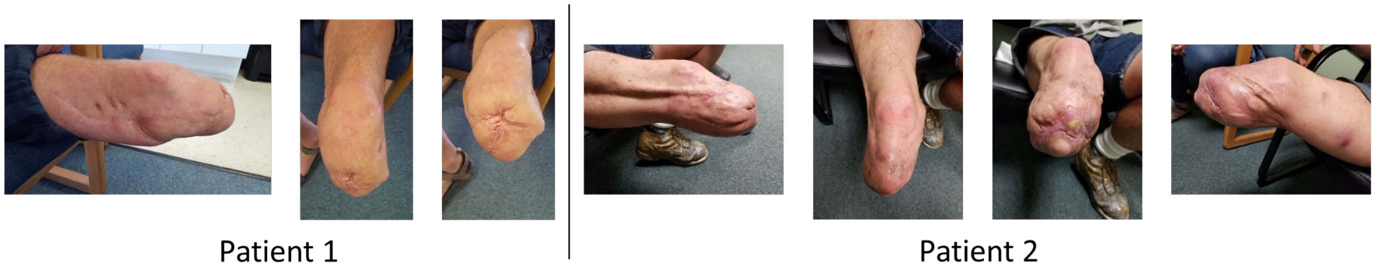

Patient 1

Patient 2

Figure 5 Example of complicated transtibial residual limbs fit with sockets made using the Symphonie Aqua System™. Patient 1 has been an amputee for decades and after wearing this socket for two years reports that it is the best socket he has ever received. During that time, his socket has needed minimal adjustments and the patient has maintained a very active lifestyle.

pressure casting we are confident that a broad range of Service Members and Veterans with amputation can be fit with sockets fabricated using this approach.

STUDY DESIGN

Figure 6 provides an overview of the proposed study design. This is a multi-site, assessor-blinded, randomized crossover comparative effectiveness clinical trial to assess initial socket fit achieved with two casting techniques: hand casting and standing hydrostatic pressure casting with a water cylinder. Subjects with transtibial and transfemoral amputations enrolled in the study can proceed through the clinical process of socket fabrication such that each step addresses a different aim of the study. We believe that comparison of the fabrication procedure up to the point of initial check socket fit will provide an assessment of the contribution that the casting approach makes to socket fit and comfort without the confounding influence of choice of componentry, suspension mechanism, and alignment. While the military and VA have excellent prosthetists who can work iteratively to make great sockets, this process can be frustrating and burdensome to the prosthesis user. Hence, this trial will show whether standing, hydrostatic pressure casting using a water cylinder is a repeatable and efficient alternative to conventional hand casting for prosthetic socket fabrication that results in a more comfortable socket "right out the gate." This trial will provide the data necessary to support decisions about clinical implementation of standing hydrostatic pressure casting systems in the VA and Military Treatment Facilities (MTFs).

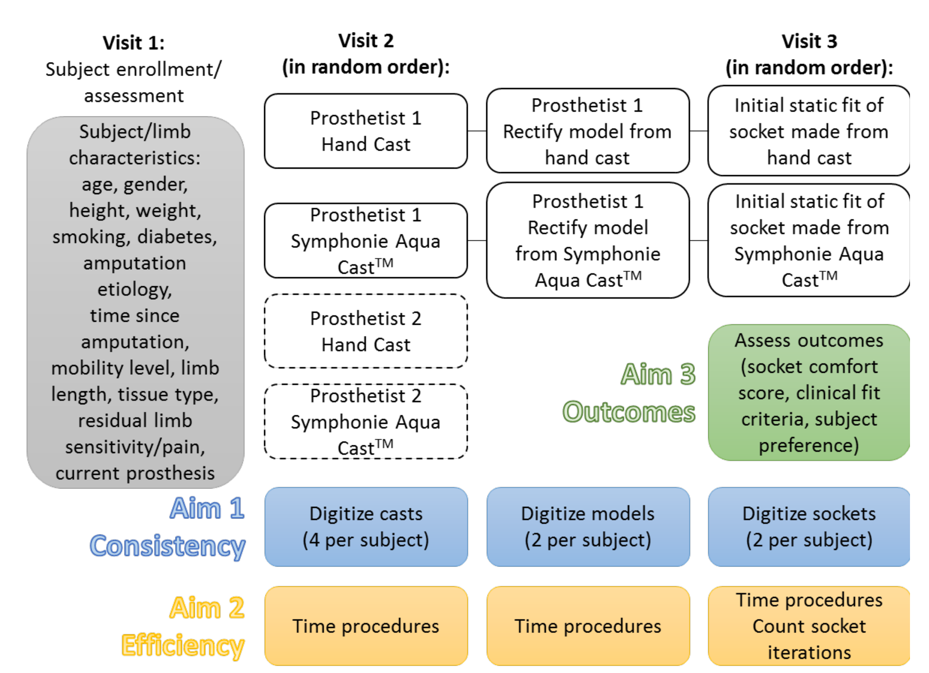

Figure 6 Overview of the proposed experimental approach with endpoints to be measured for each aim.

STUDY ENDPOINTS

Primary and Secondary Outcomes

Consistent with the preliminary data used to power this study, the primary outcome of this trial is socket comfort at the first socket fitting (before and after the prosthetist has completed any modifications necessary to attempt to achieve a satisfactory fit). See Appendix A for more information regarding the Socket Comfort Score. We believe this is the most important patient-centered outcome related to improved residual limb shape capture processes. Additionally, recent work by Sanders et al.[7] indicated that Socket Comfort Score[43] is a viable metric for detection of differences in socket fit among persons with transtibial amputation and our own previous work showed the same sensitivity in persons with transfemoral amputation.[44] If sockets are comfortable, prosthesis users are more likely to wear them and have the potential to be more functional. Other secondary outcomes are important for documenting process outcomes (comparing the shape capture processes directly) that help improve understanding of how the primary outcome is achieved. Therefore, for Aim 1, we will digitize casts and models to calculate

volume and shape changes. As was done by Dr. Fatone when describing a new sub-ischial socket technique,[35, 45] rectification maps will be used to assess cast shape and volume change. For Aim 2, we will assess the fabrication time and number of check socket fittings required to achieve a satisfactory initial socket fit. For Aim 3, we will assess socket comfort, fit and preference of the prosthesis user resulting from the two socket fabrication processes.

STUDY INTERVENTION(S) / INVESTIGATIONAL AGENT(S):

The interventions to be tested in this clinical trial are methods of residual limb shape capture (hand casting vs standing pressure casting using a water cylinder). As defined by clinicaltrials.gov an intervention is a process or action that is the focus of a clinical study and may include noninvasive approaches, such as those used in the fabrication of a custom-made prosthetic socket.

Hand casting of the residual limb using plaster or fiberglass bandages is the current-standard of care, while a standing pressure casting system using a water cylinder has become commercially available only recently. According to the manufacturer, the Symphonie Aqua System™ (Romedis gmbh, Germany) is considered workshop equipment intended solely for the fabrication of a plaster cast of the lower limb after amputation. Important components of the system are illustrated in Figure 7. The system should be used in a location where the person with amputation has some support to hold onto during standing. In preparation for casting, the cylinder is filled with water, which is then transferred to the compensation reservoir while the silicone membrane is coated with plaster insulation cream. Water is then allowed to return to the cylinder. Plaster bandages are wrapped without tension around the residual limb while the person with amputation is seated. Then they are asked to stand and insert their residual limb into the silicone membrane of the pressure vessel. As they do this, the stopcock is opened and a corresponding volume of water is allowed to escape the pressure vessel. Close the stopcock once the desired amount of the residual limb is encased by the pressure cylinder. At this point, the person with amputation should be able to place full weight onto the pressure cylinder supported by the water. Once the plaster has fully hardened, open the stopcock again and remove the residual limb from the pressure vessel.

There are few potential risks to the person with amputation from being cast in this system: the main one is that they might fall while maneuvering their residual limb into and out of the pressure cylinder. This risk is minimized by providing external support in the form of parallel bars or a walking frame. The main benefit of using this system is in achieving a better fitting and more comfortable prosthetic socket “right out the gate”.

Standing pressure casting using a cylinder of water has been described in the literature, but assessment of the consistency of shape capture, efficiency

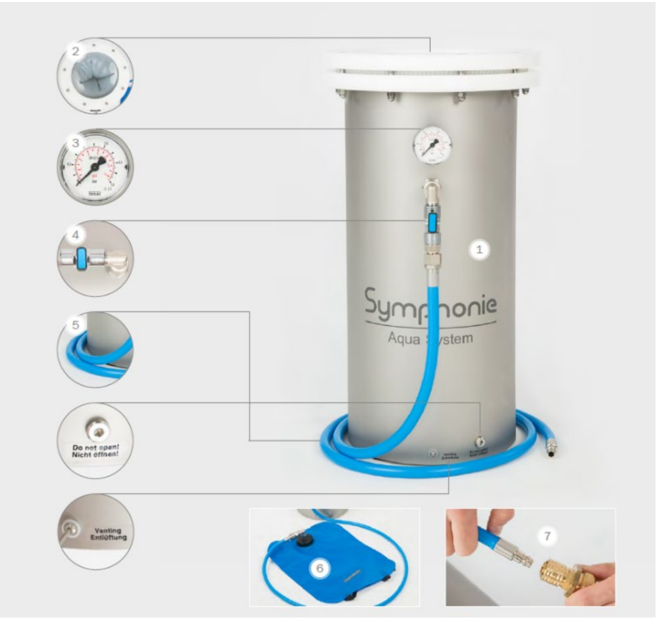

Figure 7 Important components of the Symphonie Aqua System™: (1) pressure vessel, (2) hydrostatic silicone membrane, (3) pressure gauge, (4) stopcock, (5) connecting hose, (6) compensation reservoir, and (7) filling connector. Figure from Romedis gmbh, operating instructions downloadable PDF.

of fabrication and socket fit outcomes using standing pressure casting have been limited to case studies,[6, 14, 20, 22, 24, 25] applications in developing countries,[10, 21] and comparison to the patella tendon bearing socket,[12, 21, 22] which makes up only 8.5% of transtibial lower limb sockets provided in clinical practice in the United States (US) (compared to ~20% each for total surface bearing and hydrostatic sockets).[26]

Modelling of the residual limb-water interface pressures for different loading conditions suggests that there is a positive correlation wherein an increase in load increases the interface pressure.[13] It has been argued that the most effective socket relies on a hydrostatic principle wherein controlled pressure is used to deform tissue under load such that hypothetically the stiffest path principle is achieved and internal shear stresses minimized.[25, 29]

Given the system is used only in the clinic for casting of the residual limb, there are no storage and handling requirements.

The Symphonie Aqua System™ is a commercially available device and will be used in accordance with manufacturer instructions for use as can be found at https://www.romedis.de/assets/upload/pdf/gebrauchsanleitung-symphonie-aqua-vcsystem.pdf and do not intend to submit any of the data to the Food and Drug Administration (FDA). Prior correspondence with the Northwestern University's Institutional Review Board (NU-IRB) indicated that the use of that device in this study would not be regulated by the FDA (see attached email). The US distributor of the Symphonie Aqua System™ also stated in their letter that there is no FDA oversight for this product (see attached letter).

Sockets are custom products that do not require a CE mark. Instead, the INAIL Prosthetic Center operates in accordance with ISO 9001:2008 standards (International Standards Organization).

Since the prosthetic socket is a custom-made device it is considered Class I exempt by the Food and Drug Administration (FDA), hence ***neither Good Laboratory Practice (GLP) nor Good Manufacturing Practice (GMP) Guidelines*** apply. The proposed study will be registered on clinicaltrials.gov and conducted with the oversite of multiple IRBs, which will ensure that it is conducted in a transparent manner while complying with ethical principles and sound scientific evidence as documented in detailed protocols. The process for casting and fitting check sockets is the same as the standard-of-care clinical process that study participants will have previously experienced when receiving a new prosthetic socket. The only difference is use of the Symphonie Aqua System™ for taking the standing hydrostatic pressure cast. Hence, participation in this study involves largely the same risks as those that the subject would experience when having their regular prosthetic socket fabricated and evaluated.

We anticipate that the Symphonie Aqua Casting™ system will be deemed a non-significant risk device and the casting and check socket fitting procedures considered minimal risk by all the IRBs involved. Given the minimal risk nature of this study, we do not anticipate the need for stopping rules.

Clinical Monitoring Plan

We anticipate that the standing hydrostatic pressure casting system will be considered a non-significant risk device and the study minimal risk by all the IRBs involved. Given the minimal risk and low level of study complexity in terms of human subject participation, the trial will be monitored by the PI and IRBs. The lead PI will monitor progress at all sites by engaging in monthly teleconferences with site PIs. This will allow challenges and issues to be discussed, to ensure that protocols are being followed correctly, and that data quality is being maintained. Each site will submit amendments, adverse events and protocol deviations to their respective Institutional Review Boards (IRBs) as needed following the schedule required by each IRB. These events will also be discussed at the monthly teleconference.

PROCEDURES INVOLVED

Research Team Training

Upon commencement of the project, we will develop a Manual of Procedures (MOP). The MOP will detail study conduct and operation, facilitating consistency in protocol implementation and data collection across study sites. The MOP transforms the study protocol into a guideline that describes each step of the study and how it is to be executed. Each study team member will have a MOP.

We will convene a week-long in-person training and study kick-off meeting of key investigators and prosthetists from all three sites. We plan to hold the meeting at the MVAHCS to make sure that all VA prosthetists can attend relevant portions of the meeting while still meeting clinical care needs. This meeting is vital to review study procedures documented in the MOP and provide the training necessary to ensure that all sites implement the protocol consistently. For example, study prosthetists will be trained on the casting techniques and socket designs to be used, while research engineers/post-doctoral associates will be trained as to how to scan casts, positive models and sockets. All prosthetists involved in the study have between 6 and 30 years of experience providing prosthetic clinical care for persons with lower limb amputation. While they all have extensive experience with hand casting, there are mixed levels of experience with standing hydrostatic pressure casting. Hence, the purpose of training is to ensure that everyone is comfortable with each technique and that we use the same approaches across study sites. Finally, the meeting will also provide a venue for the study team to discuss how socket fittings are conducted so that a standardized checklist of Clinical Fit Criteria can be developed with consensus from the team for use in the study. It will be added to each IRB through a modification as a data collection form once it is developed.

To facilitate discussion and development of the Clinical Fit Criteria checklist, we will conduct a literature review and information related to procedures used clinically to evaluate socket fit will be assembled. We know that clinically during static check socket fitting of the NU-FlexSIV Socket as an example, the prosthetist assesses the ability to push the residual limb into the socket and achieve total contact on the distal end. If distal end contact is not achieved, the prosthetist will check the height of the medial brim. If the brim is too high, causing impingement, it is lowered. If the medial brim height is acceptable, then the volume and/or circumference of the proximal third of the socket are checked to ensure they are not too tight. If distal end contact is achieved, but there is gapping laterally or medially, then proximal circumferences are adjusted. Criteria such as this can be collated into a checklist that can be used to assess if the fit of any given socket design is satisfactory or not. The checklist is likely to have 3-5 factors that are either met or not. The goal of improving the shape capture process is to create a socket “right out the gate” that fits well (i.e., meets all the checklist criteria).

Develop Technical Processes

Assessment of socket volume and shape requires scanning and digitizing of casts, positive models and initial sockets. There are multiple points in the process where scans will be taken: initial casts, positive models pre- and post-rectification, and check sockets. We assume that scans of the casts are the same shape as the pre-rectified positive model given that positive models are made by filling the cast with liquid plaster. However, as a quality measure it would be prudent to scan both the casts and the pre-rectified positive model in case deformation occurs. However, we will only process and analyze scans of either the cast or the pre-rectified positive models. Hence, we anticipate that a total of eight scans per subject will be processed and analyzed, either 4 scans of the casts or 4 scans of the pre-rectified positive models, 2 scans of the post-rectified positive models and 2 scans of the sockets (Figure 7 depicts one of these options for simplicity).

Two types of scanners are needed to ensure an accurate scan of the casts (positive and negative): a mechanical laser scanner (Echo Digitizer by Rodin4D, Pessac, France) and a hand-held scanner (Go!Scan3D 50 by Creaform, Quebec, Canada). Mechanical laser scanners are

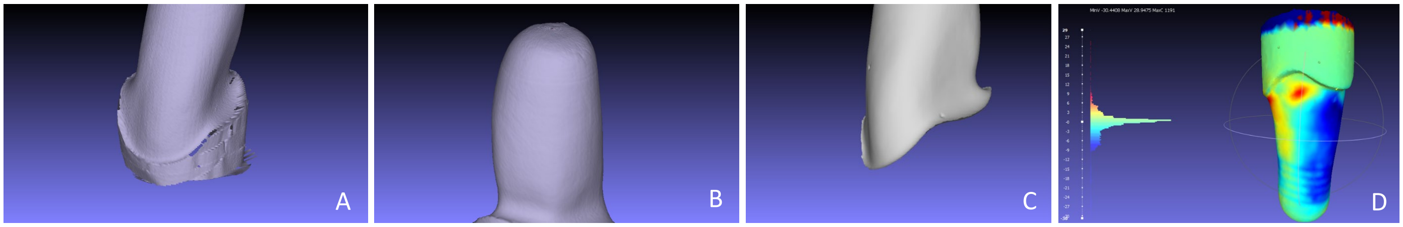

Figure 7 (A) Scan of model with missing distal data. (B) Scan of distal end. (C) Scan of proximal brim. (D) Histogram and color-coded map of shape differences when comparing two scans.

capable of measuring the distal part of a socket well (Figure 7A) but might not be able to capture the proximal part because the diameter of the socket exceeds the field-of-view of the laser if larger than 24cm (Figure 7B). Hand-held scanners are the ideal complement of mechanical laser scanners because they can easily capture large diameters (Figure 7C), but have limitations capturing the distal part of the socket, especially if residual limb length exceeds 35cm. INAIL has successfully developed a procedure to overcome these challenges in scanning by combining laser and hand-held scans. Additionally, INAIL has developed a procedure to compute distance maps of multiple sockets. Hence, de-identified digitized files will be sent by all sites to INAIL for post-processing and calculation of volume and shape.

For a given socket, the following procedure will be used to fuse the point cloud of the mechanical laser scanner and the point cloud of the hand-held scanner into a single surface mesh. The process begins with positioning a set of at least three reflective markers on the socket. These markers must be visible in both scanners' datasets. Then, a spatial registration based on the markers will be completed, followed by an automatic optimization procedure. Subsequently, the best part of each scan will be preserved, and point sampling and remeshing will be run. This innovative procedure, developed by INAIL, have been successfully tested in clinical practice. During the project, the absolute accuracy of the method will be documented to support publications arising from this project.

The spatial registration of negative casts with respect to positive models will firstly exploit a manual or socket feature-based alignment, followed by application of the Iterative Closest Point algorithm.[46, 47] The calculation of distance maps between two surfaces representing the same socket at different stages of fabrication will be based on calculation of the distance between the closest points of the two meshes after registration, followed by the optimization of a color-coded set depending on the absolute signed differences, and on the visualization of a histogram (Figure 7D). A standardized approach for this process will be defined during the project.

The calculation of average and maximum differential maps over multiple sockets (i.e. sockets from different subjects or collected by different prosthetists) is a more challenging mathematical operation, that is expected to require the morphing of each mesh on a template to then proceed with the statistical analysis. Alternatively, assessment of shape changes may be based on the extraction of scalar dimensional parameters in the regions of interest as identified by an expert prosthetist, followed by statistical analysis.

Subject Recruitment

At each site, a convenience sample of adult subjects (i.e., over 18 years) with unilateral lower limb amputation (either transtibial or transfemoral) who are current prosthesis users will be recruited to participate in this study. See Section: Inclusion and Exclusion Criteria for more detail.

Data Collection

Once enrolled, subjects will be evaluated (Visit 1, Figure 6) and the following characteristics collected: age, gender, height, weight, smoking, presence of diabetes, amputation etiology, time since amputation, functional mobility (as defined by the Amputee Mobility Predictor, AMP),[48]

residual limb length and tissue type (soft/firm), sensitivity or painful areas on the residual limb, and current socket/prosthesis design. See Appendix A for more information about the AMP.

All subjects will participate in two additional study visits: a casting visit (Visit 2, Figure 6) and then an initial socket fitting visit (Visit 3, Figure 6). At the casting visit (Visit 2, Figure 6), subjects will be cast twice with each approach in order to assess the inter-rater reliability (i.e., between experienced prosthetists) of each casting technique. The order of the four casts will be randomized for each site using a table of random numbers and all procedures will be timed by a research engineer/post-doctoral associate.

For transtibial subjects, a total surface bearing hand cast will be taken using a circumferential wrap of the residual limb with the subject in a seated position as is typically done in clinical practice. A total surface bearing hydrostatic pressure cast will be taken using a circumferential plaster wrap and then placing the residual limb into the Symphonie Aqua Transtibial System with Vector Control Unit™ while in a weight bearing standing position. Only use of cushion or seal-in liners will be permitted for the purposes of this study. Pin-locking liners (which in the US are used in 21.7% of transtibial prosthetic practice[26]) will be excluded given that they require tissue elongation during the casting process that is not possible when using standing hydrostatic pressure casting with a water cylinder. However, since we are not testing beyond initial fitting, excluding this liner type from the study does not preclude subjects who might otherwise use this liner type from participating in the study, although it may mean they do so in addition to any casting they may need for a clinically necessary socket replacement. Additionally, any total surface bearing socket design can be fabricated with this system.

For transfemoral subjects, a NU-FlexSIV Socket[33] hand cast will be taken using a circumferential wrap of the residual limb with the subject in a seated position. A total surface bearing hydrostatic pressure cast will be taken using a circumferential plaster wrap and then placing the residual limb into the Symphonie Aqua Vector Control Ready Transfemoral System™ while in a weight bearing standing position. Again, only cushion or seal-in liners will be used and pin-locking liners will be excluded (which in the US are used in 12.5% of transfemoral prosthetic practice[26]) but the individuals who typically wear this liner type may still participate in the study. The casting procedure for the NU-FlexSIV Socket[33] has been described and successfully taught to ~200 prosthetists around the world by co-developers Fatone and Caldwell. Clinical implementation is evident in recent presentations[37-42] and publications[49] by other investigators.

Casts taken from all subjects will be scanned. Between the second and third visits (Figure 6), prosthetists will fill the casts with liquid plaster so that we have positive models of all casts that can be scanned using the hand-held scanner. Depending on quality of the scans either the four scans of casts will be used or the four scans of the pre-rectified positive models will be used to assess inter-rater reliability of casting. However, only the lead prosthetist at each site will proceed to rectify the positive models of their casts according to the principles associated with each socket design and casting procedure. Positive models will be scanned again after rectification to allow for comparison of the shape change that occurs for each casting approach as a result of the rectification process. Diagnostic check sockets will then be fabricated for initial fitting using standard clinical fabrication procedures.

Subjects will then return for the initial fitting visit (Visit 3, Figure 6). A research engineer/post-doctoral associate will randomly present the sockets for fitting. Randomization order will be created for each site using a table of random numbers. Subjects and prosthetists will be blinded to the socket condition. Subjects will don the socket and be asked to place as much weight as they can tolerate on the residual limb (recorded using a bathroom scale placed under the socket/residual limb as we have done previously[50]) and the Socket Comfort Score[43] will be administered and recorded by a blinded assessor. This will allow us to assess comfort "right out the gate". Following this assessment, a static diagnostic socket fitting procedure will be conducted for each socket, wherein the prosthetist will evaluate socket fit using the Clinical Fit Criteria checklist. To achieve a satisfactory socket fit, prosthetists may make modifications to the socket.

If the socket fit is deemed to be unsatisfactory, the prosthetist may choose to re-make the socket. Socket modifications and number of re-makes, along with the time taken to achieve a satisfactory socket fit will be recorded. Once the prosthetist and subject are satisfied with the fit, the subject will again be asked to place as much weight as they can tolerate on the residual limb and the Socket Comfort Score will be administered and recorded by a blinded assessor. In keeping with the preliminary data that was used to power the study, comfort achieved in the first check socket is the primary outcome of the study. The final satisfactory socket fittings will be scanned using the mechanical scanner.

DATA AND SPECIMEN BANKING

Not applicable.

SHARING RESULTS WITH PARTICIPANTS

Results of screening and/or study participation **will not** be shared with participants or their primary care provider as it is unlikely that subjects would benefit medically or otherwise from the information.

STUDY TIMELINES

We expect that subjects will participate in this research study for 3 weeks. Table 2 illustrates that we expect it will take us 2 years to enroll all study participants. We estimate that it will take us an additional 6 months after the subjects complete the study to complete the primary analyses (estimated date 9/30/23).

Table 2 Projected quarterly enrollment of human subjects

**Target Enrollment (per quarter)**	**Year 1**				**Year 2**				**Year 3**			
	**Q1**	**Q2**	**Q3**	**Q4**	**Q1**	**Q2**	**Q3**	**Q4**	**Q1**	**Q2**	**Q3**	**Q4**
Site 1 (NU)	0	0	4	4	4	4	4	4	3	3	0	0
Site 2 (MVAHCS)	0	0	4	4	4	4	4	4	3	3	0	0
Site 3 (INAIL)	0	0	4	4	4	4	4	4	3	3	0	0
**Target Enrollment (cumulative)**	**0**	**0**	**12**	**24**	**36**	**48**	**60**	**72**	**81**	**90**	**0**	**0**

INCLUSION AND EXCLUSION CRITERIA

At each site, a convenience sample of adult subjects (i.e., over 18 years) with unilateral lower limb amputation (transtibial and transfemoral) who are current prosthesis users will be recruited to participate in this study. We will exclude anyone with

- poor residual limb sensation,
- a superficial neuroma that is painful to pressure,
- an open sore on the residual limb,
- a residual limb circumference or body weight that exceeds the size or weight limits of the Symphonie Aqua System^{TM} (i.e., >58cm and 170kg for persons with transtibial amputation and >78cm and 170kg for persons with transfemoral amputation, although the height adjustable casting stand used with the transfemoral system has a 500kg limit for safety), or
- inability to stand for the 4-6 minutes required for casting (e.g. persons with bilateral amputations),
- a new amputation given that they have immature residual limbs (i.e., have been an amputee for less than 1 year) that are less stable in volume.

- with transfemoral amputation and a known silicone allergy or a femur length less than 5 inches, since these are contraindications for the NU-FlexSIV and NU-FlexSIS Sockets.

There are no exclusions as to women and minorities.

PARTICIPANT POPULATION(S)

Accrual Number:	Category/Group: (Adults/Children Special/Vulnerable Populations)	Consented: Maximum Number to be Consented or Reviewed/Collected/Screened	Enrolled: Number to Complete the Study or Needed to Address the Research Question
Local	Adults	30	27
Study-wide	Adults	60	54
Total:	Adults	90	81

RECRUITMENT METHODS

Recruitment will take place at each of the three study sites. **NUPOC** is the oldest and largest prosthetics and orthotics research and education facility in the US but does not provide clinical care directly to persons with amputation. However, our research prosthetist Ryan Caldwell maintains an active clinical practice with Scheck & Siress Inc., the largest provider of orthotic and prosthetic clinical care in the Chicagoland area with over 40 certified and licensed orthotic and prosthetic practitioners and twelve accredited facilities. Ryan's clinical practice location provides approximately 85 transtibial and 60 transfemoral prostheses annually, while practitioners at Scheck & Siress more broadly see over 1300 patients with lower limb amputation per year. In addition to Ryan's clinical practice and Scheck & Siress Inc. as a source of potential subjects (see letter of support from Scheck & Siress Inc.), NUPOC also has long-standing relationships with patient models and research subjects who have agreed to be contacted for research projects as well as being located across the street from the Shirley Ryan Ability Lab (formerly the Rehabilitation Institute of Chicago) where an active prosthetics and orthotics clinical service provides an additional resource for subject recruitment. Finally, information about the study in the form of an IRB approved study flyer will be circulated to other prosthetists in the midwest via the Midwest Chapter of the American Academy of Orthotists and Prosthetists, whose members include prosthetists practicing in Illinois, Indiana and Wisconsin. Co-Investigator Ryan Caldwell is on the Board of Directors of the Midwest Chapter and PI Dr. Fatone is a member. Given the large population within the Chicagoland area, the long-standing presence of NUPOC as a research and education facility, our relationships with local organizations, hospitals and prosthetists, as well as a track record of successfully recruiting amputee research subjects for other studies (including Dr. Fatone's currently funded clinical trial evaluating performance and comfort of the NU-FlexSIV Socket, #W81XWH-15-1-0708), we do not anticipate any major issues with recruitment.

The **MVAHCS** is one of seven Regional Amputation Centers (RAC), the flagship facilities within the VA's Amputation System of Care. The MVAHCS is also one of five VA Polytrauma Rehabilitation Centers in the US. The MVAHCS performs about 30-35 transtibial and 10-20 transfemoral amputations each year. About 65-70% of the Veterans with transtibial amputation receive a prosthesis and about 50% of the Veterans with transfemoral amputation receive a prosthesis. Many Veterans with past amputations are followed year to year, with over 600 Veterans with amputations in our RAC database. The prosthetics department at the MVAHCS is a fully-equipped clinic including three full-time prosthetists and a number of support staff. In addition to prosthetists recruiting subjects as they come in for appointments related to a new socket, the MVAHCS study coordinator will screen medical records to find the necessary subjects for the study. We do not anticipate any problems recruiting 20 transtibial and 10 transfemoral Veterans with amputation at the MVAHCS over the three-year study period.

INAIL is one of the largest public, non-profit institutions in Italy. INAIL's activities range from work-related injury prevention, to injured workers' economic compensation, including the direct provision of medical care in addition to standard care provided by the National Healthcare Services. In this framework, INAIL operates a Prosthetic Center that provides about 1200 transtibial and 800 transfemoral prostheses annually. Patients are treated by a multidisciplinary team of physicians, engineers, certified prosthetists, physical therapists, nurses, psychologists and social workers operating within the same facility. Given the volume of patients seen at INAIL, and how easily the proposed comparative assessment fits within the regular clinical workflow, we do not anticipate any problems recruiting subjects at INAIL.

COMPENSATION FOR PARTICIPATION IN RESEARCH ACTIVITIES

Compensation for participation in research activities will be site specific. Please refer to the local protocol addendum for details.

WITHDRAWAL OF PARTICIPANTS

It is possible that the skin on the residual limb may become irritated from multiple socket casting and check socket fittings. The residual limb will be checked frequently during each study visit by the prosthetist to ensure that irritation or rubbing are dealt with as they would be clinically. Any red marks should resolve within a few minutes of cast and check socket removal. If they do not, and if the prosthetist is concerned, then the testing session will be terminated if there are no other means of addressing the irritation or rubbing. This may lead to the PI making a determination to withdraw the subject from the study.

Subjects may also be withdrawn from the study by the PI if they repeatedly fail to attend study visits without notice.

Any data collected up to the time a subject leaves the research study will be kept and used as part of the study.

Since the subjects are not undergoing any study related procedures outside of the study visits, termination procedures are relatively straight forward as all it involves is early processing of payment for study visits attended (if applicable at each site).

RISKS TO PARTICIPANTS

The process for casting and fitting check sockets is the same as the standard-of-care clinical process that study participants will have previously experienced when receiving a new prosthetic socket. The only difference is use of the Symphonie Aqua System^{TM} for taking the standing hydrostatic pressure cast. Hence, participation in this study involves largely the same risks as those that the subject would experience when having their regular prosthetic socket fabricated and evaluated.

(1) The standard clinical process of making a prosthetic socket involves some loss of modesty given the need for the prosthetist to touch the residual limb. This can be attenuated to some degree by ensuring that casting and fitting procedures are conducted in a private room.
(2) It is possible that the new standing hydrostatic pressure casting procedure will be more difficult for the participants than the typical casting procedure, which is conducted in a seated position. The main risk from participating in the study is that they might fall while maneuvering their residual limb into and out of the pressure cylinder during the casting process. We will minimize this risk by providing something for the participant to hold onto while they are standing.
(3) It is also possible that the skin on the residual limb may become irritated from multiple socket casting and check socket fittings. The residual limb will be checked frequently during each study visit by the prosthetist to ensure that irritation or rubbing are dealt with as they would be clinically. Any red marks should resolve within a few minutes of cast and check socket

removal. If they do not, and if the prosthetist is concerned, then the testing session will be terminated if there are no other means of addressing the irritation or rubbing.

(4) This study involves the use of identifiable, personal information and there is a chance that a loss of confidentiality could occur. The research team will make every effort to protect subjects' private health information and guard against any loss of privacy. Study sites will be coded as follows: NUPOC (1), MVAHCS (2) and INAIL (3); and subjects participating in the study will be coded using a six-digit identifier based on the following rubric: site # and subject #. Subjects will be coded sequentially based on enrollment using a two-digit code, e.g., 01, 02, etc. Subjects will be assigned a study code at enrollment and all study related forms/files will be labelled only with the study code. All electronic data will be maintained at each site on password-protected computers located in locked rooms and all paper files will be stored in locked cabinets in locked rooms. Both computers and paper files will be accessible only to authorized study personnel. Forms that require the subject's identity and cannot be coded (e.g. payment paperwork) will be completed at each individual site and not shared between sites. Each site PI will maintain their own master list of study codes in hard copy format to be stored in a locked cabinet in a locked office. Only de-identified data will be shared between study sites. AMRDEC SAFE (Safe Access File Exchange) will be used to share de-identified files with between MVAHCS and the other two sites, since SAFE is considered an acceptable file sharing method by the VA IRB. However, since the AMRDEC SAFE can only be used by nongovernment individuals to send files to *.gov or *.mil email addresses, INAIL and NUPOC investigators will use NU Box to share de-identified files with each other.

Risk Management and Emergency Response

Each site has different procedures for the provision of emergency care:

(1) Northwestern University research staff will call 911 for emergency service if a subject is injured on site during the project. As described in the consent form, Northwestern University will not pay for medical care required because of a bad outcome resulting from participation in this research study. However, this does not keep the subject from seeking to be paid back for care required because of a bad outcome.

(2) MVAHCS will provide treatment for injury to all Veterans who participate in this research study, including first aid, emergency treatment and follow-up care, as needed. In the event an injured Veteran cannot reach a VA facility, the VA will pay for necessary medical care for any injury or illness directly related to participation in this research study. If participating Veterans receive this type of medical care, they must contact the VA site PI, Dr. Andrew Hansen using the contact information provided in the section of the VA consent documents titled "Compensation for Any Injuries". The participant does not release the VA Medical Center from liability by signing the consent form. Compensation for free medical care other than as described in the consent form, payment of lost wages, or compensation for pain and suffering may be available from the VA under applicable Federal Law. Study participants should immediately report any injuries resulting from their participation in this study to the VA site PI, Dr. Andrew Hansen.

(3) INAIL prosthetists are Basic Life Support and Defibrillation (BLSD) trained. This means that in case of emergency, an internal in-house emergency team can be reached. The team includes a nurse and practitioner who are BLSD instructors. INAIL has 24-hour medical and nursing staff in-house. A unified emergency number equivalent to 991 in the United States can be used on any phone. Use of any hospital emergency room is free-of-charge throughout Italy. INAIL has liability insurance for standard clinical care. The budget assigned to cover the IRB costs for INAIL for this project includes costs for specific insurance in case it is required by the IRB for this project.

POTENTIAL BENEFITS TO PARTICIPANTS

Possible benefits of study participation include a more comfortable check socket since that is the intent of developing a better casting technique. Since the check sockets made for this study are made to uniquely fit each individual participant, they will be allowed to keep the one that fits best at the end of the study. However, they would need to take that check socket to their regular prosthetist in order to continue the process of receiving a new socket based on that fitting. The research team is not responsible for anything the subject chooses to do with the check socket after study completion.

This study will show whether or not standing hydrostatic pressure casting using a water cylinder offers a consistent and efficient alternative to conventional hand casting for prosthetic socket fabrication that results in a more comfortable socket “right out the gate”. Such evidence is needed to support the adoption of new technologies for persons living life with a lower limb amputation. Given the potential to generate evidence for a process that may provide a more comfortable fitting socket, the minimal risk is justified.

DATA MANAGEMENT AND CONFIDENTIALITY

Power Analysis

Using the previously described pilot data wherein persons with transtibial amputation used the Socket Comfort Score[43] to assess sockets fabricated using both casting techniques, we ran a range of power and sample size calculations using STATA MP 15 software (StataCorp. LLC, College Station, TX). Based on our pilot data, means were 8.4 and 6.3 for sockets made with each casting technique, with standard deviations of 1.3 and 2.8, respectively. The correlation between the two socket methods was 0.4. Keeping 0.8 power and 0.05 alpha constant, we looked at the total sample size that would be required to detect a difference of 3-, 2- and 1-points in Socket Comfort Score. Assuming a dependent 2-sided t-test framework, the total N to detect a 1-point difference was 54, to detect a 2-point difference was 16, and to detect a 3-point difference was 8. Although having 3 sites is unlikely to affect our power and sample size calculations we will explore differences by site in our analysis and adjust for site if needed. Recent assessment of the test-retest reliability of the Socket Comfort Score over 2-3 days suggests that the minimal detectable change is 2.73.[51] However, since our testing will take place side by side in a single study visit, we would like to have sufficient power to detect a 1-point difference in Socket Comfort Score. We think that this is the minimal difference that will be meaningful for our testing scenario and, when put in terms of standard deviation units of change, it represents a small to medium effect size. We expect drop-outs to be modest given the minimal time and burden of this randomized crossover trial, but to err on the side of caution we propose to enroll 20 transtibial subjects at each site for a total of N=60. This number allows for 10% attrition.

We do not have similar pilot data upon which to base a power calculation for transfemoral subjects. Hence, we used the above power calculations in combination with knowledge of the lower number of persons with transfemoral amputation as a proportion of the amputee population to determine a sample size.[52] A total sample size of 16 transtibial subjects is needed to detect a two-point change in Socket Comfort Score, which equates to six subjects per site (rounding up slightly). However, we will recruit 10 transfemoral subjects per site for a total of N=30. Therefore, allowing for 10% attrition, this number is still 36% more than the available data suggests is needed.

Statistical and Data Analysis Plan

Since the study has been powered for each amputation level, we will be able to assess each hypothesis for each amputation group as well as for the groups combined using the same

analyses described below. Additionally, our statistician will be involved in the study throughout all three years, allowing for interim analyses as data accrues.

To assess ***hypothesis 1.1*** that standing hydrostatic casting with a water cylinder results in more consistent cast shape and volume compared to hand casting, a research engineer/post-doctoral associate will scan the casts, positive models (pre- and post-rectification) and initial sockets once a satisfactory fit has been achieved. Scans from each step in the shape capture and socket fabrication process will be used to assess how the captured shape changes over time. Since one proposed advantage of standing hydrostatic pressure casting with a water cylinder compared to hand casting is that it captures a weight bearing shape that requires minimal rectification, the shape captured should remain more consistent from cast to socket than for the hand casting process with involves manipulation of the shape first by hand during casting and then during rectification.

Consistency of the standing, hydrostatic pressure casting with water cylinder will also be evaluated by assessing the inter-prosthetist difference. Hence, a research engineer/post-doctoral associate will scan the casts taken by two prosthetists using each casting procedure. The scans will be digitized and used to assess the difference between prosthetists for each casting procedure; and then we will compare the difference between casting procedures.

There are a number of potential approaches to quantifying and analyzing differences in scanned shapes. For example, color maps with average histograms detailing the differences between matching points on the mesh between shapes captured at different points in time, such as that shown in Figure 7D, can be derived. Statistical comparisons of shapes may include comparisons of the distribution of differences in shapes, maximum differences in shapes for specific regions, and/or complete shape comparisons using a Shape Comparison algorithm in R Software (freely available from R Foundation). At the beginning of the project and in parallel with IRB and HRPO approvals, Dr. Cutti will work with Mrs. Gravely (study statistician) to determine the most appropriate statistical approach for statistical shape comparison. More challenging, but possible would be to scale each scan to a template (e.g. generalized limb model similar to those included in CAD-CAM socket rectification software) using morphing software (e.g. rbf-morph, RBF Morph srl., Rome, Italy), matching all the point differences between template and scan to calculate means and standard deviations on a point by point basis. For example, we would take the unrectified and rectified models for each subject, match the models using the Iterative Closest Point algorithm and calculate distances associated with rectification. We would then take the differences associated with rectification from each subject and scale them to a single socket template, first by using corresponding anatomical landmarks, then using morphing. This brings the distance map of each subject into alignment with the same template. Finally, we would calculate differences for each point in the mesh. This would create very fine shape maps for each subject that may be easier to compare across subjects and potentially have the added benefit of resulting in a CAD-CAM template for use with sockets made with the Symphonie Aqua System™. If these approaches to assessment of shape are unsuccessful, we can use variables typically calculated in software that is used for the digital rectification of limb models (e.g. volume, length, diameters and circumferences) to assess discrete changes in shape during the casting, rectification and socket fitting process.

To assess ***hypothesis 2.1*** that standing hydrostatic pressure casting with a water cylinder reduces the time required to cast, rectify and successfully fit an initial diagnostic check socket compared to traditional hand casting, a research engineer/post-doctoral associate will time each step in the socket fabrication process (casting, rectification and initial fitting). The total time to achieve a satisfactory initial socket fitting will be assessed using a Generalized Estimating Equations (GEE) model[53] to compare time between the two groups. The simplest GEE model will resemble a paired t-test, but if paired t-test assumptions are not met, GEE models allow us to accommodate almost any distribution for the dependent variable (ordinal, dichotomous, gamma, and so forth). These will be fit in SAS PROC GENMOD software (SAS Institute Inc., Cary, NC).

To assess ***hypothesis 2.2*** that standing hydrostatic pressure casting with a water cylinder reduces the number of check sockets required to achieve a clinically acceptable fit compared to traditional hand casting, a research engineer/post-doctoral associate will document modifications descriptively and socket iterations required to reach what the prosthetist and subject consider a satisfactory fit for each socket. The number of socket iterations will be assessed using GEE models to compare socket iterations between casting methods. We will start with the 'number of iterations' as a count or potentially categorize it into 2 or 3 categories depending on the range of iterations. GEE models can account for both count and categorical outcomes. These will be fit in SAS PROC GENMOD software (SAS Institute Inc., Cary, NC).

To assess ***hypothesis 3.1*** that standing hydrostatic pressure casting with a water cylinder improves socket comfort as compared to hand casting, a blinded assessor will administer the Socket Comfort Score in full weight bearing for each socket before and after the subject and prosthetist have made any modifications needed to achieve a satisfactory initial fit. Order in which sockets are fit/assessed will be randomized. Socket comfort is the primary outcome of the study, as we believe it is most important to the prosthesis user and the variable for which we powered the study.

Pain-measurement methods such as the numerical rating scale (NRS) were used by Hanspal et al.[43] as models for quantifying and communicating socket comfort. The Socket Comfort Score asks the standard question, *"On a 0 to 10 scale, if 0 represents the most uncomfortable socket fit you can imagine, and 10 represents the most comfortable socket fit, how would you score the comfort of the socket fit of your artificial limb at the moment?"*[54] The developers of this measure reported inter-rater reliability, criterion-related validity, sensitivity to change, and utility in clinical practice in 44 consecutive persons with lower limb amputation.[54] As part of development of the measure, a treating prosthetist collected Socket Comfort Score responses three times during the course of a single day, as did an independent prosthetist, and a physician. Their ratings were highly correlated (Kendall tau 0.97–0.98, p<.001). Additionally, Socket Comfort Score responses were significantly correlated with assessments of socket fit by the treating prosthetist and with residual limb health by a physician (Kendall tau 0.51 and 0.48, p<.001). There was sensitivity to change, with 76% (n = 22) of adjusted sockets demonstrating a significant improvement in the Socket Comfort Score of 1 to 5 points (Wilcoxon z = 74.16, p<.001). The Socket Comfort Score is a simple, easy-to-administer measure that allows clinicians to quantify change in socket fit rather than relying on descriptive terms.[7, 44, 55] More recently, Hafner et al.[51] reported that the test-retest Intraclass Correlation Coefficients (ICC) for the Socket Comfort Score administered to lower limb amputees 2-3 days apart ranged from 0.63 to 0.79, depending on mode of administration, indicating that the Socket Comfort Score is appropriate for group-level comparisons when administered in a single mode.

Hence, Socket Comfort Score at initial fit will be compared between sockets fabricated with each casting approach using GEE models, which account for both correlation and any type of outcome variable. The simplest GEE model will resemble a paired t-test, but if paired t-test assumptions are not met, GEE models allow us to accommodate almost any distribution for the dependent variable (ordinal, dichotomous, gamma, and so forth). These will be fit in SAS PROC GENMOD software (SAS Institute Inc., Cary, NC).

To assess ***hypothesis 3.2*** that standing hydrostatic pressure casting with a water cylinder improves socket fit assessed clinically as compared to hand casting, the prosthetist will document the fit of each socket before and after any modifications using the Clinical Fit Criteria checklist that we intend to develop at the initial kick-off and training meeting. Order in which the sockets are fit/assessed will be randomized. The checklist will likely have 3-5 factors that are either met or not. We will use a McNemar's test (i.e., a chi square test for dependent measures) for each factor to assess frequency with which each casting approach created a socket "right out the gate" that met these checklist criteria.

Potential Problems and Alternative Approaches

We hope we have addressed potential problems and provided alternative approaches in the preceding paragraphs, however we summarize these here with respect to potential problems and alternative approaches for data processing, statistical analysis and subject loss to follow up.

- To develop the scan assessment process, we proposed a preferred approach based on the calculation of average and maximum differential maps over multiple sockets that is expected to require the morphing of each mesh on a template to then proceed with the statistical analysis. However, if that proves to be too challenging a mathematical problem, we suggested an alternative approach wherein assessment of shape changes may be based on the extraction of scalar dimensional parameters in the regions of interest as identified by an expert prosthetist, followed by statistical analysis.
- To assess rectifications by comparing changes in positive model shapes, we described a number of potential approaches to quantifying and analyzing differences in scanned shapes including: (1) using color-coded maps with average histograms detailing the differences between matching points on the mesh between shapes captured at different points in time; (2) the more challenging option of scaling each scan to a template using morphing software and matching all the point differences between template and scan to calculate means and standard deviations on a point by point basis; and (3) using variables typically calculated in software that is used for the digital rectification of residual limb models to assess discrete changes in shape during the casting, rectification and socket fitting process.
- To assess ***hypotheses 2.1 and 3.1,*** we will use Generalized Estimating Equations (GEE) model given that they allow us to accommodate almost any distribution for the dependent variable.
- To assess ***hypothesis 2.2,*** we will start with the number of check socket iterations as a count but if that does not work, we can potentially categorize the results into two or three categories depending on the range of iterations. Again, GEE models will be used as they can account for both count and categorical outcomes. It is also possible that prosthetists may find it necessary to sometimes recast the residual limb. Hence, we will also track how many times a re-cast is necessary in addition to check socket iterations and ensure that this is also captured in the analysis.
- We expect drop-outs from the study to be modest given the minimal subject commitment required (approximately 9 hours of time spread over 3 visits over 3 weeks), but to err on the side of caution we propose to enroll more subjects than needed, which should be sufficient to allow for 10% attrition.
- If recruiting from among patients known to the prosthetists involved in the study proves to be an insufficient recruitment process in terms of timeliness or number of participants, more proactive recruitment methods will be engaged with IRB approval, such as mailing information about the study to patients with unilateral transtibial or transfemoral amputation who receive clinical services at each site. Similarly, to further facilitate recruitment at their site, NUPOC can, with IRB approval, circulate information about the study to other prosthetists in the midwest via the Midwest Chapter of the American Academy of Orthotists and Prosthetists, whose members include prosthetists practicing in Illinois, Indiana and Wisconsin. Those prosthetists may then share study information with their patients and refer interested potential subjects to PI Fatone for enrollment.

Data Security

Data collection will take place at all three study sites (NUPOC, MVAHCS and INAIL) but data will be pooled across sites for analysis. Data processing will take place primarily at INAIL and statistical analysis primarily at MVAHCS. Transport of samples is not required. However, de-

identified data will be shared between sites as described in the section Data Sharing between Sites so that analysis of scans for all subjects can be conducted at INAIL and statistical analysis can be conducted at MVAHCS.

The research team will make every effort to protect subjects' private health information and guard against any loss of privacy. Study sites will be coded as follows: NUPOC (1), MVAHCS (2) and INAIL (3); and subjects participating in the study will be coded using a six-digit identifier based on the following rubric: site # and subject #. Subjects will be coded sequentially based on enrollment using a two-digit code, e.g., 01, 02, etc. Subjects will be assigned a study code at enrollment and all study related forms will be labelled only with the study code. Forms that require the subject's identity and cannot be coded (e.g. payment paperwork) will be completed at each individual site and not shared between sites. Each site PI will maintain their own master list of study codes in hard copy format to be stored in a locked cabinet in a locked office.

Subjects will be assigned a study code at enrollment and all study related forms/files will be labelled only with the study code. All electronic data is maintained at each site on password protected computers located in locked rooms and all paper files are stored in locked cabinets in locked rooms. Both computers and paper files are accessible only to authorized study personnel.

The following people can review study records: Institutional Review Board (IRB) approved study researchers and staff, representatives of the Department of Defense (including USAMRMC and HRPO), and the IRBs at each participating site. They are all required to keep the subject's personal information confidential. Sensitive information will be reported to state or local authorities only if required by law and will follow the procedures of each institution's privacy officers.

All electronic data is maintained at each site on password-protected computers located in locked rooms and all paper files are stored in locked cabinets in locked rooms. Both computer and paper files will be accessible only to authorized study personnel. There are no plans to destroy the de-identified data set. Identifiers will be held and destroyed according to each institution's data management plans.

Data Sharing between Sites

Only de-identified data will be shared between the study sites. Since some data will be VA data, all data will be de-identified in accordance with the Health Information Privacy and Portability Act (HIPAA) Privacy Rule as outlined in Veterans Health Administration (VHA) Handbook 1605.1, Appendix B. Transmission and transfer of VA de-identified data will be performed in accordance with VA security policies. Specifically, de-identified VA data will be shared with other study sites using AMRDEC SAFE (Safe Access File Exchange). The SAFE application can be accessed online and used to send large files to individuals that would normally be too large to send via email. SAFE supports file sizes up to 2GB. There are no user accounts for SAFE - authentication is handled via email. SAFE is considered an acceptable file sharing method by the MVAHCS Institutional Review Board (IRB).

However, since the AMRDEC SAFE can only be used by nongovernment individuals to send files to *.gov or *.mil email addresses, INAIL and NUPOC investigators will use NU Box to share de-identified files with each other.

Data Sharing with Research Communities

Data generated by the funded research will be made available to the research community and to the public under the auspices of the PI. Prior to sharing, data will be de-identified and redacted to reduce the risk of subject identification. Final research data will include the factual material necessary to document and support research findings such as the computerized scans of casts and sockets, outcome measure results, and subject characteristics upon which any accepted publications are based, it may include both raw data and derived variables. Documentation will also be provided regarding the methodology and procedures used to collect the data, details

about codes, definitions of variables, variable field locations, frequencies, etc. No analytic tools will be provided.

Data will be shared no later than the acceptance for publication of the main findings from the final dataset. Data will be shared using Northwestern Box, a file sharing service that provides individual, flexible online space for unlimited file storing or sharing by faculty at Northwestern University. The PI will share data upon request with anyone via a unique link to a file or folder within Northwestern Box. Additionally, a link may also be placed on the research project page on the NUPOC website. A data-sharing agreement will be used to ensure that the data is only used for the purpose described, users acknowledge the data source in any use of the data, agree to provide the PI with copies of any presentation or publication that uses the data, prohibit sharing of the data with others without those parties having their own data use agreement, and prohibit manipulation of data for the purposes of identifying subjects.

Results of the proposed work will be published in peer-reviewed journals and presented at national and international meetings. Publications and presentations will contain summary statistics.

Data Capture, Verification and Disposition:

There are no plans to destroy the de-identified data set. Identifiers will be held and destroyed according to each institution's data management plans. For example, the MVAHCS will hold identifiable data for a period defined by the Department of Veterans Affairs Records Control Schedule and these data will then be destroyed. INAIL will keep data for 7 years after the project ends.

In terms of quality control, when we submit scans to INAIL for processing, INAIL will verify that data are readable and represent what is expected. Also, at each site a calibration tool will be regularly scanned to make sure that scanners are not distorting dimensions.

PROVISIONS TO MONITOR THE DATA TO ENSURE THE SAFETY OF PARTICIPANTS

Not applicable as study does not involve more than minimal risk.

PROVISIONS TO PROTECT THE PRIVACY INTERESTS OF PARTICIPANTS

The standard clinical process of making a prosthetic socket involves some loss of modesty given the need for the prosthetist to touch the residual limb. This can be attenuated to some degree by ensuring that casting and fitting procedures are conducted in a private room with as few individuals present as is necessary to conduct the tasks required (e.g. prosthetist and research staff member who will time the procedures).

This study involves the use of identifiable, personal information and there is a chance that a loss of confidentiality could occur. The research team will make every effort to protect subjects' private health information and guard against any loss of privacy. Study sites will be coded as follows: NUPOC (1), MVAHCS (2) and INAIL (3); and subjects participating in the study will be coded using a six-digit identifier based on the following rubric: site # and subject #. Subjects will be coded sequentially based on enrollment using a two-digit code, e.g., 01, 02, etc. Subjects will be assigned a study code at enrollment and all study related forms/files will be labelled only with the study code. All electronic data will be maintained at each site on password-protected computers located in locked rooms and all paper files will be stored in locked cabinets in locked rooms. Both computers and paper files will be accessible only to authorized study personnel.

Forms that require the subject's identity and cannot be coded (e.g. payment paperwork) will be completed at each individual site and not shared between sites. Each site PI will maintain their own master list of study codes in hard copy format to be stored in a locked cabinet in a locked office. Only de-identified data will be shared between study sites. AMRDEC SAFE (Safe Access File Exchange) will be used to share de-identified files with between MVAHCS and the other two sites, since SAFE is considered an acceptable file sharing method by the VA IRB. However, since

the AMRDEC SAFE can only be used by nongovernment individuals to send files to *.gov or *.mil email addresses, INAIL and NUPOC investigators will use NU Box to share de-identified files with each other.

COMPENSATION FOR RESEARCH-RELATED INJURY

Compensation for research-related injury will be site specific. Please refer to the local protocol addendum for details.

ECONOMIC BURDEN TO PARTICIPANTS

Economic burden to participants will be site specific. Please refer to the local protocol addendum for details.

CONSENT PROCESS

Screening Procedures

Screening will be accomplished by asking each potential subject to verbally confirm that they meet the following selection criteria: that they are over the age of 18 (i.e., an adult), have had a single below- or above-the-knee amputation for over a year, currently use a prosthesis, do not have any numb or pressure sensitive spots on their residual limb, do not currently have an open sore on the residual limb, and are able to stand for 4-6 minutes on their intact limb. With respect to persons with transtibial amputation we will ask if they have a residual limb circumference <58cm and body weight <170kg. For persons with transfemoral amputation we will ask if they have a residual limb circumference <78cm and body weight <170kg. With respect to persons with transfemoral amputation we will also ask if they have any allergies to silicone or a femur longer than 5 inches. If potential subjects verbally indicate they meet these selection criteria they will be enrolled in the study. The prosthetist will confirm selection criteria at the first study visit when they physically evaluate the subject and their residual limb.

Consent Procedures

A Master informed consent form has been drafted, in English and will be converted into Italian for use at INAIL. Each site will tailor the master consent form to site specific requirements.

The PI or other IRB approved study personnel will read the consent form with the subject, verifying that the subject understands the information, and answering any questions the subject may have about the study. Prospective subjects can take the consent forms home for further perusal. Subjects may have as much time as they like between being informed of the study and being consented to take part in the study. Decisional capacity to consent to clinical research will be assessed before a signature is obtained on the consent form using the University of California San Diego Brief Assessment of Capacity to Consent (UBACC).[56] See Appendix A for more information about the UBACC. After all signatures are collected, the subject will be provided a copy of the consent form. Since the study only lasts three weeks, it will not be necessary to re-consent subjects or re-evaluate them for ongoing capacity to provide consent.

For all sites, the consent process will take place prior to beginning any study related procedures. It may occur prior to or at commencement of the first study visit. It will take place at each study site in a quiet location that affords the potential participant some privacy.

We will not recruit minors and other populations who cannot provide informed consent.

NON-ENGLISH SPEAKING PARTICIPANTS

Whether or not participants will include those who do not speak English will be site specific given the involvement of an international site. Please refer to the local protocol addendum for details.

PROTECTED HEALTH INFORMATION (PHI AND HIPAA)

Whether or not protected health information will be accessed will be site specific. Please refer to the local protocol addendum for details.

QUALIFICATIONS TO CONDUCT RESEARCH AND RESOURCES AVAILABLE

Qualifications of Study Personnel

Stefania Fatone, PhD, BPO(Hons), Principal Investigator (PI), Northwestern University. Dr. Fatone is a Professor in the Feinberg School of Medicine, Department of Physical Medicine and Rehabilitation. She is also a qualified prosthetist/orthortist. She has 20 years of experience in prosthetics and orthotics research and over 80 publications. She has been PI on a dozen projects funded by the National Institute on Disability and Rehabilitation Research (NIDRR), DOD, and the National Institutes of Health (NIH) as well as industry and various foundations.

Ryan Caldwell, CP, FAAOP, Co-Investigator/Research Prosthetist, NUPOC. Mr. Caldwell is a certified prosthetist who maintains a one-day-per-week appointment as a research prosthetist with NUPOC in the Department of Physical Medicine and Rehabilitation. Mr. Caldwell is the co-developer of the NU-FlexSIV Socket and has over 15 years of experience providing prosthetic care to persons with lower limb amputation as part of his prosthetic clinical practice at Scheck & Siress Inc. He will be responsible for all patient evaluation and casting at NUPOC.

Jessica Yohay, BS, Study Coordinator, NUPOC. Ms. Yohay has an undergraduate degree in engineering and has provided research support to Dr. Fatone for over three years. She will be responsible for scanning of all casts and sockets and ensuring that files are configured appropriately for processing and analysis. She will also assist in data collection with regards to timing of processes and final evaluation of socket fittings. She will act as the study coordinator, assisting with preparation and maintenance of IRB approvals, clinical trial registration and maintenance, scheduling of subjects, and collating all data files.

Andrew Hansen, PhD, Site PI, Minneapolis VA Health Care System (MVAHCS). Dr. Hansen is the Founder and Director of the Minneapolis Adaptive Design & Engineering (MADE) Program at the MVAHCS and a Professor of Rehabilitation Medicine at the University of Minnesota. He is a biomedical engineer with over 20 years of experience in prosthetics and orthotics research. His work has led to over 60 peer-reviewed publications and 18 issued patents.

Amy Gravely, MA, Co-Investigator/Research Service Biostatistician, MVAHCS. Mrs. Gravely has worked at the MVAHCS as a statistician since 2006. She will lead statistical analyses for the project. Data from all sites will be transferred to Mrs. Gravely, allowing for statistical modeling and hypothesis testing.

John Looft, PhD, Director of Motion Analysis, MVAHCS. Dr. Looft completed his PhD in biomedical engineering at the University of Iowa in 2014, as well as post-doctoral fellowships at University of Melbourne in 2015 and the University of Minnesota in 2018. Dr. Looft is now the Director of Motion Analysis at the MVAHCS. He has worked for the prosthetics department since June 2018; he also performs research and development at the MVAHCS.

Kyle Barrons, MSPO, CPO, Prosthetist, MVAHCS Regional Amputation Center. Mr. Barrons is a full-time prosthetist at the MVAHCS and the Advanced O&P Practitioner for the facility. He has been working in the prosthetics field for 8 years, and has been certified in prosthetics by the American Board for Certification in Prosthetics, Orthotics and Pedorthics (ABC) since 2012.

Karl Koester, CP, Prosthetist, MVAHCS Regional Amputation Center. Mr. Koester is the full-time prosthetics laboratory chief at the MVAHCS. He has 10 years of clinical experience as a certified prosthetist.

Brian Arndt, CPO, Prosthetist, MVAHCS Regional Amputation Center. Mr. Arndt is a full-time prosthetist at the MVAHCS and the Assistant Laboratory Chief of Prosthetics. He has been working in the prosthetics field for 13 years, first as a technician/fitter. He has been certified as a prosthetist for the last 6 years.

Nicole Walker, MS, Research Prosthetist Orthotist, MADE Program. Ms. Walker is a full-time research prosthetist-orthotist in the MADE Program, who recently received her masters in prosthetics and orthotics from Baylor University.

Matthew Larson, RN MHI, Nurse Informaticist, Extended Care & Rehabilitation. Mr. Larson will provide access to Regional Amputee Center (RAC) data using an automated report that pulls information from Corporate Data Warehouse (CDW). The data in CDW is updated daily, allowing MVAHCS to find new potential participants as they enter the RAC, as well as hundreds of existing patients who have been seen by the RAC.

Andrea Giovanni Cutti, PhD, Site PI, INAIL Prosthetic Center, Italy. Dr. Cutti is Applied Research Manager at the INAIL Prosthetic Center, a specialized center of the Italian Workers' Compensation Authority focused on the construction and application of prostheses and orthoses and assistive devices. Dr. Cutti is an electronic engineer with a PhD in biomedical engineering. He was also received his prosthetics and orthotics certification in 2018. His research activities have focused on the biomechanics of amputation and upper-limb prosthetics, with over 35 peer-reviewed publications.

Gianluca Migliore, CPO, Chief of the Transfemoral Department at INAIL. Mr. Migliore has nearly 30 years of experience in the treatment of patients with amputations. In particular, his experience in the treatment of persons with transfemoral amputation will be key to the project, thanks to his knowledge of hand casting and of the Symphonie Aqua System™.

Giovanni Osti, CPO, Chief of Transfemoral Casting at INAIL. Mr. Osti's activity has focused constantly on persons with transfemoral amputation over the past 20 years, with specific responsibility for casting over the past 10 years. Mr. Osti's extensive knowledge of all hand-casting procedures is key to the project, together with his direct involvement in fitting over 60 patients with the transfemoral prototype version of the Symphonie Aqua System™ in trials at INAIL. He will lead all activities related to the transfemoral subjects.

Gianni Giorgio Gregori, CPO, Chief of the Transtibial Department at INAIL. Mr. Gregori has 18 years of experience in treating patients with transtibial amputation. He is the chief of the transtibial department, which produces over 1200 transtibial prostheses yearly. His knowledge extends from all hand-casting methodologies to the clinical application of the Symphonie Aqua System™, which he helped previously test.

Facilities, Existing Equipment and Other Resources

The Northwestern University Prosthetic-Orthotic Center (NUPOC) Education & Research is part of the Department of Physical Medicine and Rehabilitation, Feinberg School of Medicine, and located on the Chicago campus of Northwestern University at 680 N. Lake Shore Drive, Suite 1100, Chicago, IL. NUPOC has a total of 20,000 square feet of purpose designed space that is fully accessible to individuals with disabilities (i.e., no physical barriers are present that require any more than minimal effort to overcome). Space is divided between the education and research programs. NUPOC is the oldest and largest research and training program for orthotists and prosthetists in the United States. NUPOC research projects are primarily concerned with addressing clinically relevant problems in the field of prosthetics and orthotics rehabilitation. The NUPOC education program trains new prosthetists and orthotists and conducts continuing professional education courses in prosthetics and orthotics for physicians and therapists as well as for prosthetists and orthotists. Approximately 48 prosthetic and orthotic students graduate from NUPOC each year, representing nearly half of all prosthetic and orthotic graduates in the United States. NUPOC laboratories and facilities include:

- *Prosthetic and Orthotic Rehabilitation Technology Assessment Laboratory (PORTAL):* The PORTAL is used for the evaluation of upper and lower extremity prosthetic and orthotic devices, both commercial and experimental. Within PORTAL there are three specialty lab areas:

- *The Functional Physiology Testing Laboratory:* This laboratory is equipped with an XSens Inertial Measurement Unit system to capture instantaneous body segment accelerations and rotations, a K5 portable metabolic measurement system (Cosmed, Rome, Italy) and a Cosmed Sport Treadmill (T170) with a large belt surface, safety harness, incline/decline capabilities, and adjustable handrails.
- *The Mechanical Testing Laboratory:* This laboratory contains an Instron 8800 Materials Testing Machine (Instron Corp, Norwood, MA) used to assess the mechanical properties of components and materials. This system uses hydraulic actuators to apply loads cyclically to the specimens. These applied loads are controlled using feedback loops and measured using either a 10,000-pound, 1,000-pound, or 110-pound load transducer. A data acquisition program connected to the Instron system acquires the loading response information and creates stress-strain diagrams for a specimen. Using custom adaptors and fixtures, this system can perform a variety of mechanical tests.
- *The Computer-Aided Design and Manufacturing (CAD-CAM) Laboratory:* This laboratory is equipped with a Stratasys Fused Deposition Modeler (FDM) 400mcTM. This rapid prototyping machine uses data in the form of stereo lithography (STL) files that computers can use to make 3-dimensional objects. Fabrication is fully automated and uses a dispensing nozzle to deposit thin layers of liquefied plastic that quickly hardens into a solid. This equipment allows researchers to rapidly fabricate prototypes, thus enabling early error correction and prediction of end-product performance.

- *Plaster and Plastics Fabrication Laboratory:* This laboratory contains three PDQ infrared ovens (OTS-Corp, Weaverville, NC), each with a 36” x 48” capacity and a Rollabout™ tray, a vacuum system and sinks with a plaster trap. This gives us the capacity to do all the casting and socket fabrication we need for our research and development projects.
- *Actuator and Control Laboratory:* This laboratory is a mechanical and electronics facility with capability for prototype electromechanical and mechatronic construction. We have a full range of components, bench power supplies, signal generators, multimeters, voltage reference and oscilloscopes, including a Tektronix 2230 100 MHz digital storage oscilloscope and a Tektronix 2214 digital storage oscilloscope. This laboratory also has precision tools for hand finishing and assembly of mechanical components and a Bausch and Lomb 2x-30x stereo microscope.
- *Machine Shop:* The shop includes drill presses, bandsaws, Trautman routers, belt sanders, grinding wheels, buffing wheels, and an abrasive blaster.
- *Jessie Brown VA Medical Center-Motion Analysis Research Laboratory (JBVAMC-MARL):* NUPOC includes a VA-sponsored research program under the auspices of the Jesse Brown VA Medical Center (JBVAMC). Housed within NUPOC and covered by a partial VA off-site waiver, the JBVAMC-MARL is a 1200 square foot state-of-the-art human movement research laboratory designed for making measurements necessary to quantify human movements. It consists of 12 digital real-time cameras (Motion Analysis Corporation, Santa Rosa, CA), 6 force-plates (Advanced Mechanical Technology Inc., Watertown, MA) embedded in the floor, *pliance* and *pedar* pressure measurement systems (Novel gmbh, Munich, Germany), Trigno 16 channel wireless electromyography (EMG) system (Delsys Inc., Boston, MA), 8-channel telemetered EMG system (Noraxon USA Inc., Scottsdale, AZ), and a Balance System™ SD (Biodex, Shirley, NY). Two force-plates are also available for stairway or slope ambulation. The JBVAMC-MARL also has a custom-instrumented treadmill (N-Mill, MotekForce Link, Culemborg, the Netherlands) used to simulate slips and trips during walking for evaluating locomotor stability. An overhead harness ensures safety of research participants while on the treadmill. OrthoTrak and/or GaitTrak gait analysis software (Motion Analysis Corporation, Santa Rosa, CA) and Visual 3D (C-Motion Inc., Germantown, MD) are available to process gait data. Lastly, the JBVAMC-MARL has a digital video subsystem comprised of two digital

camcorders, a digital media recorder (DMR), a monitor, and a video-editing board that are used for recording split-screen displays of two simultaneous views of research subjects during experiments. All of the measurement systems in JBVAMC-MARL are integrated to allow for the synchronized collection of data in order to provide a comprehensive overview of the particular human movement activity of interest to each study.

In addition to these facilities, Dr. Fatone has a private office equipped with a direct phone line, computer, and internet access. As faculty in the Feinberg School of Medicine, Dr. Fatone also has access both physically and digitally to the resources of Northwestern University such as Blue Jeans Conferencing Service and Northwestern Box. Blue Jeans is a vendor-agnostic, cloud-based audio/video/content sharing conferencing service that supports high-resolution videoconferencing (720p); high-resolution content sharing (up to 1080p); and real time video sharing. Live chat is available during meetings and meetings can be recorded. End-to-end encrypted meetings are also available. Northwestern Box is a file sharing service that provides individual, flexible online space for unlimited file storing or sharing. Both these resources can be used for this project.

The Minneapolis VA Health Care System (MVAHCS) is one of seven Regional Amputation Centers (RACs), the flagship facilities within the VA's Amputation System of Care. The MVAHCS is also one of five VA Polytrauma Rehabilitation Centers in the US. The prosthetics department (BU wing, Building 70) at the MVAHCS is a fully-equipped clinic including three full-time prosthetists and a number of support staff. Patient areas include fitting/exam rooms, clinical evaluation room, and a cast room. Support areas include orthotics and prosthetics (O&P) lab work-stations, and O&P fabrication supply storage. Lastly, staff and administrative areas include O&P lab staff office space. In total, the prosthetics fitting facility has over 4,200 square feet of space within the MVAHCS.

The prosthetics lab has a WillowWood OMEGA carver for creating positive limb models out of foam material, and a Provel thermoformer for creating plastic check sockets over the foam limb models. These systems can be used by the prosthetists at the MVAHCS to create check sockets for the proposed study. The prosthetics lab also has saws, drill presses, sanders, and other equipment that Dr. Hansen and his group already use for research and development projects related to prosthetics. Additionally, the prosthetics lab has all the necessary equipment needed to create multi-stage laminated sockets on-site for patients.

In the MVAHCS machine shop, we have lathes, a milling machine, saws, drill presses, and equipment needed to weld aluminum, titanium, and other metals. Additionally, the machine shop has a Stratasys FDM-400 rapid prototyping machine that can be used to quickly create three-dimensional parts if needed for this project.

Dr. Hansen has an office (4P-130) and four laboratories (4P-111, 4P-121, 4P-124, and 4P-131) in the medical center (same building as the prosthetics department, Building 70). Dr. Hansen also has a clinical research laboratory (3M-123, Building 70) where patients are seen for human subjects testing. Other study personnel are also equipped with computers and desks within the MVAHCS, with all the necessary software needed to perform the project.

The Italian Workers Compensation Authority (Istituto Nazionale per l'Assicurazione contro gli Infortuni sul Lavoro, INAIL) is one of the largest public, non-profit institutions in Italy. It has over 200 local offices, with headquarters in Rome, Piazzale Giulio Pastore 6.

INAIL activities range from work-related injury prevention, to injured workers' economic compensation, including the direct provision of medical care in addition to the standard care provided by the National Healthcare Services. In this framework, INAIL operates a Prosthetic Center with headquarters in Vigorso di Budrio, near Bologna, branches in Roma and Lamezia Terme, and territorial contact points in Milan, Venice, Naples, and Bari.

The site at Vigorso di Budrio was founded in 1961 with the specific mission to *"experiment and apply prostheses and prosthetic aids"*. It comprises four functional areas: prosthetic and orthotic production, delivery of inpatient and outpatient physical medicine and rehabilitation services, assistive devices provision, and research and education. At 20,000m^2 it is the largest clinical prosthetic facility worldwide.

The site Principle Investigator for this project, Dr. Cutti, is the Applied Research Manager within the prosthetic and orthotic production area. The production area sees the involvement of 4 engineers and over 190 certified prosthetists/orthotists and technicians, who coordinate their activities with a team of 8 physicians, 15 physical therapists, 2 social workers and 1 clinical psychologist.

The production area consists of six workshops: transtibial prostheses, transfemoral prostheses, insoles and footwear, orthoses, upper-limb prostheses, and silicone prostheses. The transtibial and transfemoral prosthesis workshops annual fittings tally 1200 and 800 prostheses, respectively.

All workshops are fully equipped for casting, rectification, vacuum forming, plastic and composite laminations, prosthetic component assembly and alignment, and initial gait training. All the typical prosthetic and orthotic machineries are available, including computer-controlled milling machines. The production area is also equipped with a state-of-the-art 7-axis robot (Ortis, Roboticom, Italy), computer aided design-computer aided manufacturing (CAD-CAM) software, and two fused deposition modelling (FDM) printers (WASP, Italy).

A fully equipped motion analysis laboratory is also part of the production area. Equipped with a Vicon optoelectronic system with 10 cameras, three Kistler force-plates, a 16 channel EMG wireless system, a Cosmed K5, Novel *pedar-X* and *pliance* sensors, StepWatch sensors, photocells, a Phantom high-speed camera, one Micro Gate Opto Gait motion analysis system, and a set of Xsens inertial measurement units.

MULTI-SITE RESEARCH

Organizational Chart

Figure 8 provides an overview of the organizations involved in the proposed multi-institutional study and the key members of the study team. Northwestern University will lead the study. Data will be collected by prosthetists at all three sites (Northwestern University, Minneapolis VA Health Care System and INAIL). INAIL personnel will provide the majority of data processing, while MVAHCS personnel will provide statistical analysis.

We have partnered with INAIL (based in Italy) because of their extensive experience in assessing the clinical feasibility of the Symphonie Aqua System^{TM} during prototype development.[30] Our colleagues at INAIL have amassed a depth of experience and understanding of the system that is not currently available in the US. To date, INAIL has experience with over 140 transfemoral and 100 transtibial successful fittings with the Symphonie Aqua System^{TM}. This experience will be shared across study sites, especially during our initial in-person training meeting. Additionally, INAIL has developed the technical competence required to support data analysis for this project given their experience scanning, processing and analyzing scans of residual limb casts/shapes.

Northwestern University is the lead site on the study. As the PI at Northwestern University, Dr. Fatone will lead the project, assuming administrative responsibility and coordinating all components of the project with collaborating sites. She will monitor progress at all sites by engaging in monthly teleconferences with site PIs. This will allow challenges and issues to be discussed, to ensure that protocols are being followed correctly, and that data quality is being maintained. Dr. Fatone will coordinate the necessary HRPO IRB approvals and DOD technical/fiscal reports.

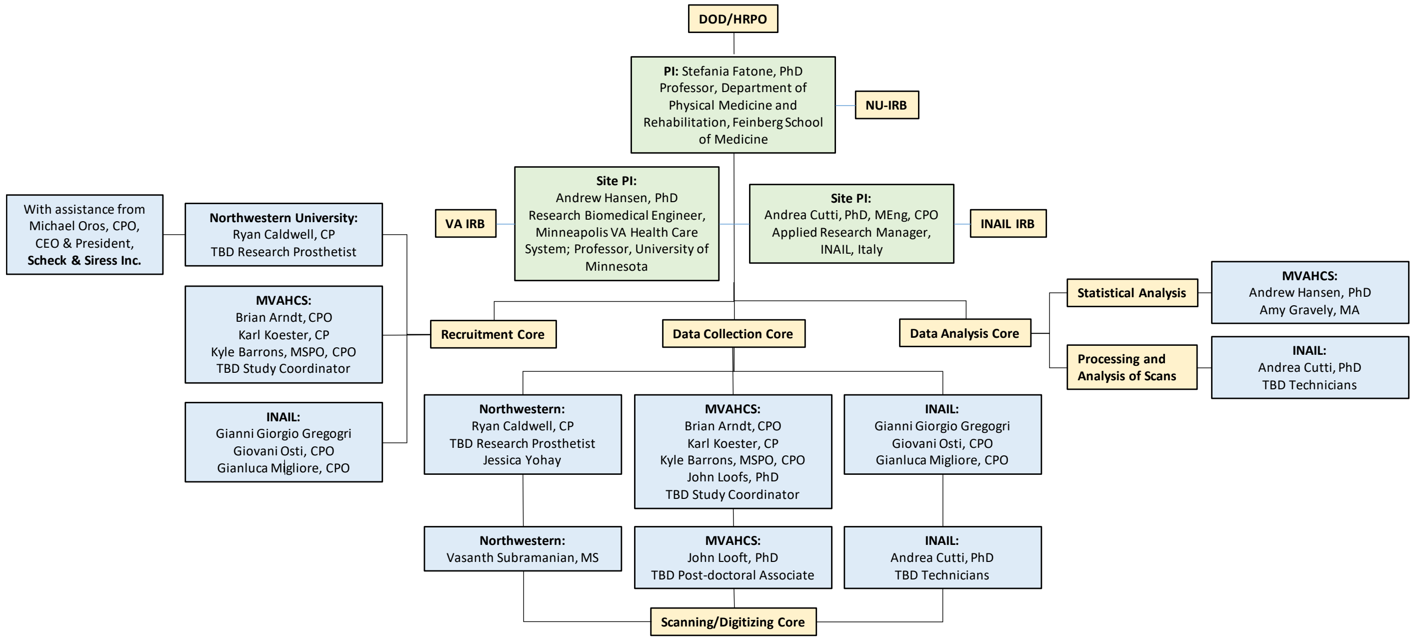

Figure 8 Organizational chart identifying key members of the study team within each of the core study domains and an outline of the governing structure of the proposed multi-institutional clinical trial.

REFERENCES

1. Geil MD. Consistency and accuracy of measurement of lower-limb amputee anthropometrics. *J Rehabil Res Dev*. 2005; 42: 131-40.
2. Morgan S, Friedly J, Amtmann D, Salem R and Hafner B. Cross-sectional assessment of factors related to pain intensity and pain interference in lower limb prosthesis users. *Arch Phys Med Rehabil*. 2017; 98: 105-13.
3. Desmond D, Gallagher P, Henderson-Slater D and Chatfield R. Pain and psychosocial adjustment to lower limb amputation amongst prosthesis users. *Prosthet Orthot Int*. 2008; 32.
4. Buis AW, Blair A, Convery P, Sockalingam S and McHugh B. Pilot study: data-capturing consistency of two trans-tibial casting concepts, using a manikin stump model: a comparison between the hands-on PTB and hands-off ICECAST compact concepts. *Prosthet Orthot Int*. 2003; 27: 100-6.
5. Convery P, Buis AW, Wilkie R, Sockalingam S, Blair A and McHugh B. Measurement of the consistency of patellar-tendon-bearing cast rectification. *Prosthet Orthot Int*. 2003; 27: 207-13.
6. Courtney A, Orendurff MS and Buis A. Effect of alignment perturbations in a trans-tibial prosthesis user: A pilot study. *J Rehabil Med*. 2016; 48: 396-401.
7. Sanders JE, Youngblood RT, Hafner BJ, et al. Effects of socket size on metrics of socket fit in trans-tibial prosthesis users. *Med Eng Phys*. 2017; 44: 32-43.
8. Gailey R, Kristal A, Lucarevic J, Harris S, Applegate B and Gaunaurd I. The development and internal consistency of the comprehensive lower limb amputee socket survey in active lower limb amputees. *Prosthet Orthot Int*. 2018; https://doi.org/10.1177/0309364618791620.
9. Commean PK, Smith KE, Cheverud JM and Vannier MW. Precision of surface measurements for below-knee residua. *Arch Phys Med Rehabil*. 1996; 77: 477-86.
10. Lee PV, Lythgo N, Laing S, Lavranos J and Thanh NH. Pressure casting technique for transtibial prosthetic socket fit in developing countries. *J Rehabil Res Dev*. 2014; 51: 101-10.

11. Hittenberger D and Carpenter K. A below knee vacuum casting technique. *Orthot Prosthet*. 1983; 37: 15-23.
12. Manucharian SR. An investigation of comfort level trend differences between the hands-on patellar tendon bearing and hands-off hydrocast transtibial prosthetic sockets. *J Prosthet Orthot*. 2011; 23: 124-40.
13. Shikh SS, Osman NA and Latif LA. Hydrostatic cast system: Pressure mapping of the stump-water interface. *The 3rd International Symposium on Biomedical Engineering*. 2008: 266-9.
14. Abu Osman N, Spence W, Solomonidis S and Weir A. A simple and low cost method of producing a prosthetic socket for trans-tibial amputees. *3rd IEEE Seminar on Appropriate Medical Technology for Developing Countries*. 2004, p. 33/1-/5.
15. Kahle J. Conventional and hydrostatic transtibial interface comparison. *J Prosthet Orthot*. 1999; 11: 85-91.
16. Selles RW, Janssens PJ, Jongenengel CD and Bussmann JB. A randomized controlled trial comparing functional outcome and cost efficiency of a total surface-bearing socket versus a conventional patellar tendon-bearing socket in transtibial amputees. *Arch Phys Med Rehabil*. 2005; 86: 154-61.
17. Safari M, Rowe P, McFadyen A and Buis A. Hands-off and hands-on casting consistency of amputee below knee sockets using magnetic resonance imaging. *Sci World J*. 2013; 13:http://dx.doi.org/10.1155/2013/486146
18. Wu Y, Casanova H, Smith WK, Edwards M and Childress DS. Technical Note: CIR sand casting system for trans-tibial socket. *Prosthet Orthot Int*. 2003; 27: 146-52.
19. Murdoch G. The "Dundee Socket" - A total contact socket for the below-knee amputation. *Orthot Prosthet*. 1965; 19: 231-4.
20. Convery P and Buis A. Socket/stump interface dynamic pressure distribution recorded during the prosthetic stance phase of gait of a trans-tibial amputee wearing a hydrocast socket. *Prosthet Orthot Int*. 1999; 23: 107-12.
21. Laing S, Lythgo N, Lavranos J and Lee PVS. Transtibial prosthetic socket shape in a developing country: A study to compare initial outcomes in pressure cast hydrostatic and patella tendon bearing designs. *Gait Posture*. 2017; 58: 363-8.
22. Goh JCH, Lee PVS and Chong SY. Stump/socket pressure profiles of the pressure cast prosthetic socket. *Clin Biomech*. 2003; 18: 237-43.
23. Engsberg J, Sprouse W, Uhrich M, Ziegler B and Luitjohan F. Comparison of rectified and unrectified sockets for transtibial amputees. *J Prosthet Orthot*. 2006; 18: 1-7.
24. Goh J, Lee P and Chong S. Comparative study between patellar-tendon-bearing and pressure cast prosthetic sockets. *J Rehabil Res Dev*. 2004; 41: 491-502.
25. Buis A, Kamyab M, Hillman S, Murray K and McGarry A. A preliminary evaluation of a hydro-cast trans-femoral socket, a proof of concept. *Prosthet Orthot Open J*. 2017; 1: 1-9.
26. Whiteside S, Allen M, Bick J, et al. Practice analysis of certified practitioners in the disciplines of orthotics and prosthetics. Alexandria, VA: American Board for Certification in Orthotics and Prosthetics, Inc., 2015.
27. Safari MR and Meier MR. Systematic review of effects of current transtibial prosthetic socket designs-Part 1: qualitative outcomes. *J Rehabil Res Dev*. 2015; 52: 491-508.
28. Safari MR and Meier MR. Systematic review of effects of current transtibial prosthetic socket designs-Part 2: quantitative outcomes. *J Rehabil Res Dev*. 2015; 52: 509-26.
29. Redhead R. Total surface bearing self suspending above-knee sockets. *Prosthet Orthot Int*. 1979; 3: 126-36.
30. Cutti A, Osti M, Migliore E, Cardin D, Ventruoli F and Verni G. Clinical effectiveness of a novel hydrostatic casting method for transfemoral amputees: results from the first 64 patients. *American Orthotic and Prosthetic Association*. Vancouver, BC, Canada, September 26-29, 2018.

31. Kahle JT and Highsmith MJ. Transfemoral sockets with vacuum-assisted suspension comparison of hip kinematics, socket position, contact pressure, and preference: ischial containment versus brimless. *J Rehabil Res Dev*. 2013; 50: 1241-52.
32. Kahle JT and Highsmith MJ. Transfemoral interfaces with vacuum assisted suspension comparison of gait, balance, and subjective analysis: Ischial containment versus brimless. *Gait Posture*. 2014; 40: 315-20.
33. Fatone S and Caldwell R. Northwestern University Flexible Subischial Vacuum Socket for persons with transfemoral amputation: Part 1 description of technique. *Prosthet Orthot Int*. 2017; 41: 237-45.
34. Fatone S and Caldwell R. Northwestern University Flexible Subischial Vacuum Socket for persons with transfemoral amputation: Part 2 Description and preliminary evaluation. *Prosthet Orthot Int*. 2017; 41: 246-50.
35. Fatone S, Johnson W, Tran L, Tucker K, Mowrer C and Caldwell R. Quantification of rectifications for Northwestern University Flexible Sub-Ischial Vacuum (NU-FlexSIV) Socket. *Prosthet Orthot Int*. 2017; 41: 251-7.
36. Caldwell R and Fatone S. Technique modifications for a suction suspension version of the Northwestern University Flexible Sub-Ischial Vacuum socket: The Northwestern University Flexible Sub-Ischial Suction socket. *Prosthet Orthot Int*. 2018; 0: 0309364618798869.
37. Storey M and Thomas D. Sub-Ischial transfemoral socket design. *Australian Orthotic and Prosthetic Association Congress*. Melbourne, Australia, October 6-8, 2016.
38. Sasaki S and Tonei K. Diffusion of NU-FlexSIV Socket in Japan 2017. *25th Scientific Meeting of the Japanese Academy of Prosthetists and Orthotists*. Sapporo, Japan, July 21-22, 2018.
39. Mulroy-Lang S. Overview of the NU-FlexSIV method with case studies. *New Zealand Orthotics and Prosthetics Association*. Auckland, New Zealand, June 1-2, 2018.
40. Mulroy S and Howells C. Optimising outcomes: Subischial socket design with Unity, Prof-Flex family, updated RHEO Knee XC features, and mechanical knee considerations. *Australian Orthotic and Prosthetic Association Congress*. Australia, October 5, 2017.
41. Imbeault V and Jette S. Experiences cliniques avec emboiture Nu-Flex SIV socket. *Quebec Orthotics and Prosthetics Conference*. Montreal, Canada, 2017.
42. Mazzone B, Yoder A, Zalewski B, Wyatt M and Sheu R. Comprehensive treatment strategy for chronic low back pain in a patient with bilateral transfemoral amputations integrating changes in prosthetic socket design. *J Prosthet Orthot*. 2017; 29: 190-7.
43. Hanspal RS, Fisher K and Nieveen R. Prosthetic socket fit comfort score. *Disabil Rehabil*. 2003; 25: 1278-80.
44. Fatone S, Dillon M, Stine R and Tillges R. Coronal plane socket stability during gait in persons with unilateral transfemoral amputation: pilot study. *J Rehabil Res Dev*. 2015; 51: 1217-28.
45. Fatone S, Yohay J and Caldwell R. Change in residual limb size over time in the NU-FlexSIV socket: A case study. *Prosthet Orthot Int*. 2018: 309364618775445.
46. Besl P and Mckay N. A method for registration of 3D shapes. *IEEE Trans Pattern Anal Mach Intell* 1992; 14: 239-56.
47. Zhang Z. Iterative point matching for registration of free-form curves and surfaces. *Int J Comput Vision*. 1994; 13: 119–52.
48. Gailey RS, Roach KE, Applegate EB, et al. The amputee mobility predictor: an instrument to assess determinants of the lower-limb amputee's ability to ambulate. *Arch Phys Med Rehabil*. 2002; 83: 613-27.
49. Mazzone B, Yoder A, Zalewski B, Wyatt M and Sheu R. Comprehensive treatment strategy for chronic low back pain in a patient with bilateral transfemoral amputations integrating changes in prosthetic socket design. *J Prosthet Orthot*. 2017; 29: 190-7.

50. Fatone S, Caldwell R and Major M. Effect of interface components on residual limb weight-bearing tolerance in the Northwestern University Flexible Sub-Ischial Vacuum (NU-FlexSIV) Socket. *World Congress of the International Society for Prosthetics and Orthotics*. Cape Town, South Africa, May 8-11, 2017.
51. Hafner BJ, Morgan SJ, Askew RL and Salem R. Psychometric evaluation of self-report outcome measures for prosthetic applications. *J Rehabil Res Dev*. 2016; 53: 797-812.
52. Adams P, Hendershot G and Marano M. Current estimates from the National Health Interview Survey, 1996. *Vital Health Stat*. 1999; 10(200).
53. Hardin J and Hilbe J. *Generalized Estimating Equations*. 2nd ed. Boca Raton, FL: CRC Press Taylor & Francis Group, 2012.
54. Hanspal RS, Fisher K and Nieveen R. Prosthetic socket fit comfort score. *Disabil Rehabil*. 2003; 25: 1278-80.
55. Heinemann AW, Connelly L, Ehrlich-Jones L and Fatone S. Outcome instruments for prosthetics: clinical applications. *Phys Med Rehabil Clin North Am*. 2014; 25: 179-98.
56. Jeste DV, Palmer BW, Appelbaum PS and et al. A new brief instrument for assessing decisional capacity for clinical research. *Archives of General Psychiatry*. 2007; 64: 966-74.
57. Deathe AB, Wolfe DL, Devlin M, Hebert JS, Miller WC and Pallaveshi L. Selection of outcome measures in lower extremity amputation rehabilitation: ICF activities. *Disabil Rehabil*. 2009; 31: 1455-73.
58. Resnik L and Borgia M. Reliability of outcome measures for people with lower-limb amputations: distinguishing true change from statistical error. *Phys Ther*. 2011; 91: 555-65.

Appendix A: Surveys, Questionnaires, and Other Data Collection Instruments

Socket Comfort Score[43]

To assess ***hypothesis 3.1*** that standing hydrostatic pressure casting with a water cylinder improves socket comfort as compared to hand casting, a blinded assessor will administer the Socket Comfort Score during Visit 3 in full weight bearing for each socket before and after the subject and prosthetist have made any modifications needed to achieve a satisfactory initial fit. Order in which sockets are fit/assessed will be randomized. Socket comfort is the primary outcome of the study, as we believe it is most important to the prosthesis user and the variable for which we powered the study. Additionally, recent work by Sanders et al.[7] indicated that Socket Comfort Score[43] is a viable metric for detection of differences in socket fit among persons with transtibial amputation and our own previous work showed the same sensitivity in persons with transfemoral amputation.[44]

Pain-measurement methods such as the numerical rating scale (NRS) were used by Hanspal et al.[43] as models for quantifying and communicating socket comfort. The Socket Comfort Score asks the standard question, *"On a 0 to 10 scale, if 0 represents the most uncomfortable socket fit you can imagine, and 10 represents the most comfortable socket fit, how would you score the comfort of the socket fit of your artificial limb at the moment?"*[43] The developers of this measure reported interrater reliability, criterion-related validity, sensitivity to change, and utility in clinical practice in 44 consecutive lower limb amputees.[54] As part of development of the measure, a treating prosthetist collected Socket Comfort Score responses three times during the course of a single day, as did an independent prosthetist and a physician. Their ratings were highly correlated (Kendall tau 0.97–0.98, p<.001). Socket Comfort Score responses were significantly correlated with assessments of socket fit by the treating prosthetist and with residual limb health by a physician (Kendall tau 0.51 and 0.48, p<.001). There was sensitivity to change, with 76% (n = 22) of adjusted sockets demonstrating a significant improvement in the Socket Comfort Score of 1 to 5 points (Wilcoxon z = 74.16, p<.001). The Socket Comfort Score is a simple, easy-to-administer measure that allows clinicians to quantify change in socket fit rather than relying on descriptive terms.[55] More recently, Hafner et al.[51] reported that the test-retest Intraclass Correlation Coefficients (ICC) for the Socket Comfort Score administered to lower limb amputees 2-3 days apart ranged from 0.63 to 0.79, depending on mode of administration, indicating that the Socket Comfort Score is appropriate for group-level comparisons when administered in a single mode.

Hence, Socket Comfort Score at initial fit will be compared between sockets fabricated with each casting approach using General Estimating Models (GEE) models, which account for both correlation and any type of outcome variable. The simplest GEE model will resemble a paired t-test, but if paired t-test assumptions are not met, GEE models allow us to accommodate almost any distribution for the dependent variable (ordinal, dichotomous, gamma, and so forth). These will be fit in SAS PROC GENMOD software (SAS Institute Inc., Cary, NC).

Amputee Mobility Predictor (AMP)[48]

Once enrolled, subjects will be evaluated at Visit 1 to define the study population. One of the characteristics to be defined is functional mobility (i.e., Medicare Functional Classification Level or K-Level),[48] for which we will use the Amputee Mobility Predictor, AMP)[48]. Since all participants must be current prosthesis users in order to be included in the study, the AMPPRO will be administered (i.e., with prosthesis) for this study.

The AMP measures ambulatory potential of people with lower limb amputations with (AMPPRO) and without a prosthesis (AMPnoPro); published psychometric information is available only for the AMPPRO.[48] The AMP consists of 21 items that evaluate transfers, sitting and standing balance, and gait skills. It demonstrates good inter-rater and intra-rater reliability,[48] and concurrent validity has been established with the 6 Minute Walk Test (6MWT) and Amputee Activity Survey (AAS).[48] The AMPPRO predicts distance walked in 6 minutes[48] and distinguishes

Medicare Functional Classification Levels (k-levels),[48] although wide score distributions preclude cutoff scores.[57]

Resnik and Borgia[58] recruited a convenience sample of 44 lower limb prosthesis users with transfemoral, knee disarticulation, and transtibial amputations, and administered the AMPPRO twice within one week. The average AMPPRO score was 40 ± 4 (range: 28–45) on the first occasion and 41 ± 4 (range: 29–46) on the second occasion. AMPPRO total Intraclass Correlation Coefficient (ICC) was 0.88 (95% Confidence Interval of 0.79–0.93), comparable with previous reports of 0.96 to 0.98.[48] Standard Error of the Mean (SEM) was 1.5 and Minimal Detectable Change (MDC90) was 3.4.[58]

University of California San Diego Brief Assessment of Capacity to Consent (UBACC)[56]

Decisional capacity to consent to clinical research will be assessed before a signature is obtained on the consent form using the University of California San Diego Brief Assessment of Capacity to Consent (UBACC).[56] The UBACC provides a brief and easy-to-use yet reliable and validated method by which investigators may document that at least a basic level of comprehension of key elements of the project protocol is present prior to enrollment and may identify individuals for whom a more thorough assessment of decisional capacity is needed. The UBACC is a 10-item scale (with 4 items examining understanding, 5 for appreciation, and 1 for reasoning) that can be administered by research staff with a minimum Bachelors level education and typically requires less than 5 minutes to administer.

The UBACC is administered after the participant has reviewed the consent form in detail. The investigator explains that they are going to ask a few brief questions about the study. Potential participants generally have a copy of the consent form available to them during the consent process and therefore do not have to rely solely on their ability to memorize the protocol details when giving consent to enroll. Consequently, subjects being screened with the UBACC are permitted to refer to the consent form when answering questions. However, the participant is encouraged to explain the information relevant to each item in their own words to ensure that their responses reflect genuine comprehension, not merely an ability to read or repeat the consent form.

Each item of the UBACC is scored on a scale of 0 to 2 points, with 0 reflecting a clearly incapable response and 2 indicating a clearly capable response. A score of 1 or 0 on any particular item may alert investigators to the need for further assessment of decisional capacity. Total scores can thus range from 0 to 20. Prior to the initiation of the study, the PI must: (1) examine the UBACC questions and determine which of the 10 are essential for consent to the specific protocol, and (2) prepare a list of answers for his or her specific study that will receive a score of 1 or 2 on each item.

The UBACC was found to have good internal consistency (Cronbach's alpha = 0.76 and 0.77), inter-rater reliability (intraclass correlation coefficients = 0.84 and 0.98), concurrent validity with the MacArthur Competency Assessment Tool for Clinical Research (MacCAT-CR), high sensitivity (89%), and acceptable specificity (100%) in older and middle aged persons with schizophrenia and healthy controls.[56]
